# Supplementary figures and images for: High variability within pet foods prevents the identification of native species in pet cats’ diets using isotopic evaluation
Source: PeerJ. 2020 Jan 22;8:e8337. doi: 10.7717/peerj.8337 (PMC6982418; doi:10.7717/peerj.8337)

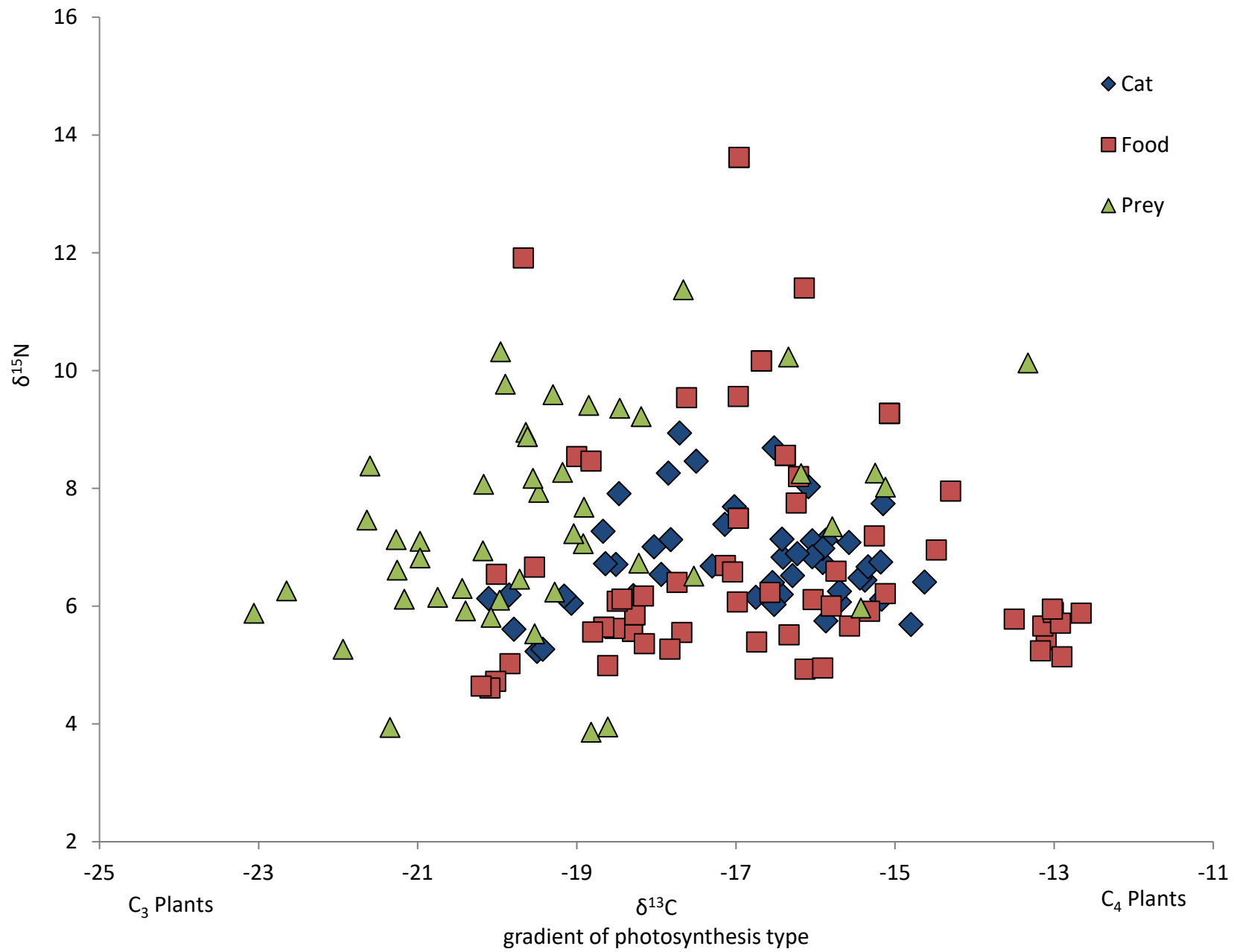

Supplement: Figure S1 — Plot shows cats and TEF adjusted food sources from the southeastern U.S. Raw isotope values for food and prey are adjusted by an enrichment factor of +2.6‰δ13C and +1.9‰δ15N. [file peerj-08-8337-s001.pdf]

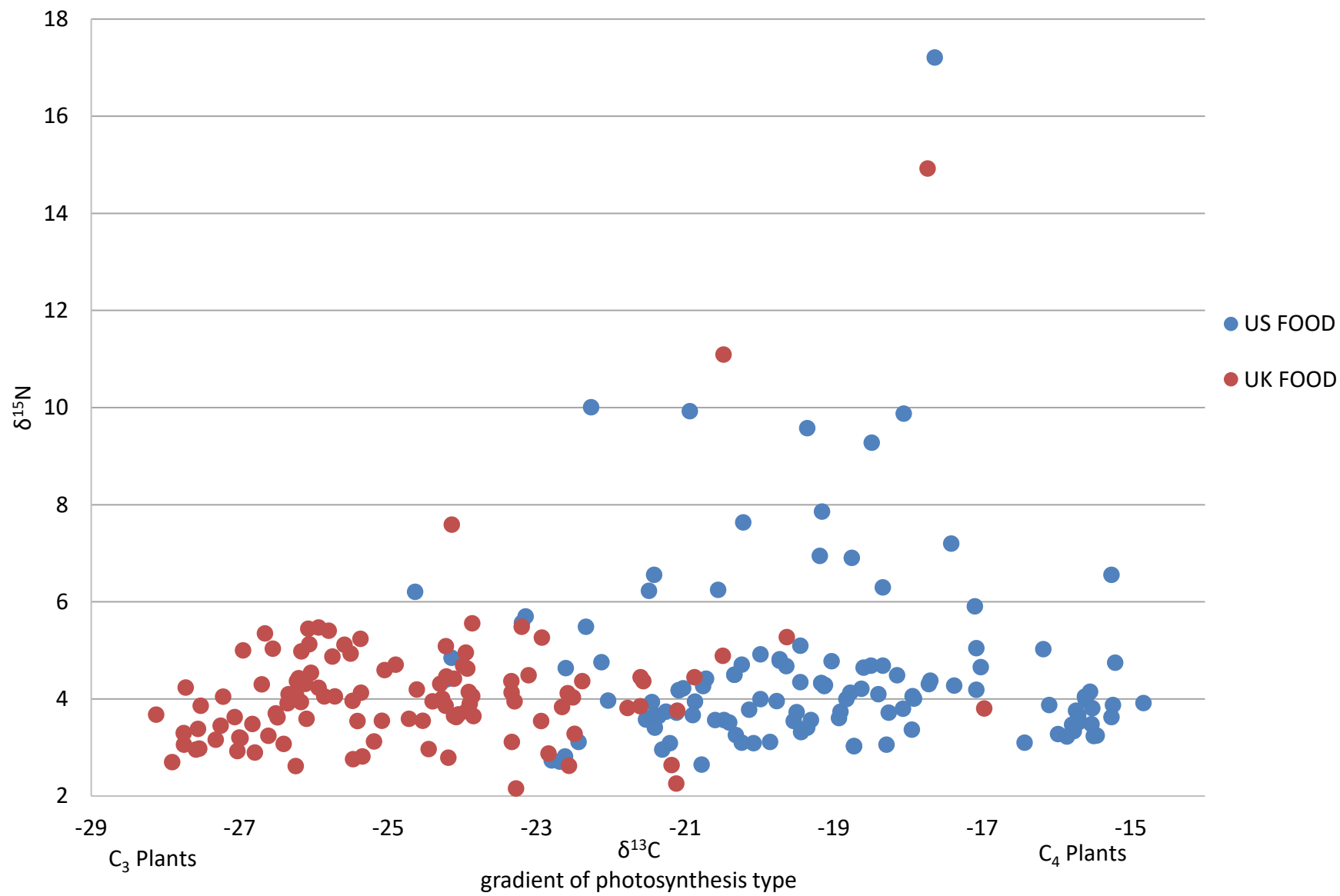

Supplement: Figure S2 [file peerj-08-8337-s002.pdf]

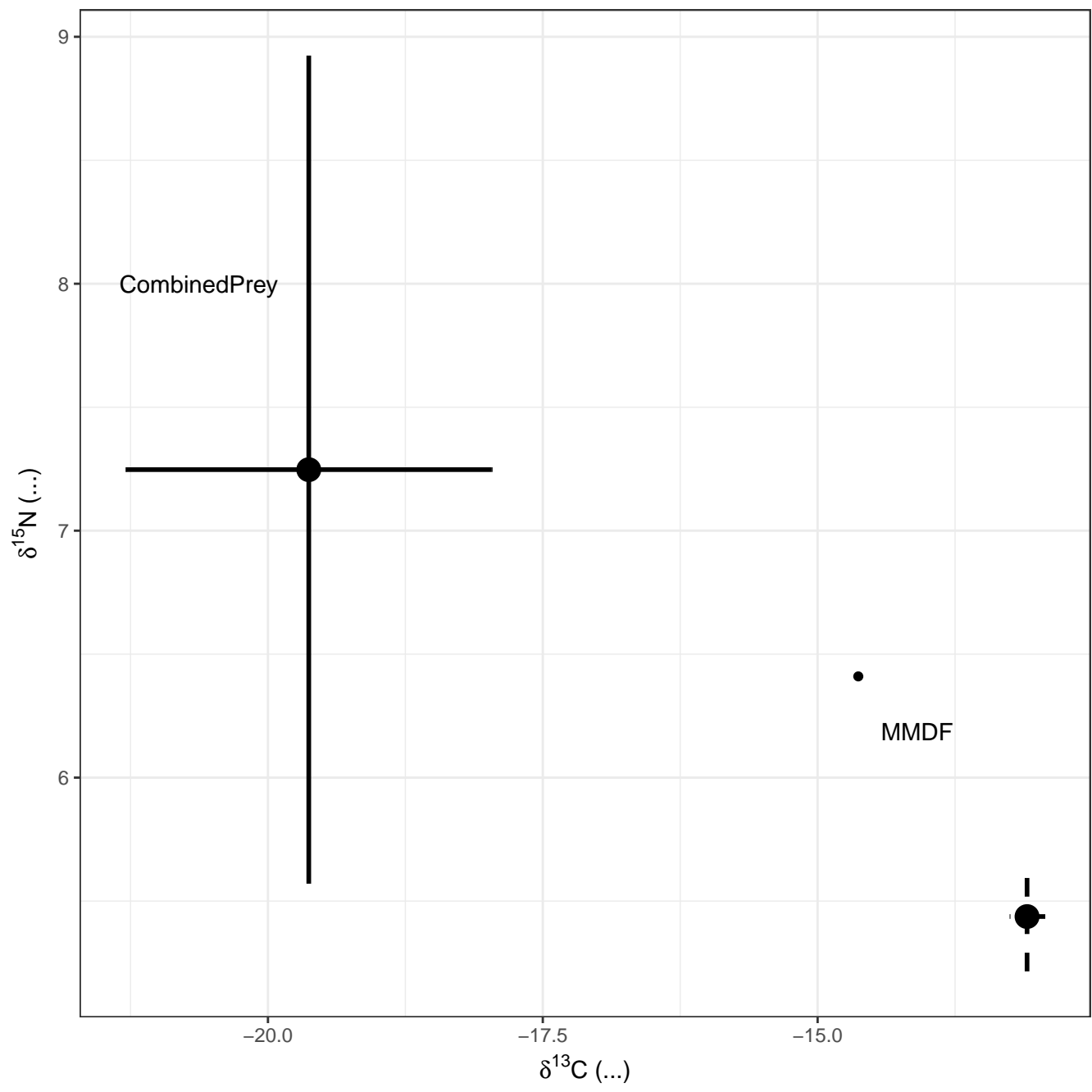

Supplement: Supplemental Information 2 — Contains isospace plot generated in MixSIAR for each cat in analysis. [file peerj-08-8337-s007.zip › Isospace plots/Amber_isospace combadj.pdf]

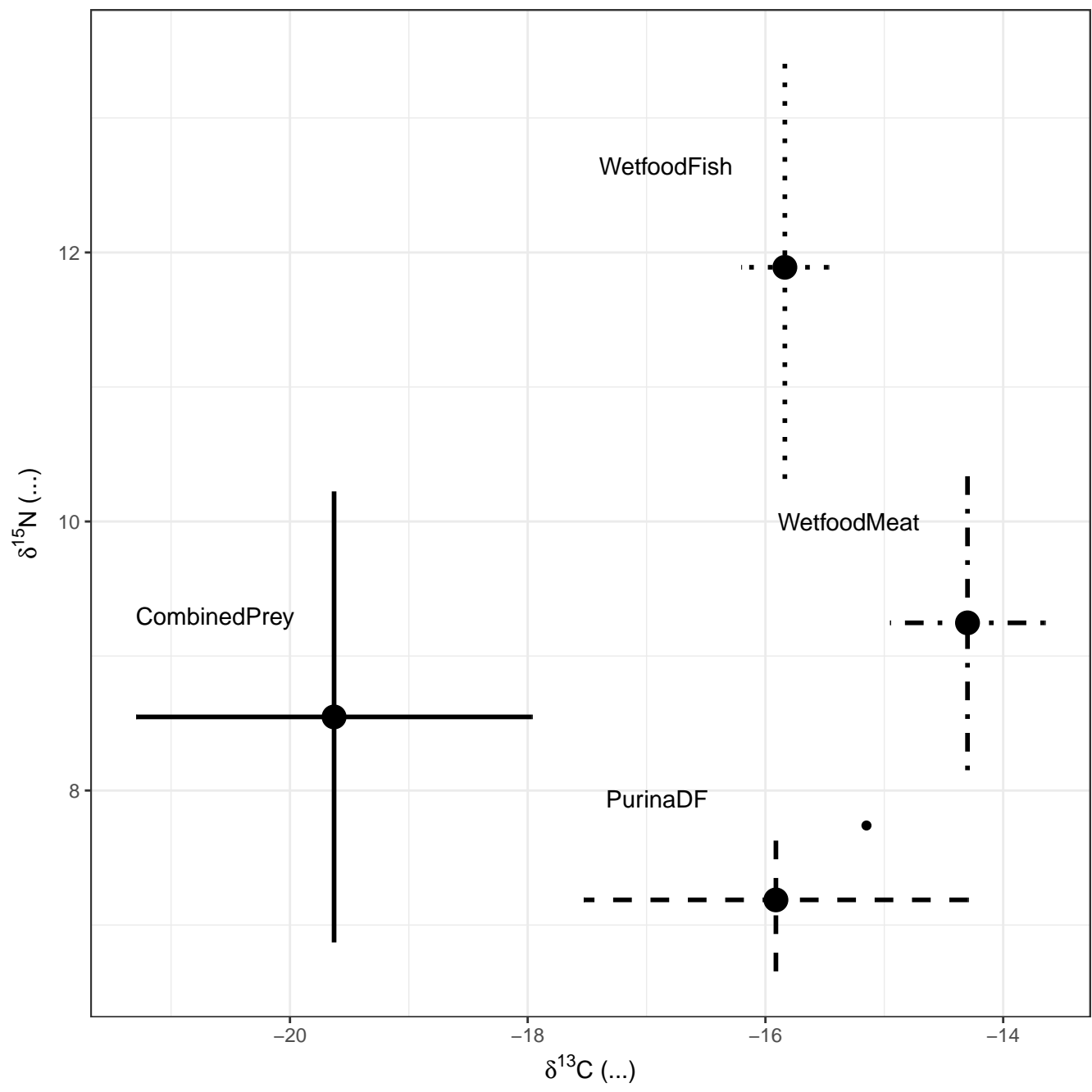

Supplement: Supplemental Information 2 — Contains isospace plot generated in MixSIAR for each cat in analysis. [file peerj-08-8337-s007.zip › Isospace plots/Banjo_isospace combadj.pdf]

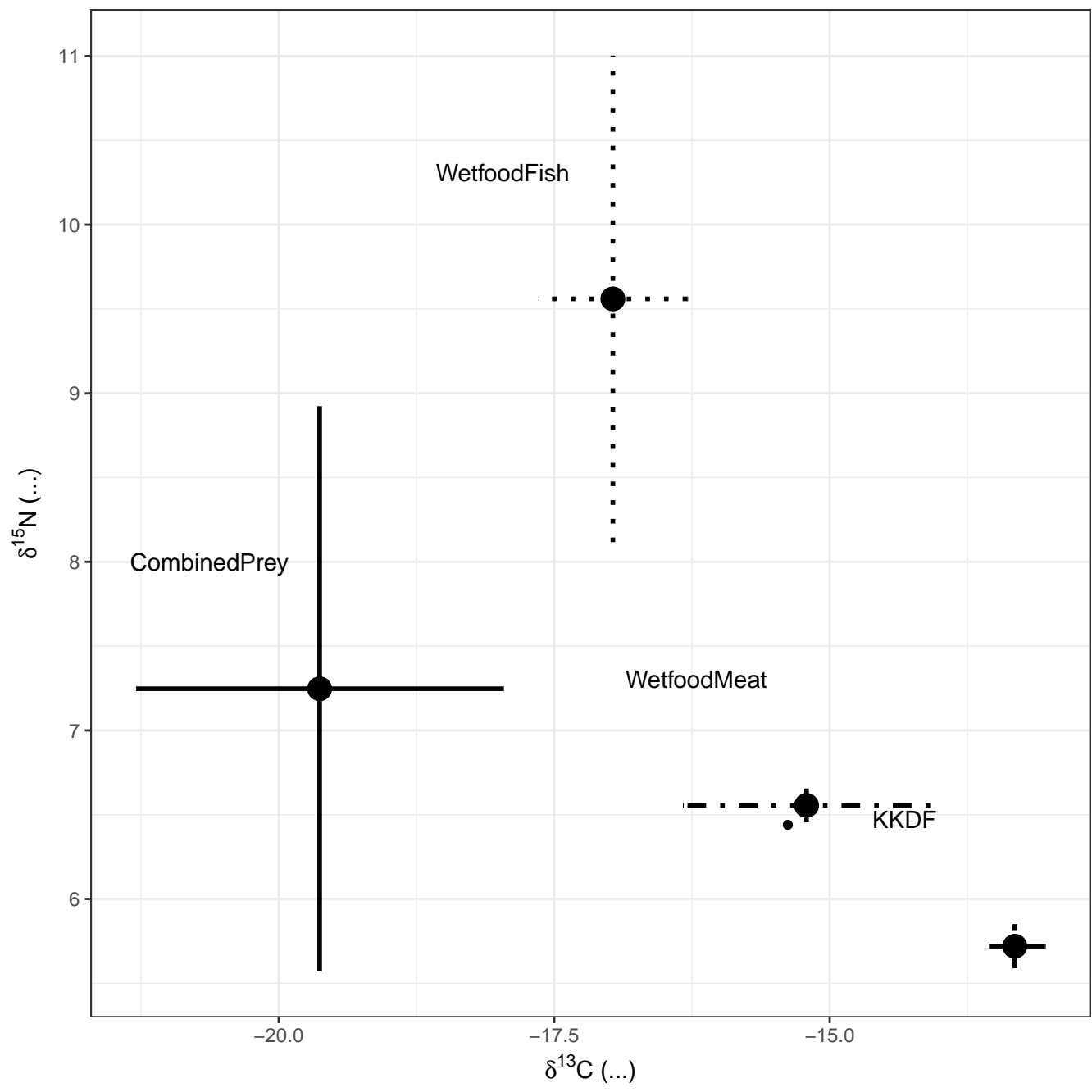

Supplement: Supplemental Information 2 — Contains isospace plot generated in MixSIAR for each cat in analysis. [file peerj-08-8337-s007.zip › Isospace plots/Beatle_isospace_combadj.pdf]

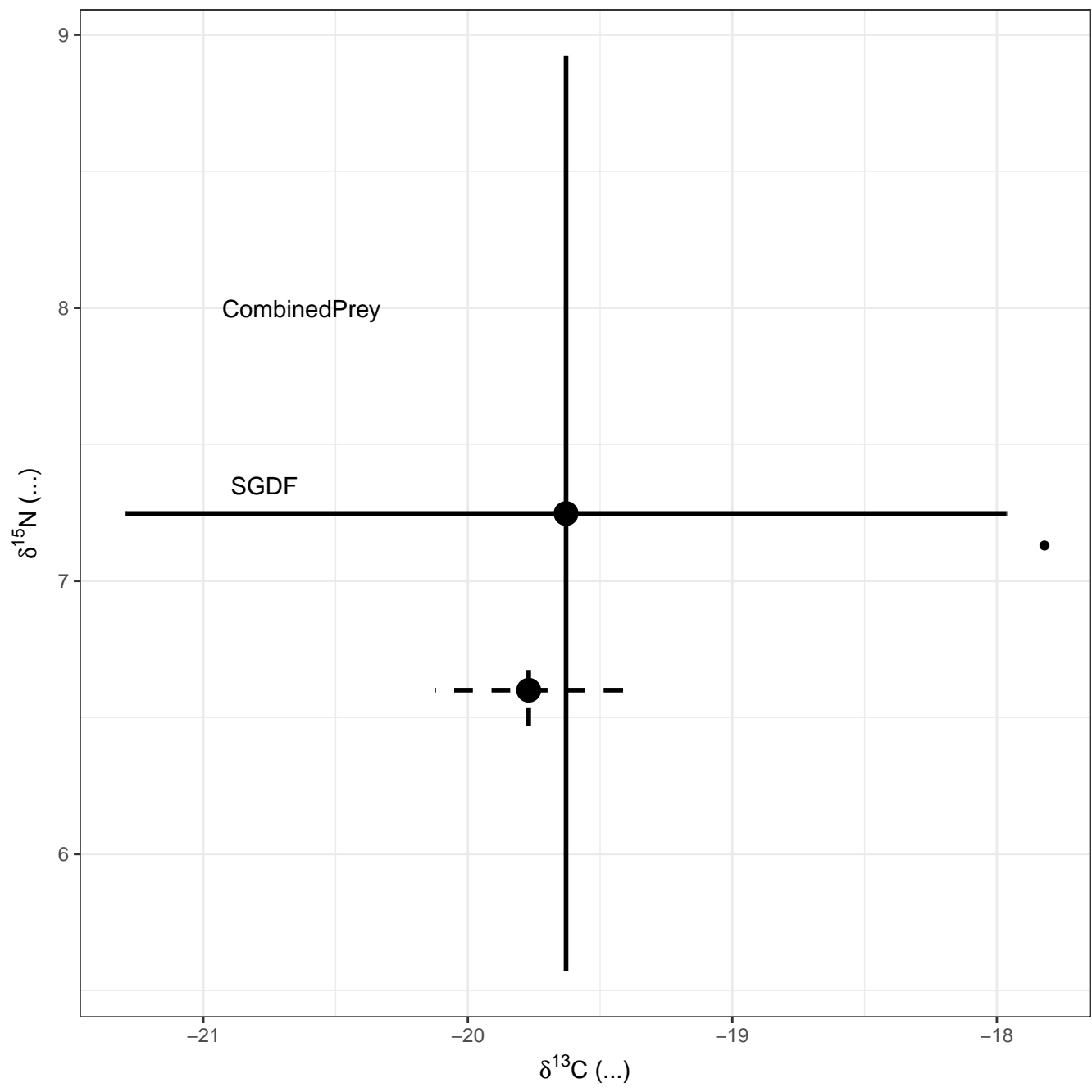

Supplement: Supplemental Information 2 — Contains isospace plot generated in MixSIAR for each cat in analysis. [file peerj-08-8337-s007.zip › Isospace plots/Bella_isospace combadj.pdf]

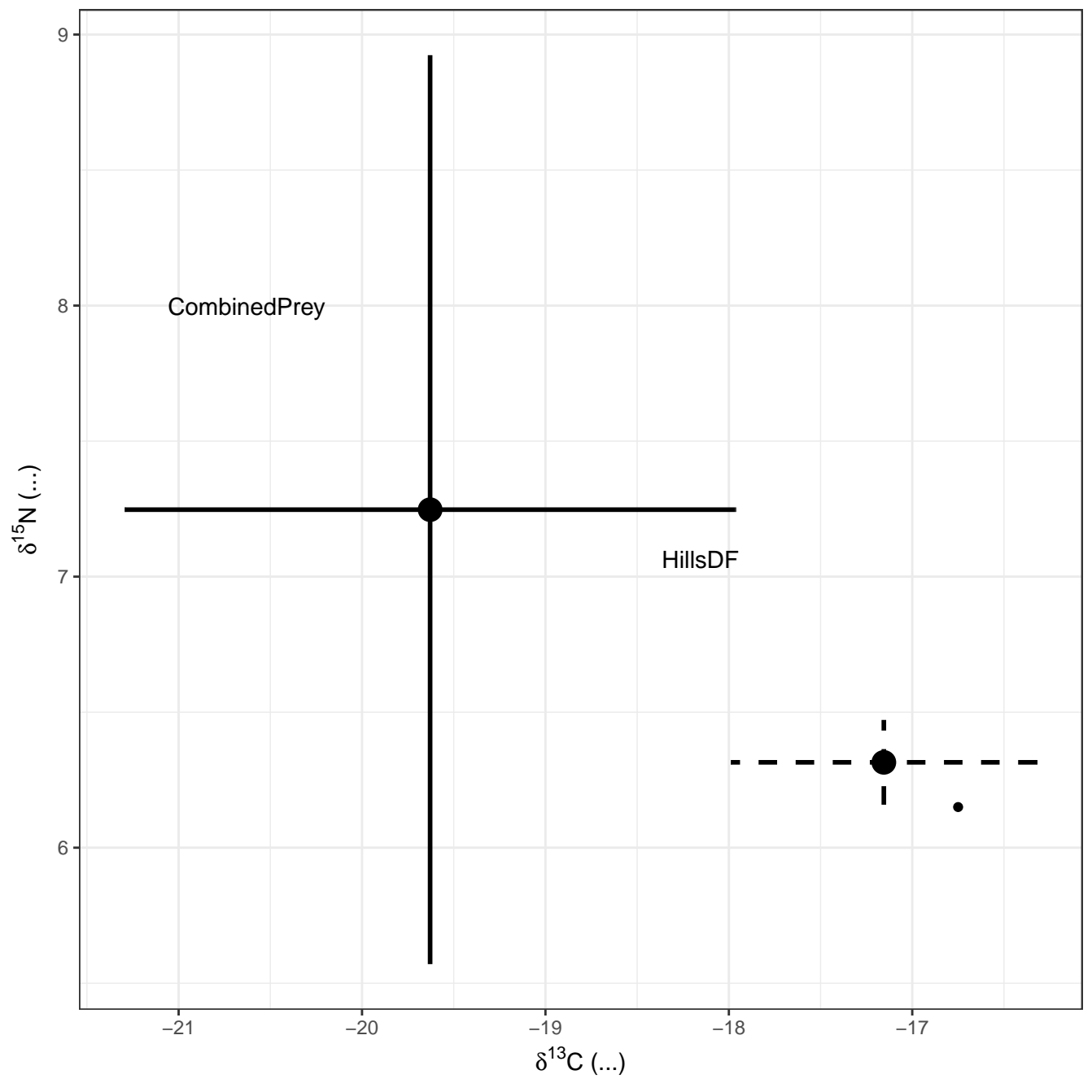

Supplement: Supplemental Information 2 — Contains isospace plot generated in MixSIAR for each cat in analysis. [file peerj-08-8337-s007.zip › Isospace plots/Boris_isospace combadj.pdf]

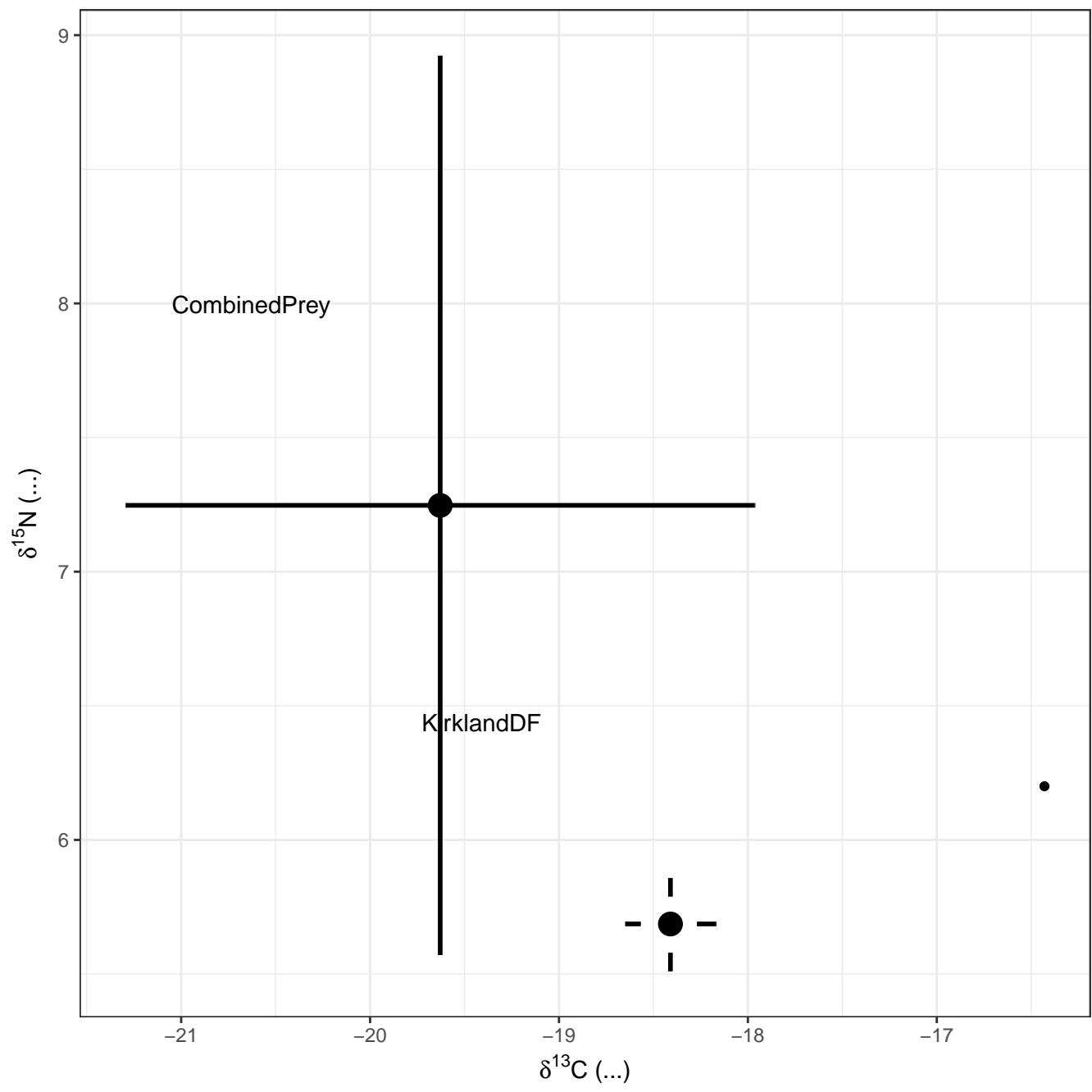

Supplement: Supplemental Information 2 — Contains isospace plot generated in MixSIAR for each cat in analysis. [file peerj-08-8337-s007.zip › Isospace plots/CatnissE_isospace combadj.pdf]

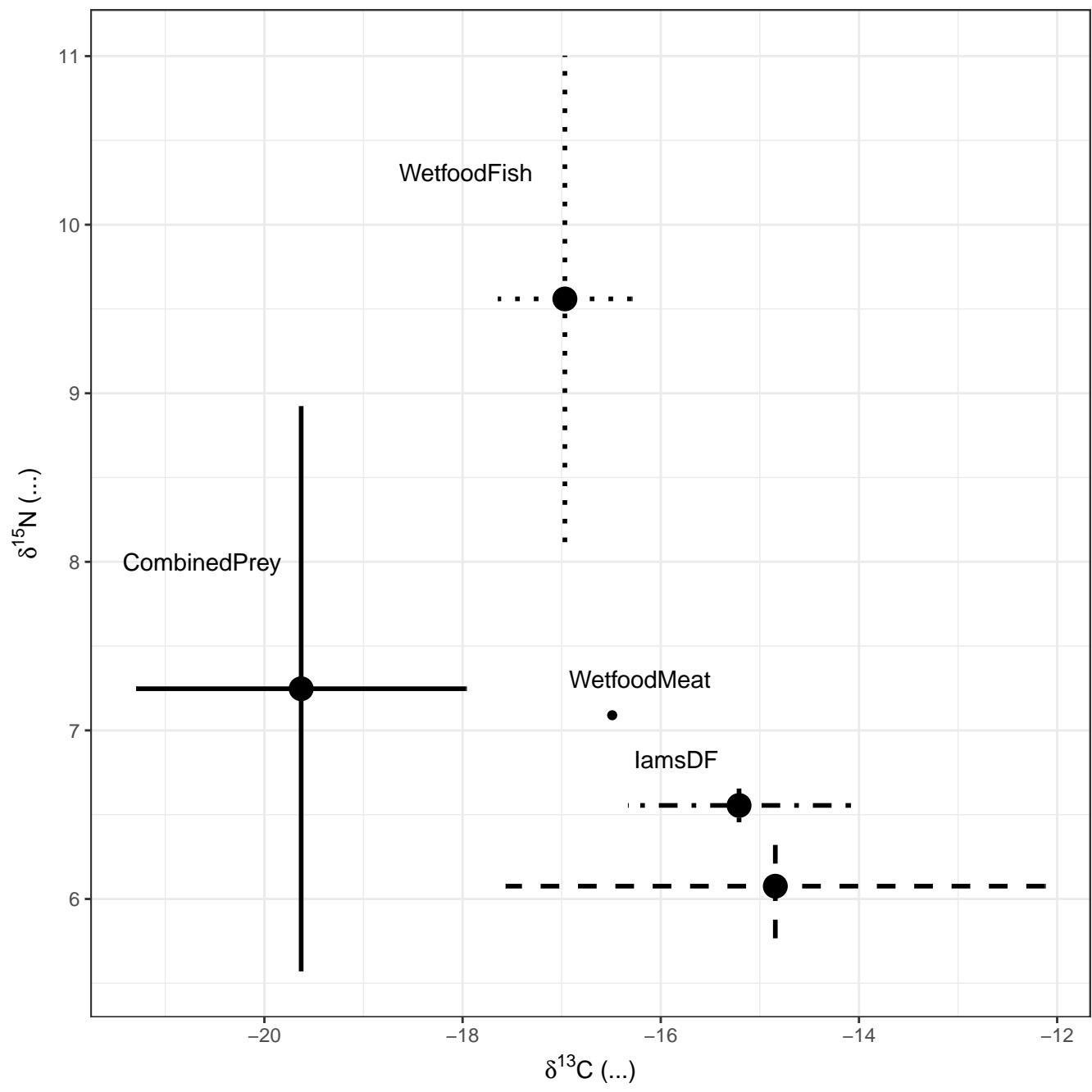

Supplement: Supplemental Information 2 — Contains isospace plot generated in MixSIAR for each cat in analysis. [file peerj-08-8337-s007.zip › Isospace plots/Cayden_isopace combadj.pdf]

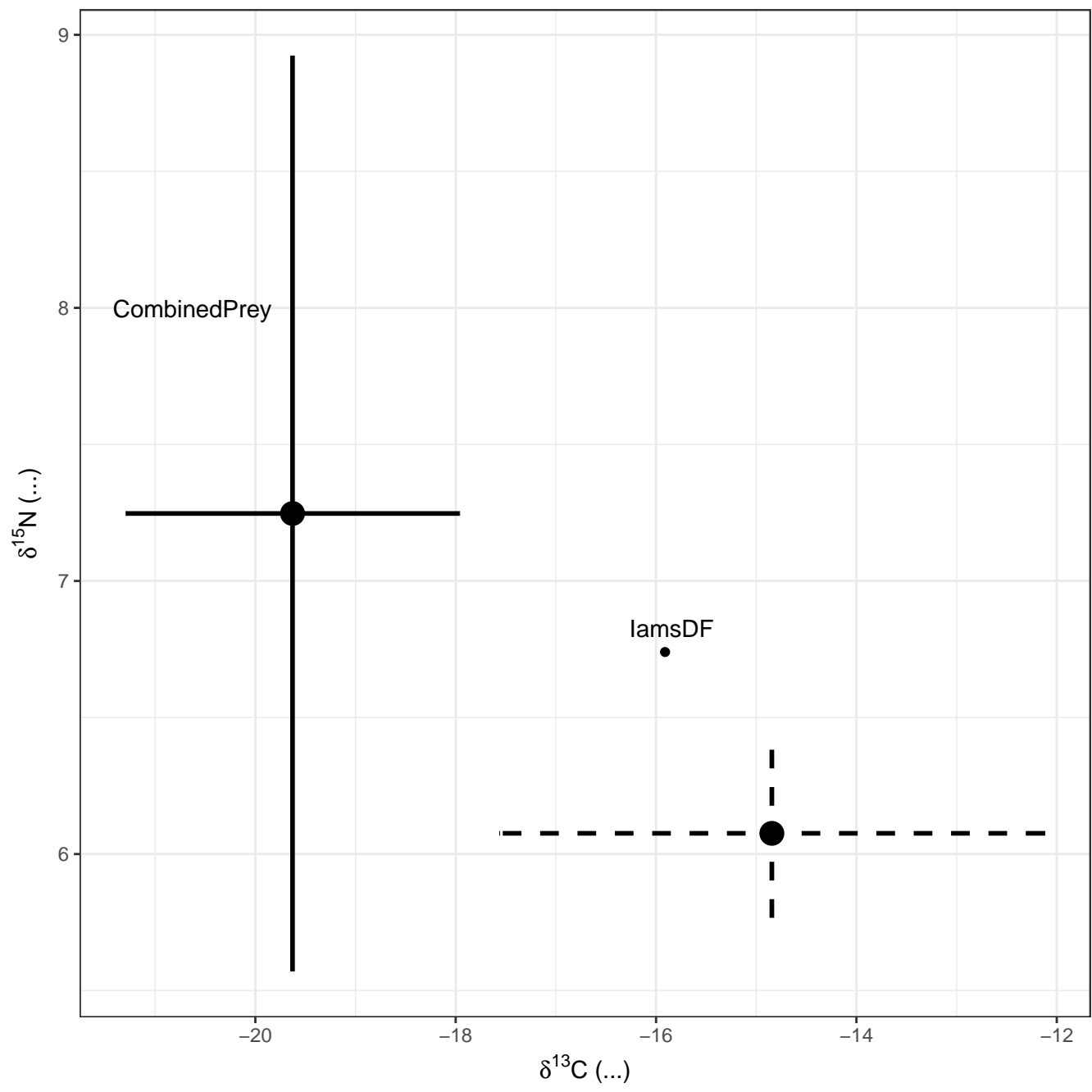

Supplement: Supplemental Information 2 — Contains isospace plot generated in MixSIAR for each cat in analysis. [file peerj-08-8337-s007.zip › Isospace plots/Climber_isospace combadj.pdf]

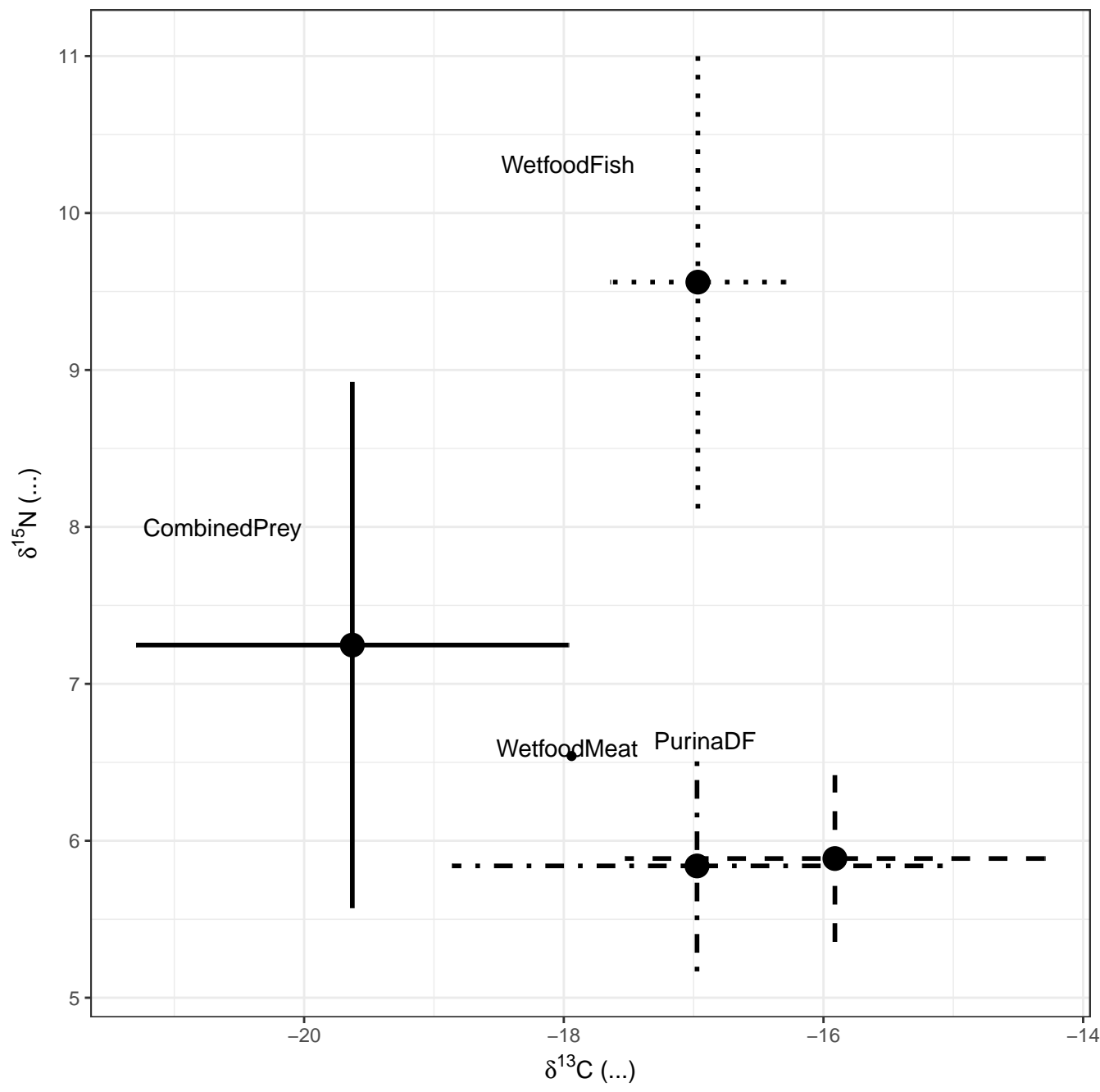

Supplement: Supplemental Information 2 — Contains isospace plot generated in MixSIAR for each cat in analysis. [file peerj-08-8337-s007.zip › Isospace plots/CptnAmerica_isospace combadj.pdf]

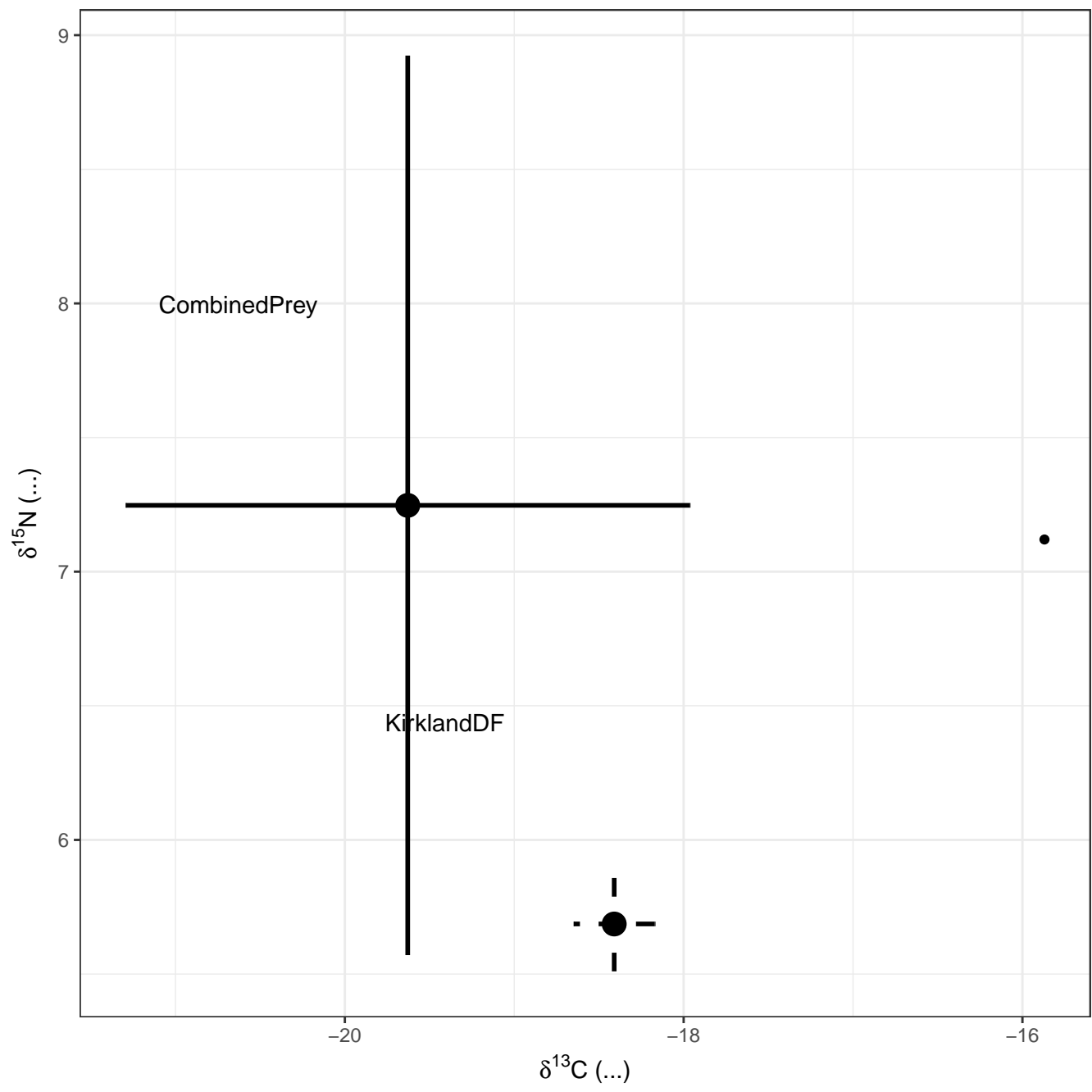

Supplement: Supplemental Information 2 — Contains isospace plot generated in MixSIAR for each cat in analysis. [file peerj-08-8337-s007.zip › Isospace plots/DCarlos_isospace combadj.pdf]

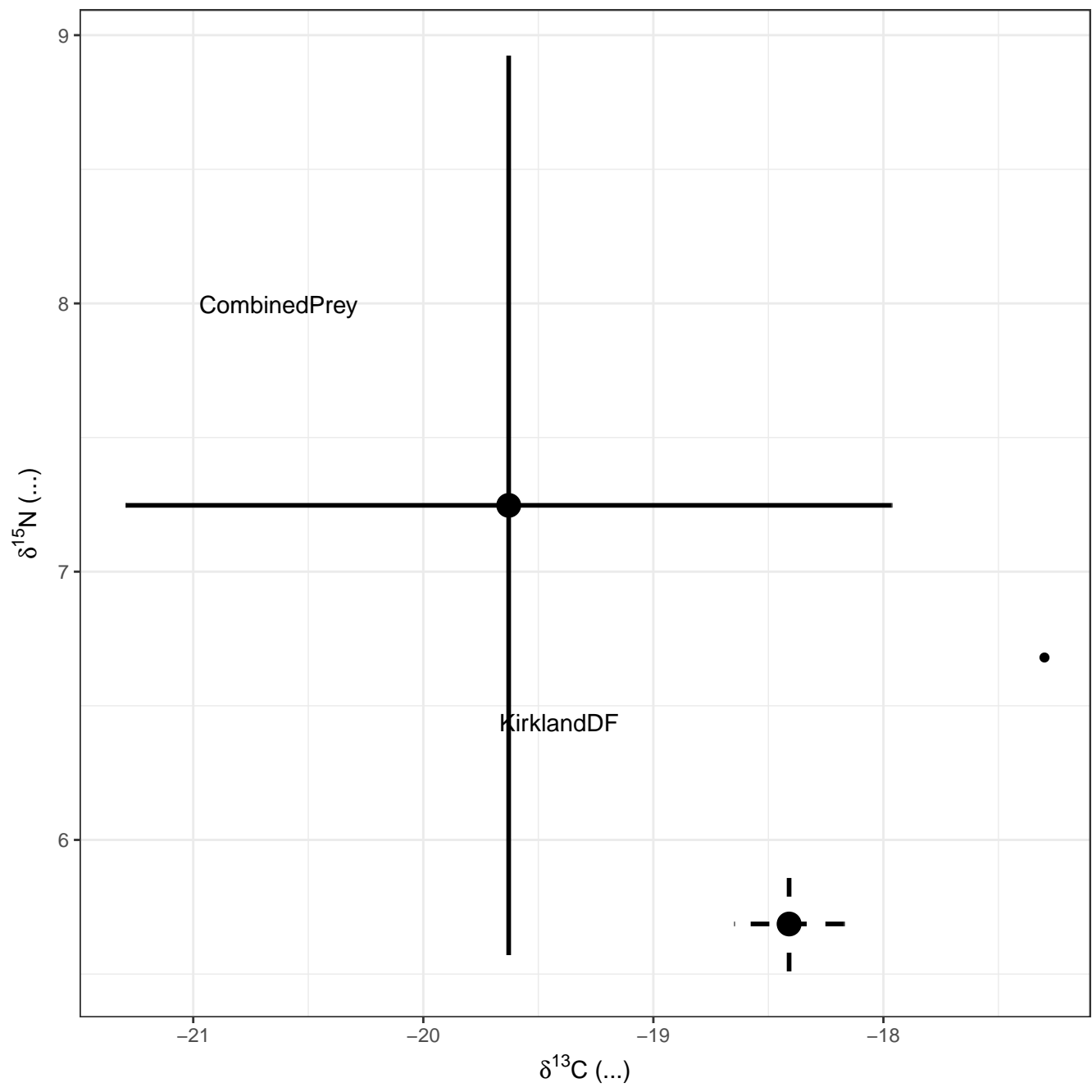

Supplement: Supplemental Information 2 — Contains isospace plot generated in MixSIAR for each cat in analysis. [file peerj-08-8337-s007.zip › Isospace plots/DFluffypants_isospace combadj.pdf]

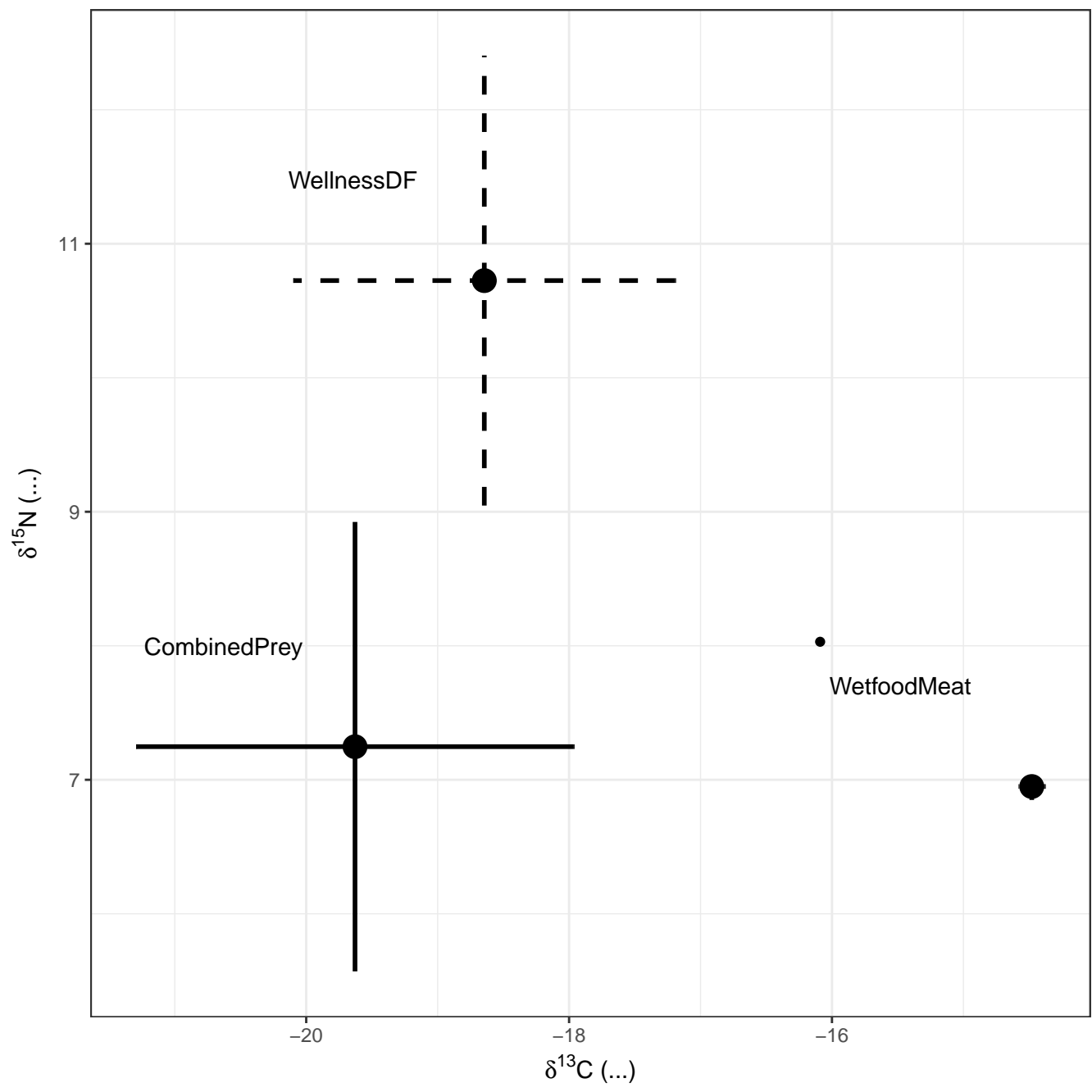

Supplement: Supplemental Information 2 — Contains isospace plot generated in MixSIAR for each cat in analysis. [file peerj-08-8337-s007.zip › Isospace plots/Douglas_isospace combadj.pdf]

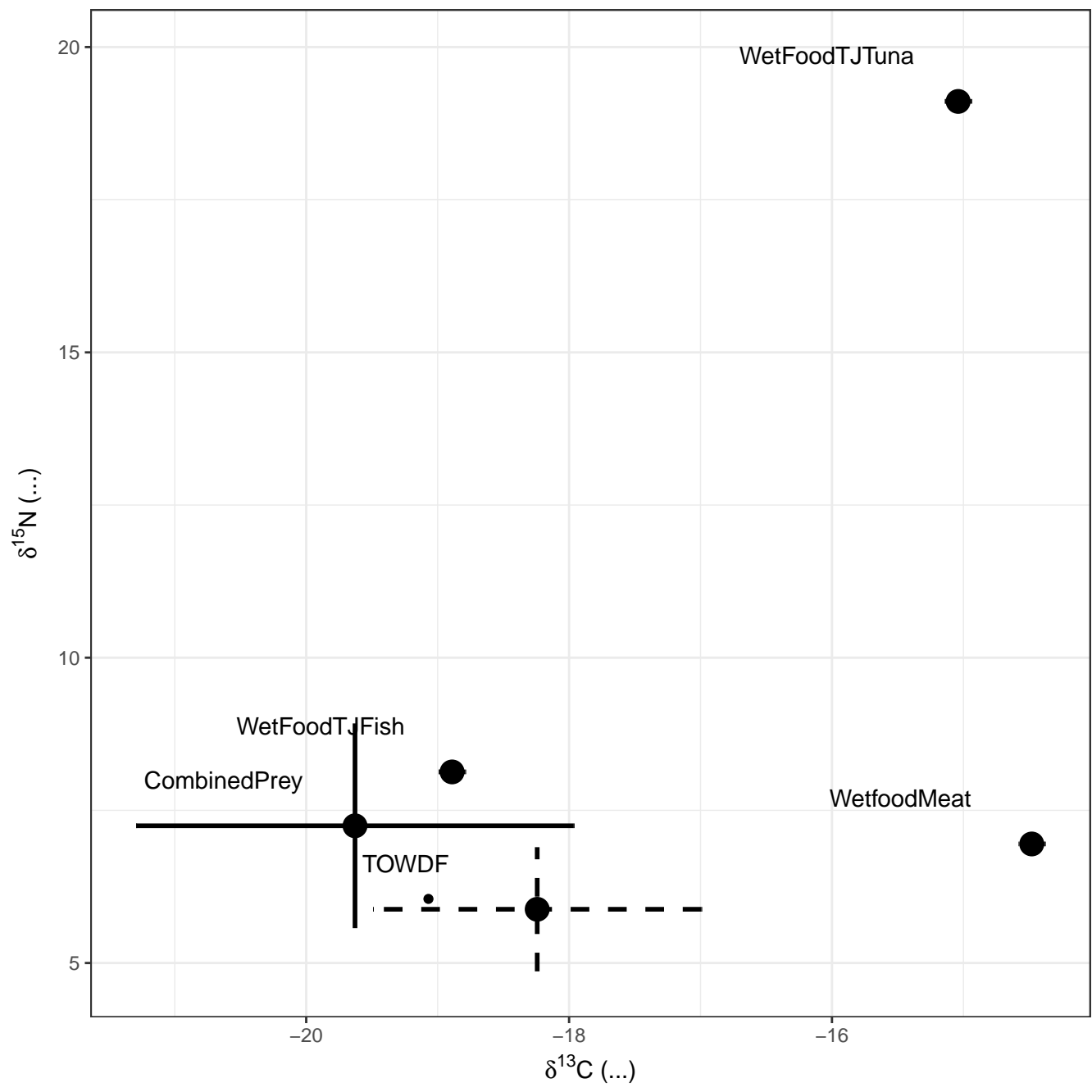

Supplement: Supplemental Information 2 — Contains isospace plot generated in MixSIAR for each cat in analysis. [file peerj-08-8337-s007.zip › Isospace plots/Ellie_isospace combadj.pdf]

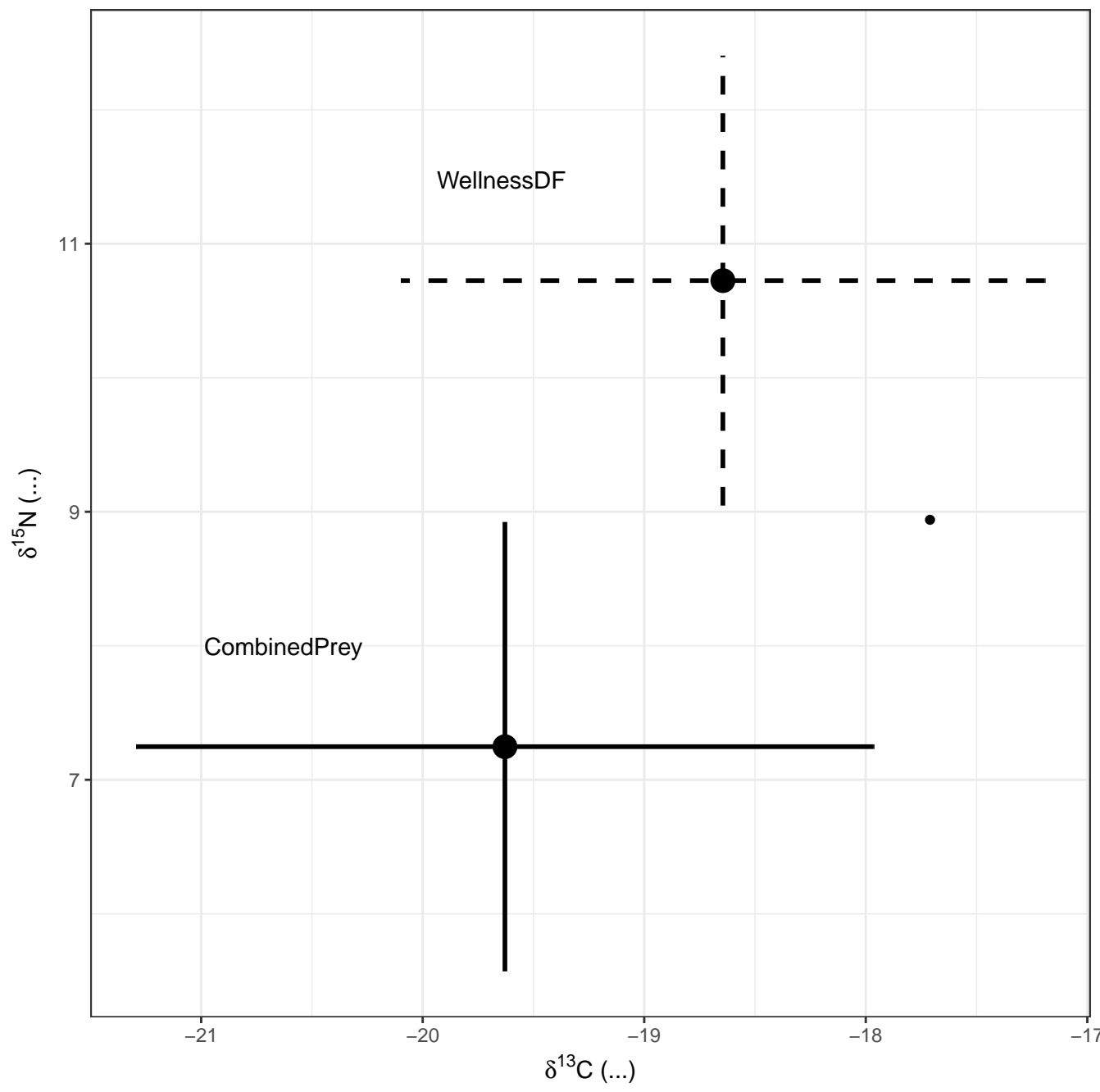

Supplement: Supplemental Information 2 — Contains isospace plot generated in MixSIAR for each cat in analysis. [file peerj-08-8337-s007.zip › Isospace plots/Fey_isospace combadj.pdf]

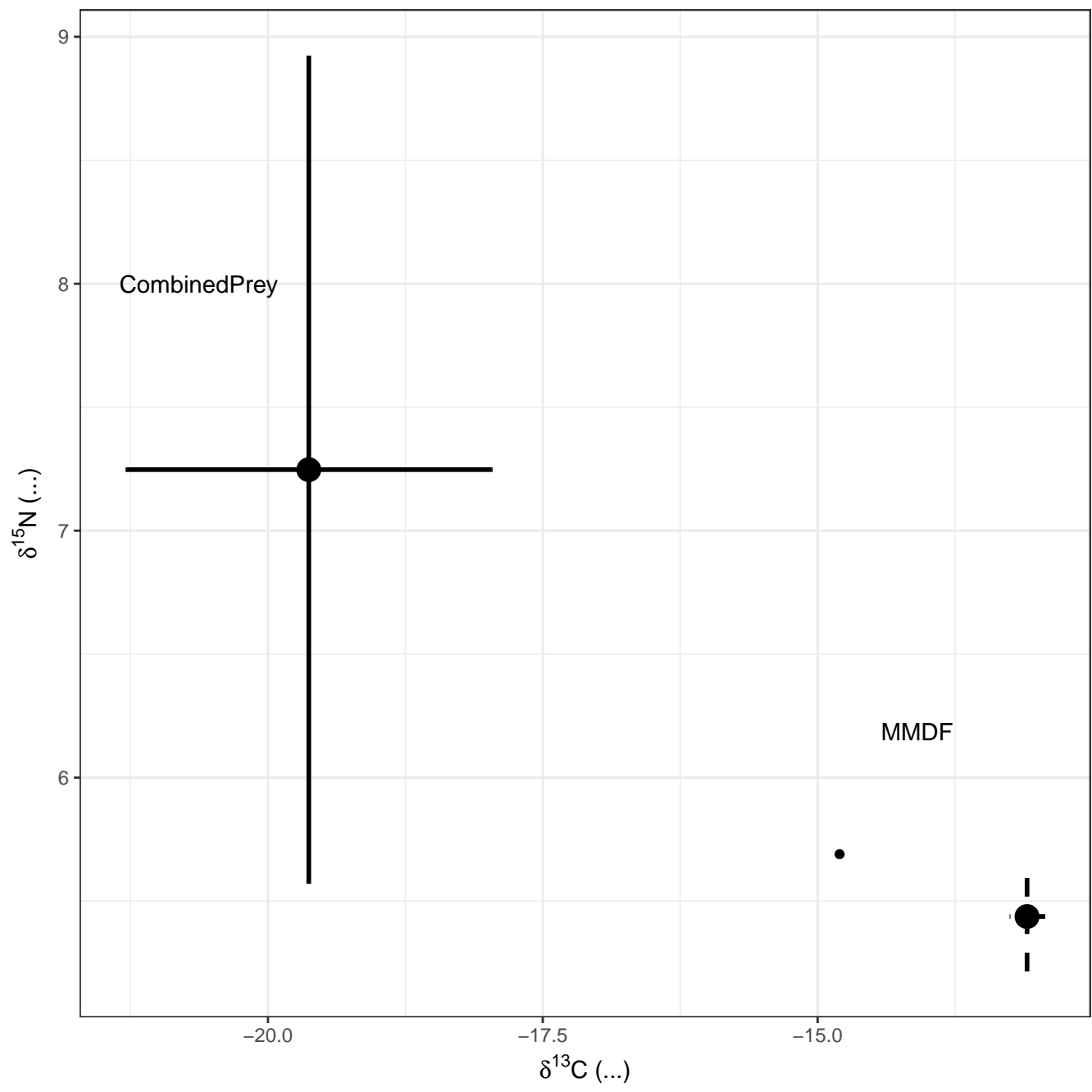

Supplement: Supplemental Information 2 — Contains isospace plot generated in MixSIAR for each cat in analysis. [file peerj-08-8337-s007.zip › Isospace plots/Fiona_isospace combadj.pdf]

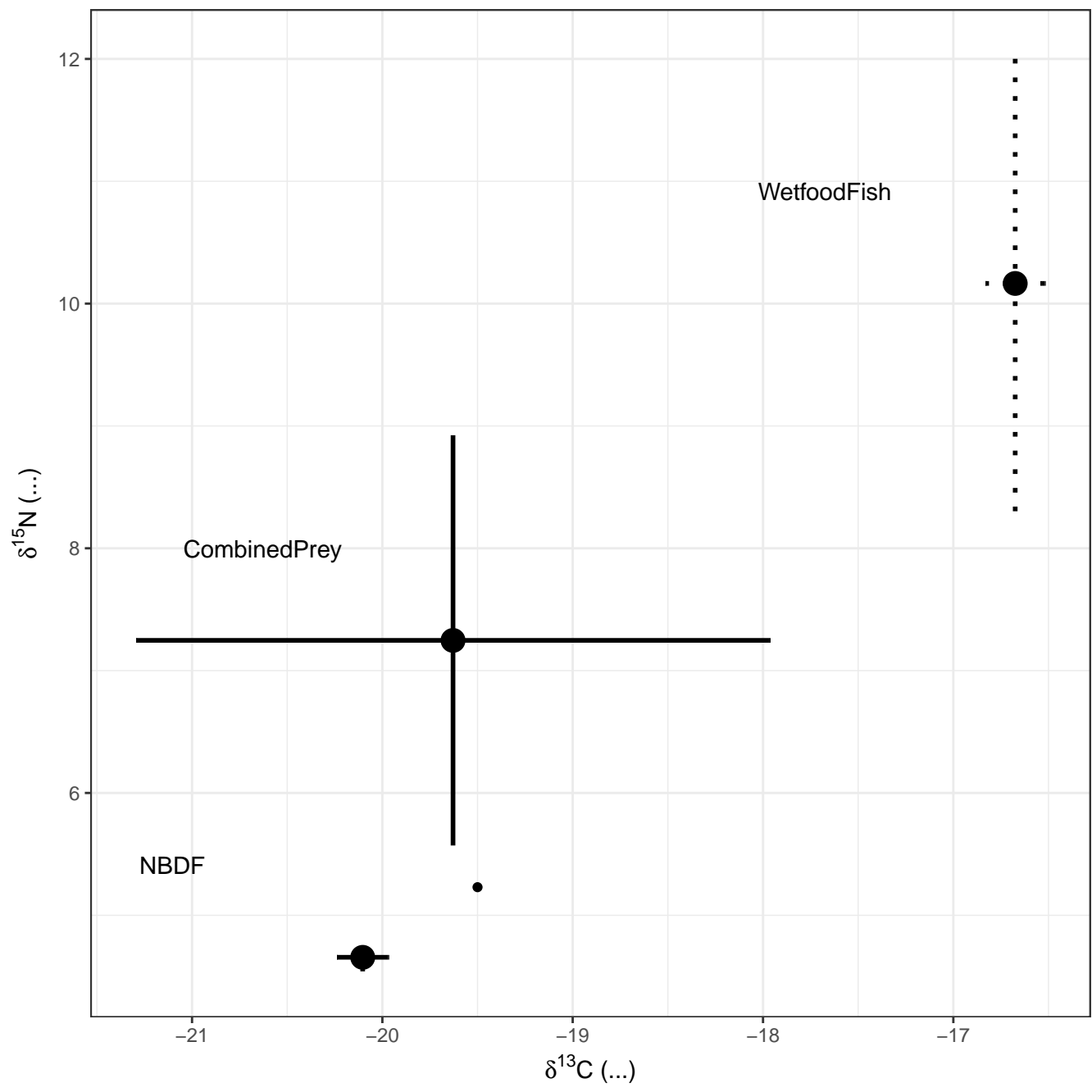

Supplement: Supplemental Information 2 — Contains isospace plot generated in MixSIAR for each cat in analysis. [file peerj-08-8337-s007.zip › Isospace plots/Gianni_isospace combadj.pdf]

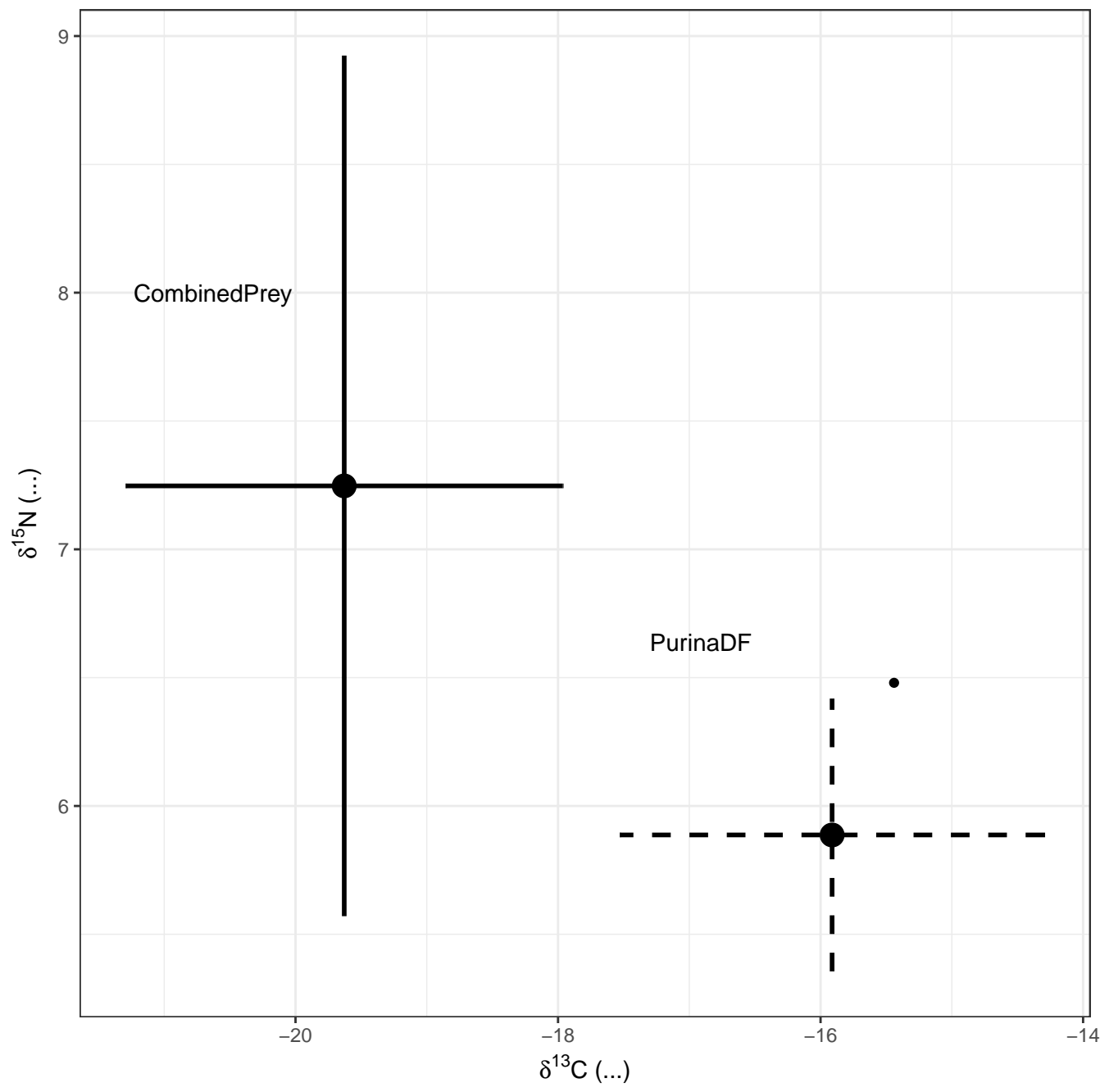

Supplement: Supplemental Information 2 — Contains isospace plot generated in MixSIAR for each cat in analysis. [file peerj-08-8337-s007.zip › Isospace plots/Gracie_isospace combadj.pdf]

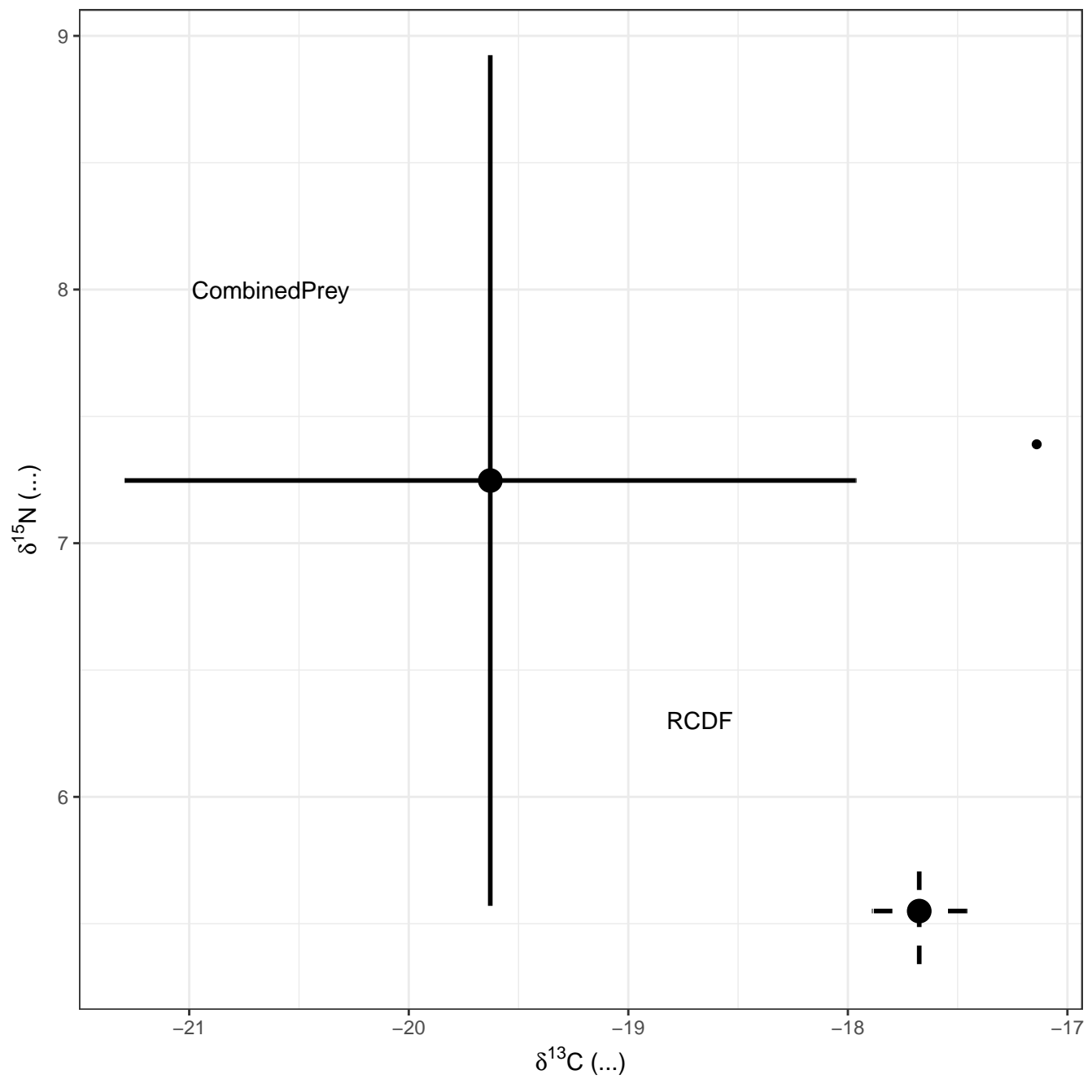

Supplement: Supplemental Information 2 — Contains isospace plot generated in MixSIAR for each cat in analysis. [file peerj-08-8337-s007.zip › Isospace plots/Hailey_isospace combadj.pdf]

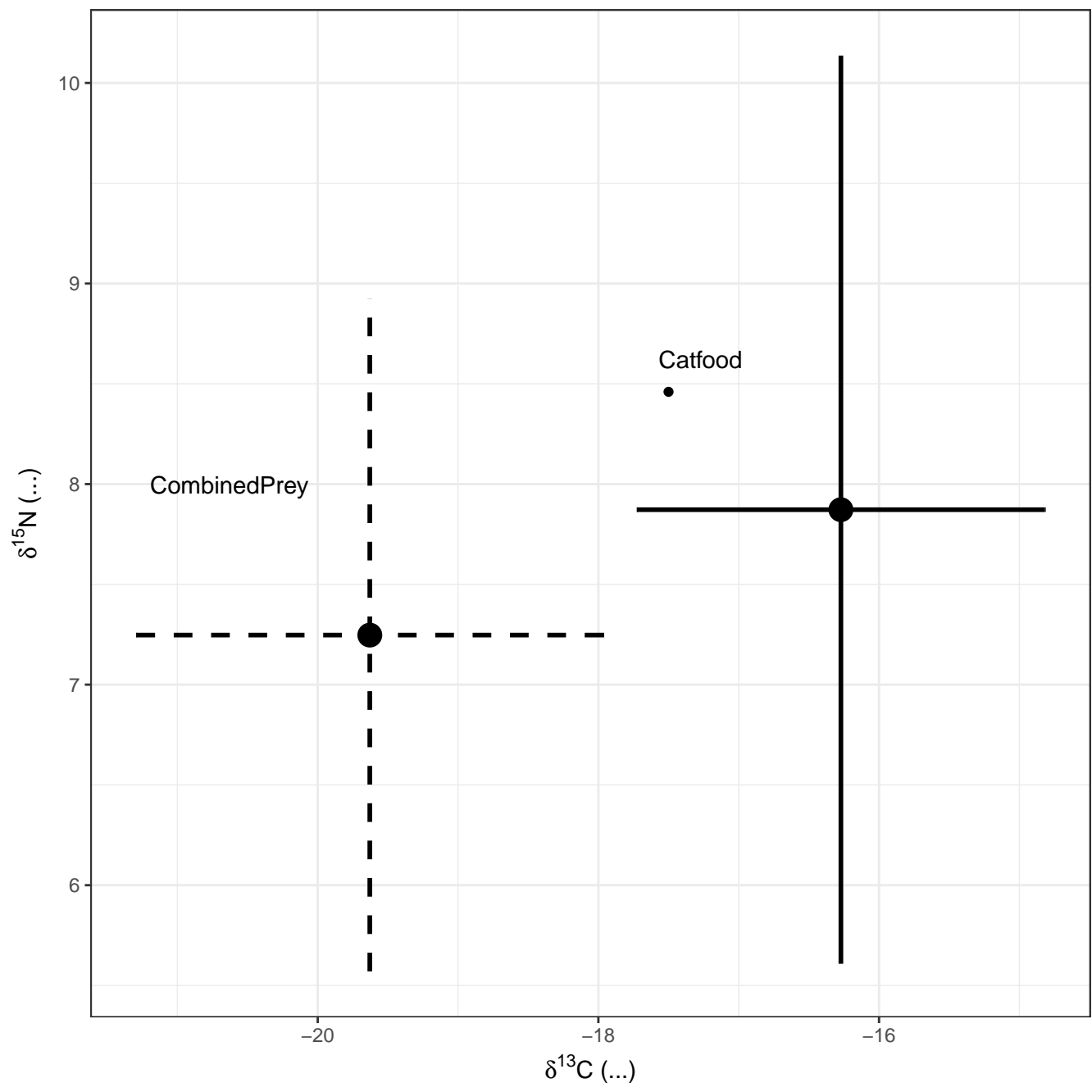

Supplement: Supplemental Information 2 — Contains isospace plot generated in MixSIAR for each cat in analysis. [file peerj-08-8337-s007.zip › Isospace plots/HazelG_isospace combadj.pdf]

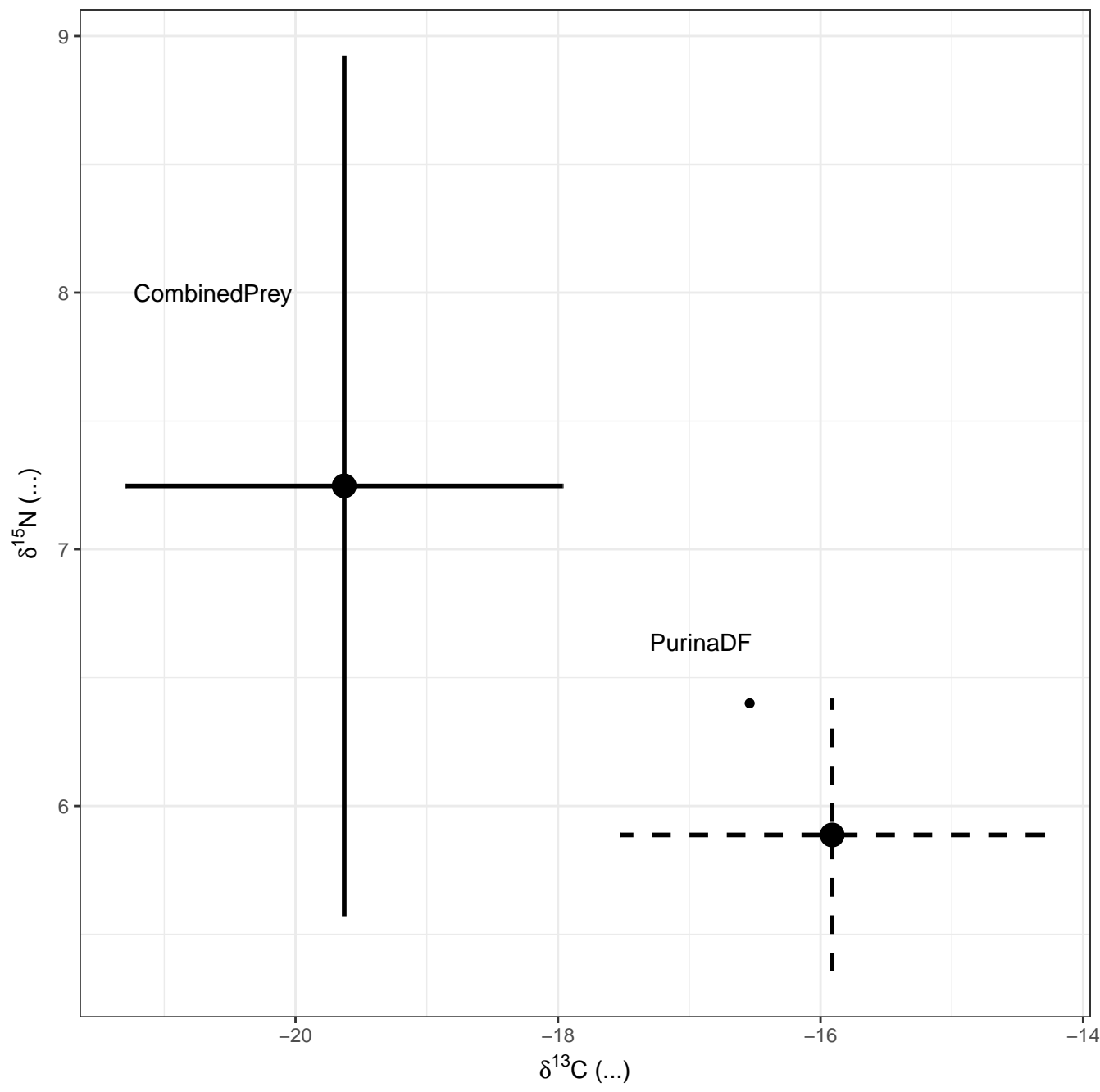

Supplement: Supplemental Information 2 — Contains isospace plot generated in MixSIAR for each cat in analysis. [file peerj-08-8337-s007.zip › Isospace plots/Hershey_isospace combadj.pdf]

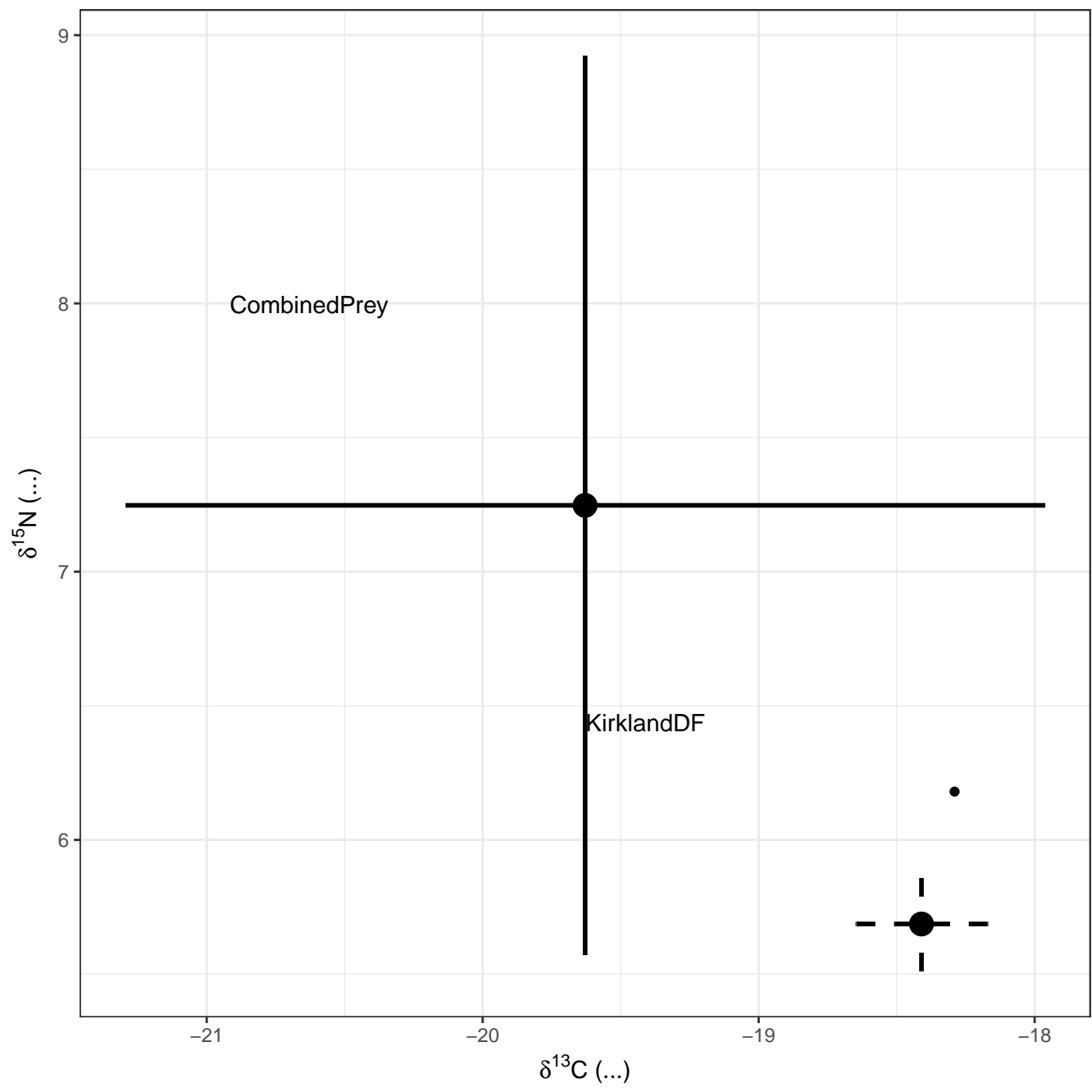

Supplement: Supplemental Information 2 — Contains isospace plot generated in MixSIAR for each cat in analysis. [file peerj-08-8337-s007.zip › Isospace plots/Jetpack_isosapce combadj.pdf]

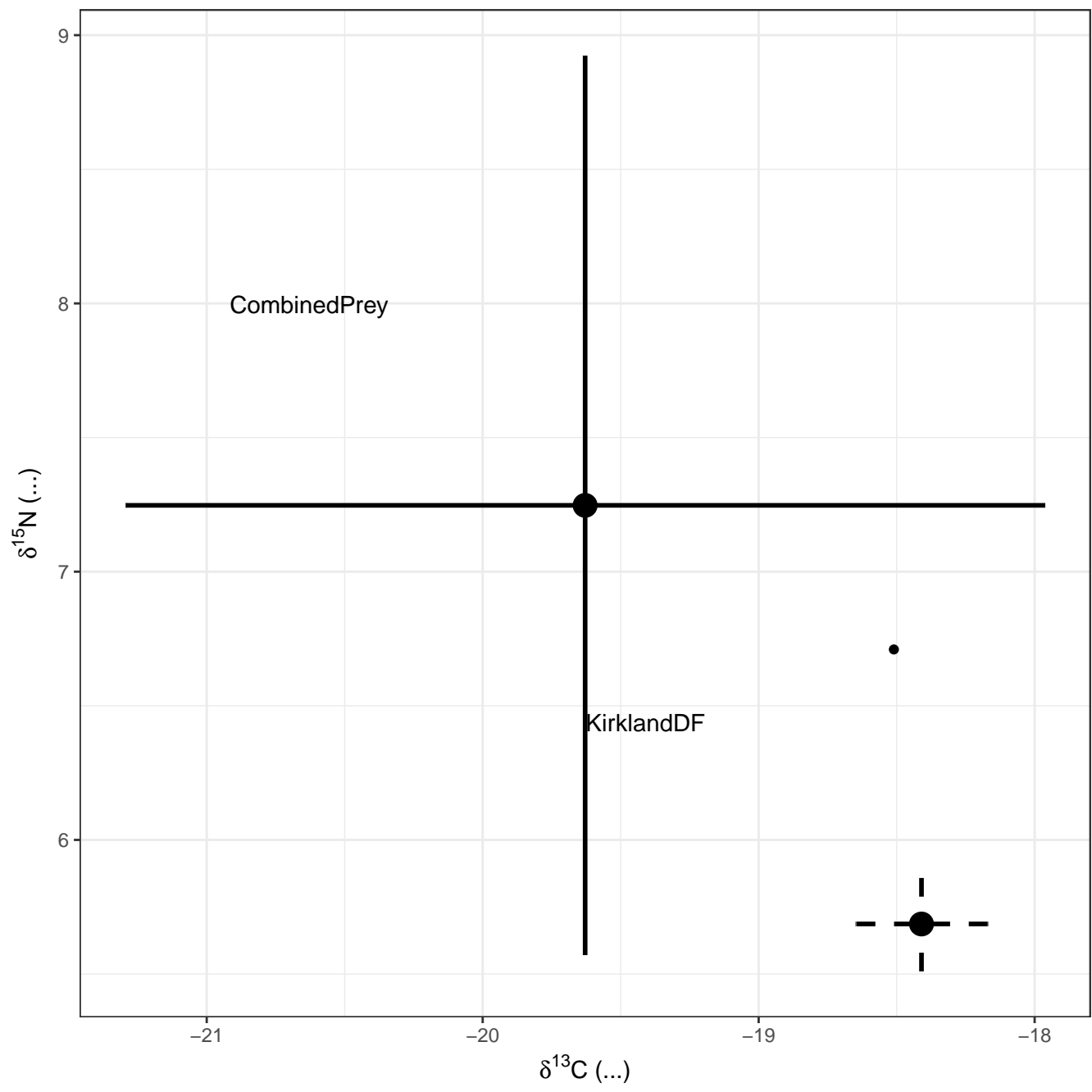

Supplement: Supplemental Information 2 — Contains isospace plot generated in MixSIAR for each cat in analysis. [file peerj-08-8337-s007.zip › Isospace plots/Jitterbug_isospace combadj.pdf]

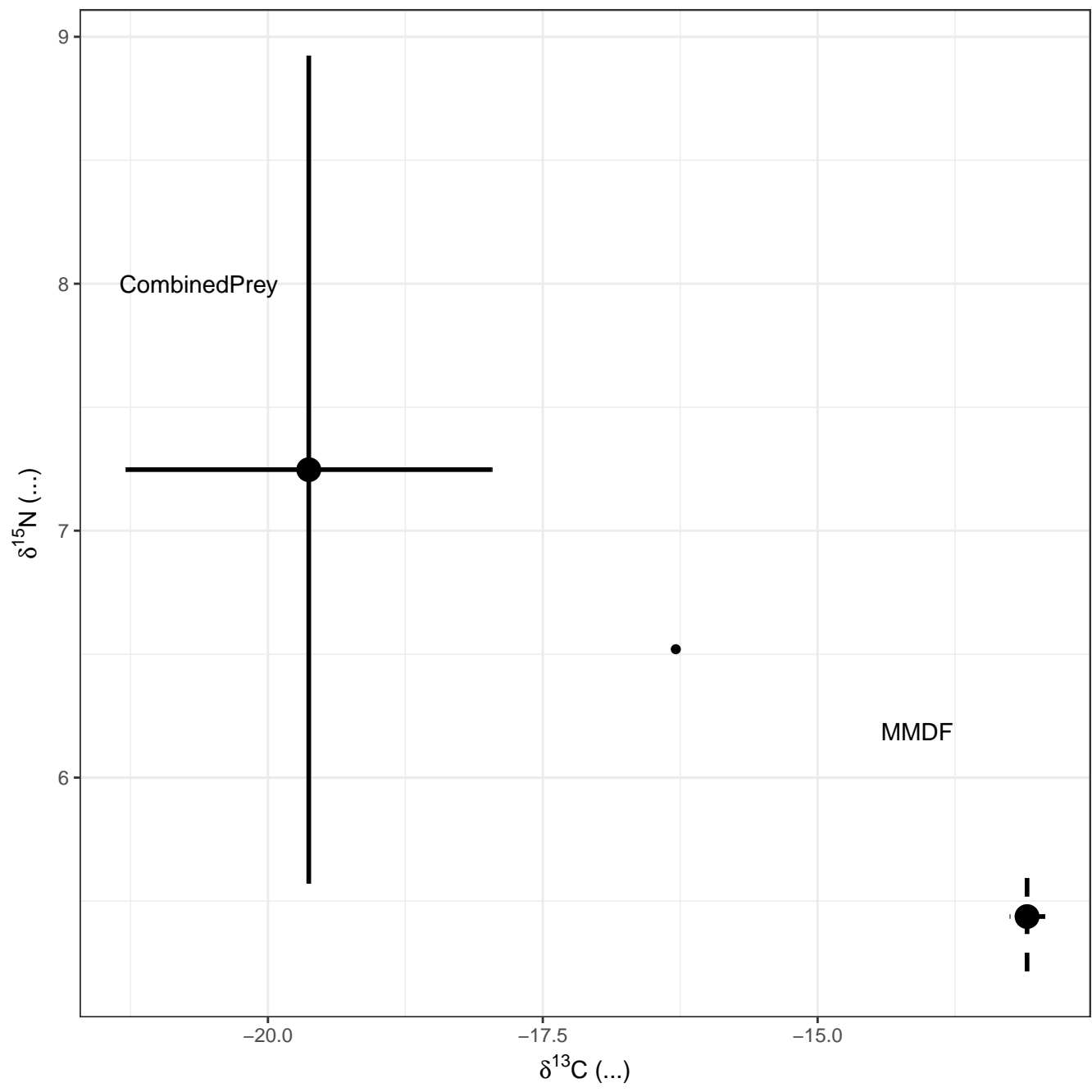

Supplement: Supplemental Information 2 — Contains isospace plot generated in MixSIAR for each cat in analysis. [file peerj-08-8337-s007.zip › Isospace plots/Joey_isospace combadj.pdf]

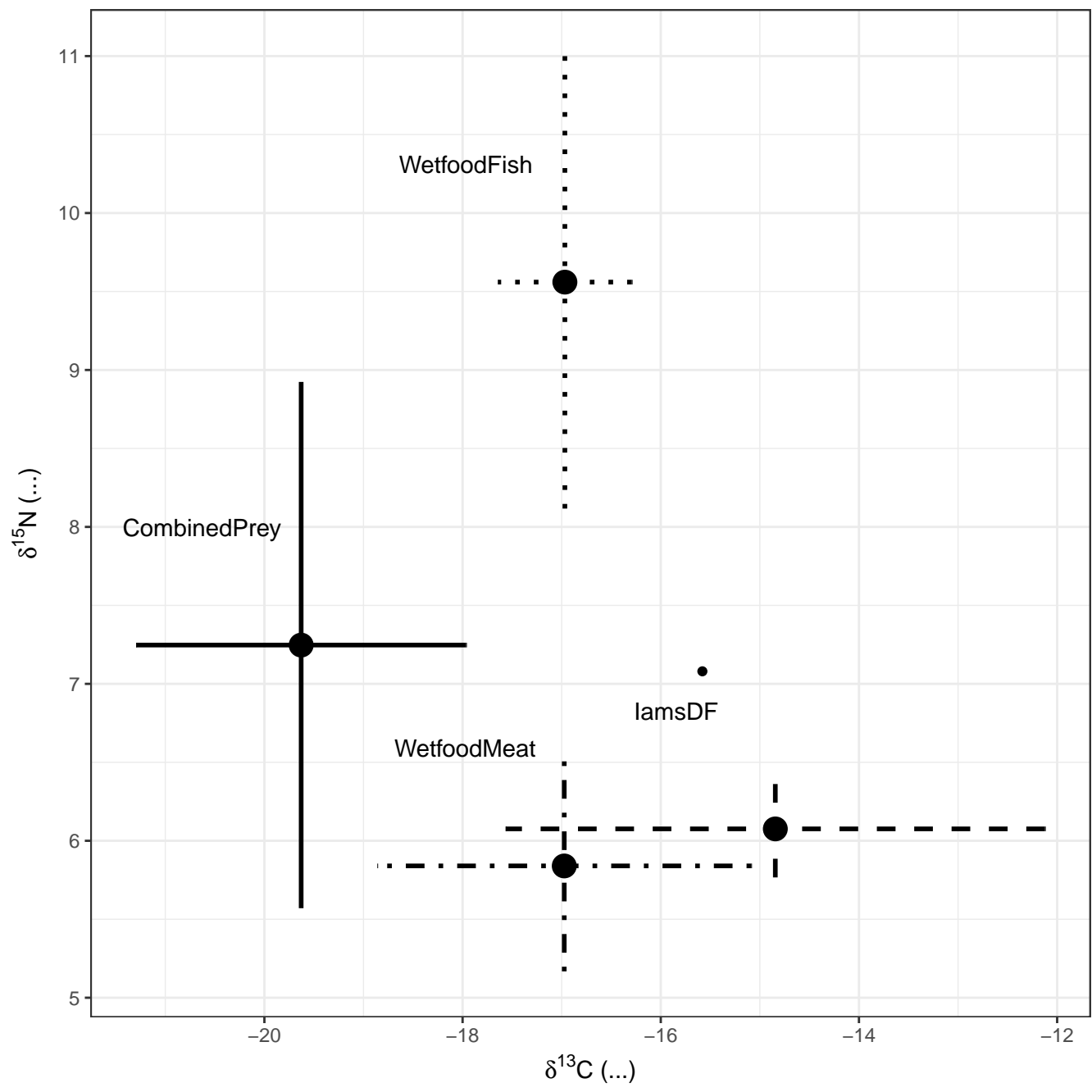

Supplement: Supplemental Information 2 — Contains isospace plot generated in MixSIAR for each cat in analysis. [file peerj-08-8337-s007.zip › Isospace plots/Kicky_isospace combadj.pdf]

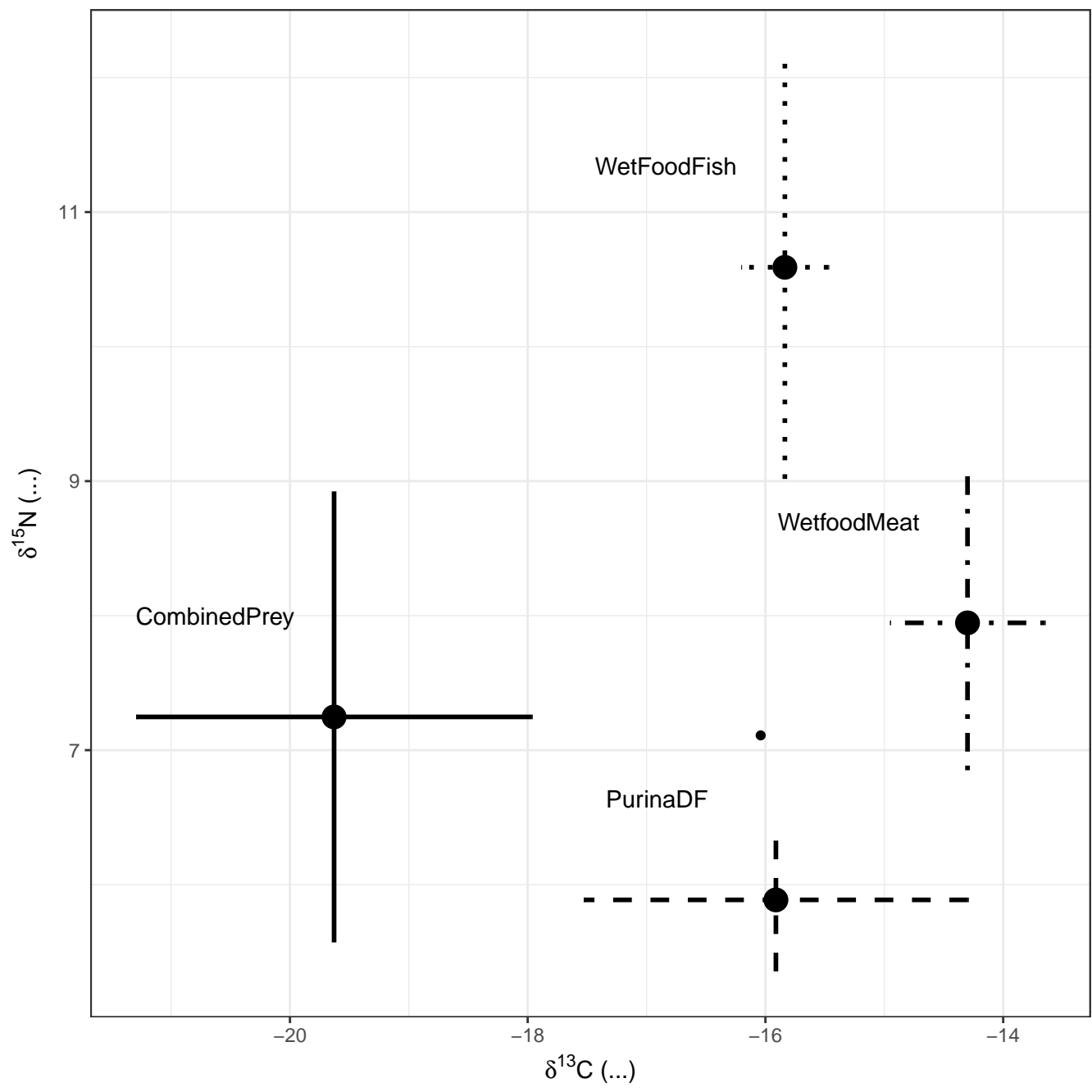

Supplement: Supplemental Information 2 — Contains isospace plot generated in MixSIAR for each cat in analysis. [file peerj-08-8337-s007.zip › Isospace plots/Leopard_isospace combadj.pdf]

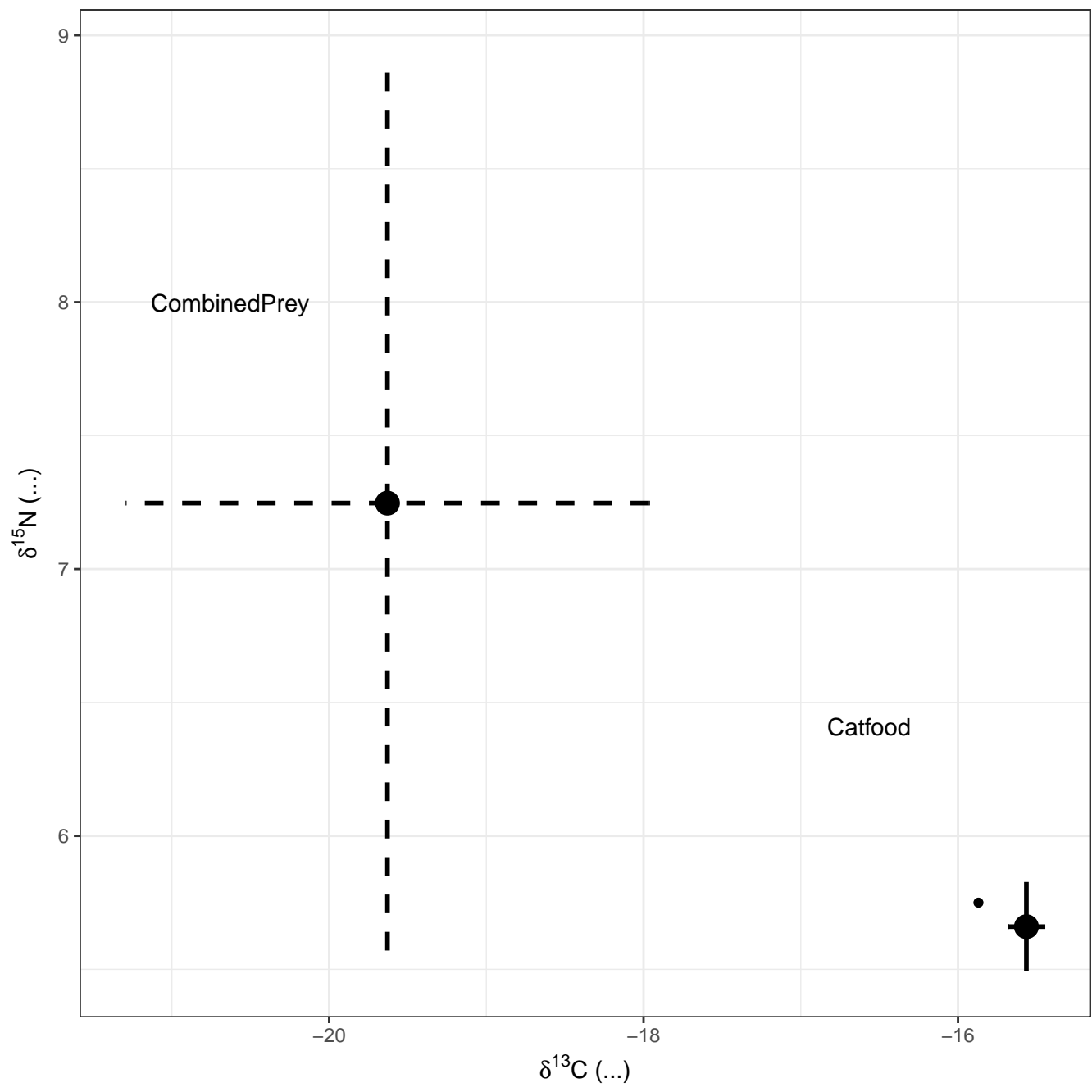

Supplement: Supplemental Information 2 — Contains isospace plot generated in MixSIAR for each cat in analysis. [file peerj-08-8337-s007.zip › Isospace plots/LunaA_isospace combadj.pdf]

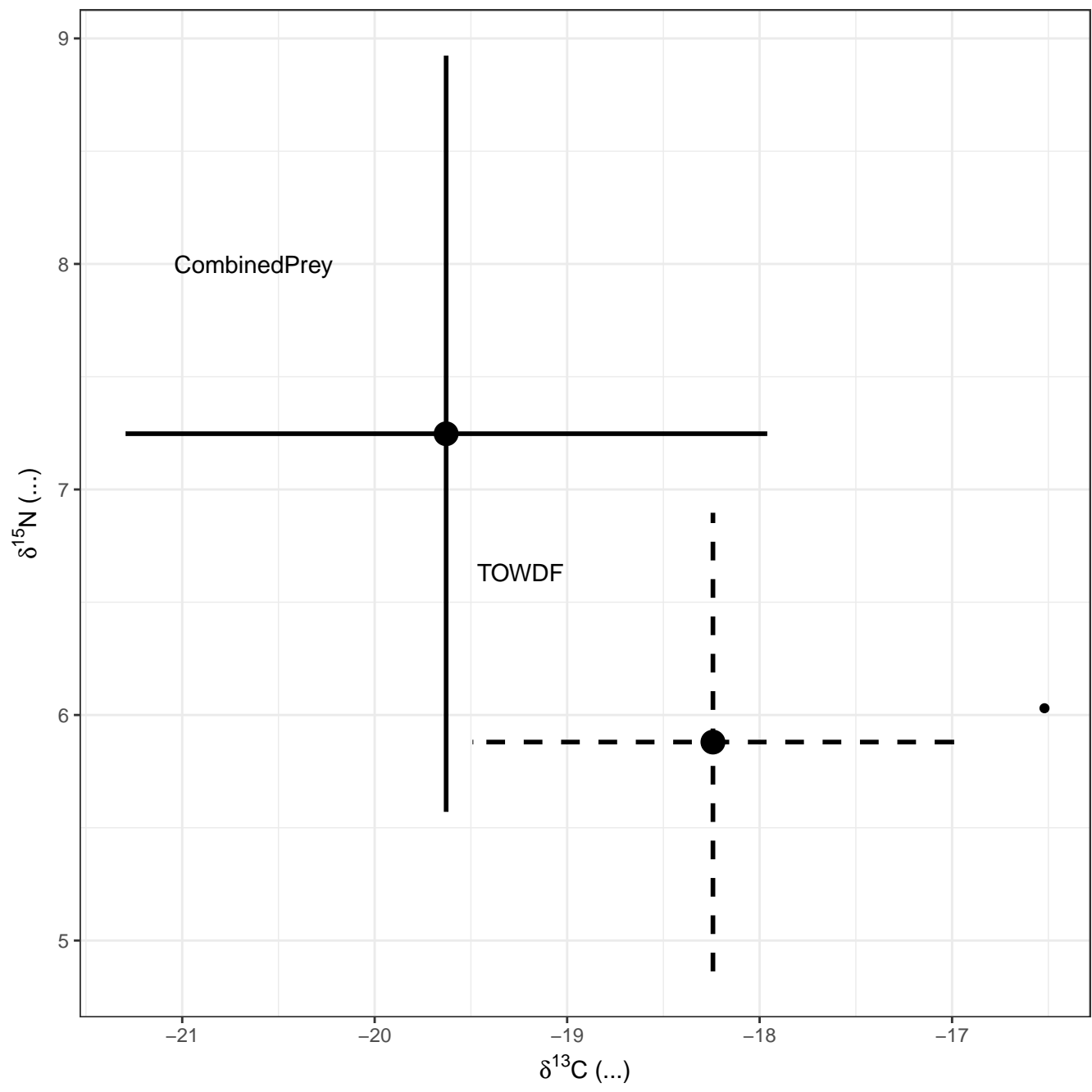

Supplement: Supplemental Information 2 — Contains isospace plot generated in MixSIAR for each cat in analysis. [file peerj-08-8337-s007.zip › Isospace plots/LunaM_isospaace combadj.pdf]

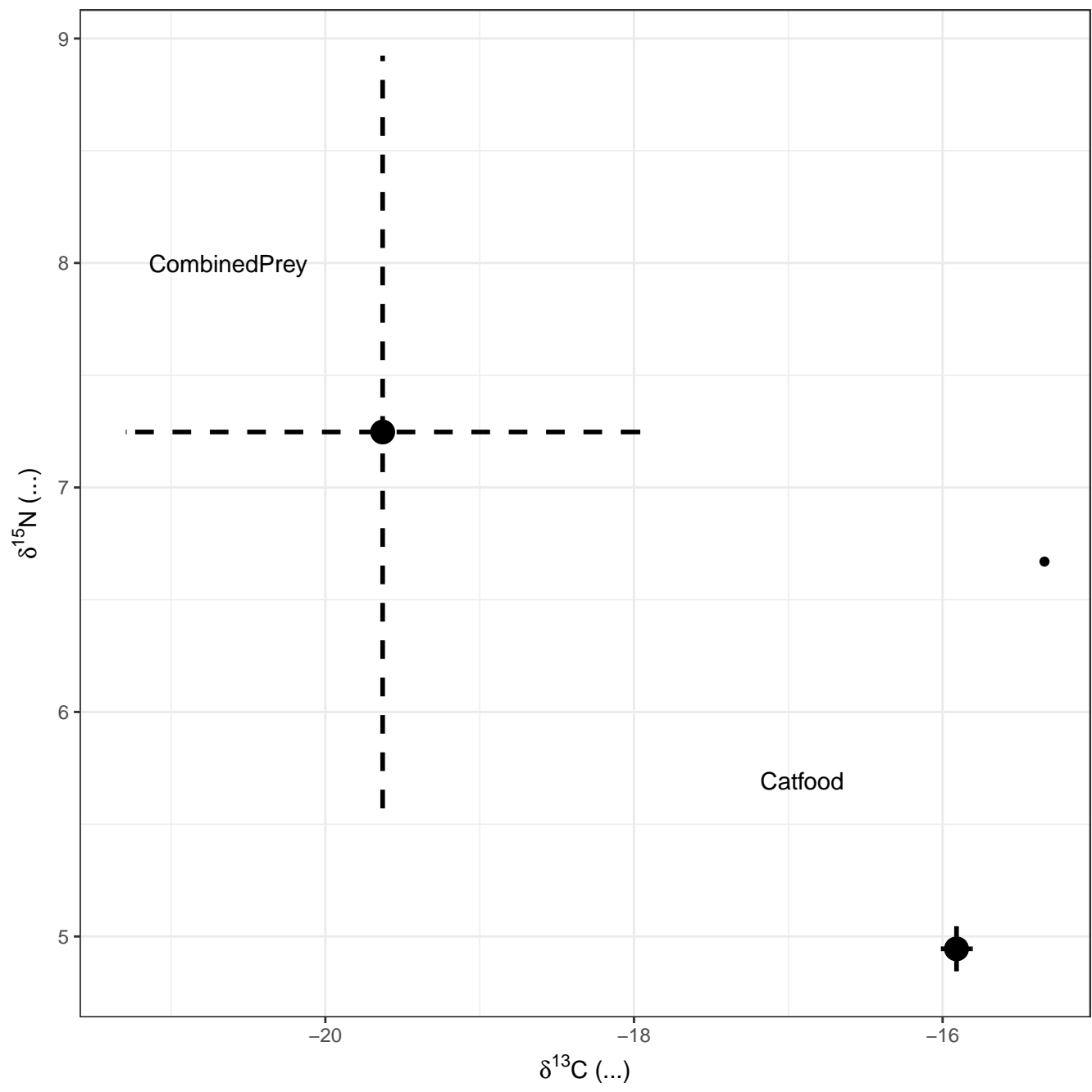

Supplement: Supplemental Information 2 — Contains isospace plot generated in MixSIAR for each cat in analysis. [file peerj-08-8337-s007.zip › Isospace plots/Nola_isospace combadj.pdf]

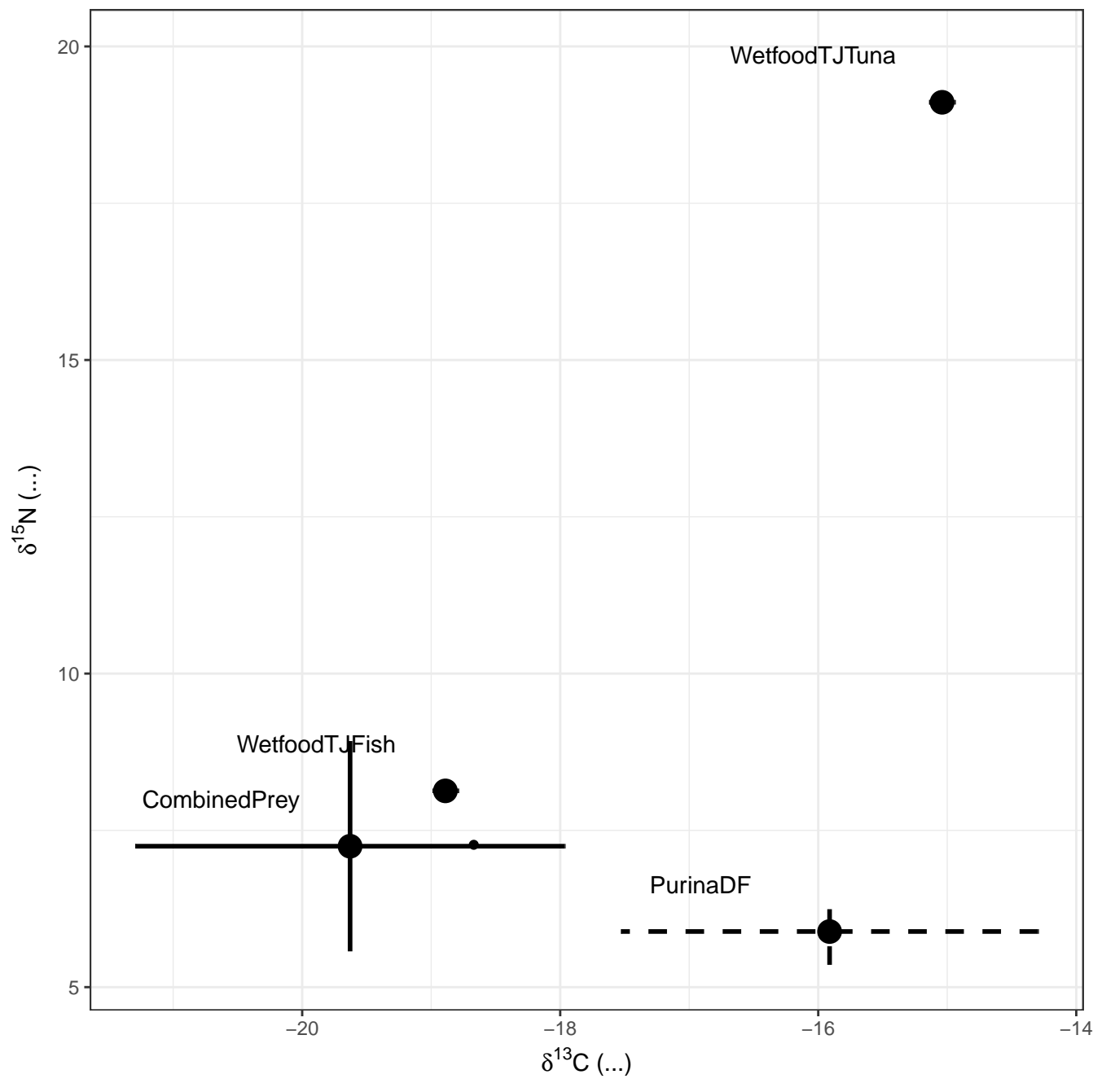

Supplement: Supplemental Information 2 — Contains isospace plot generated in MixSIAR for each cat in analysis. [file peerj-08-8337-s007.zip › Isospace plots/Oatmeal_isospace combadj.pdf]

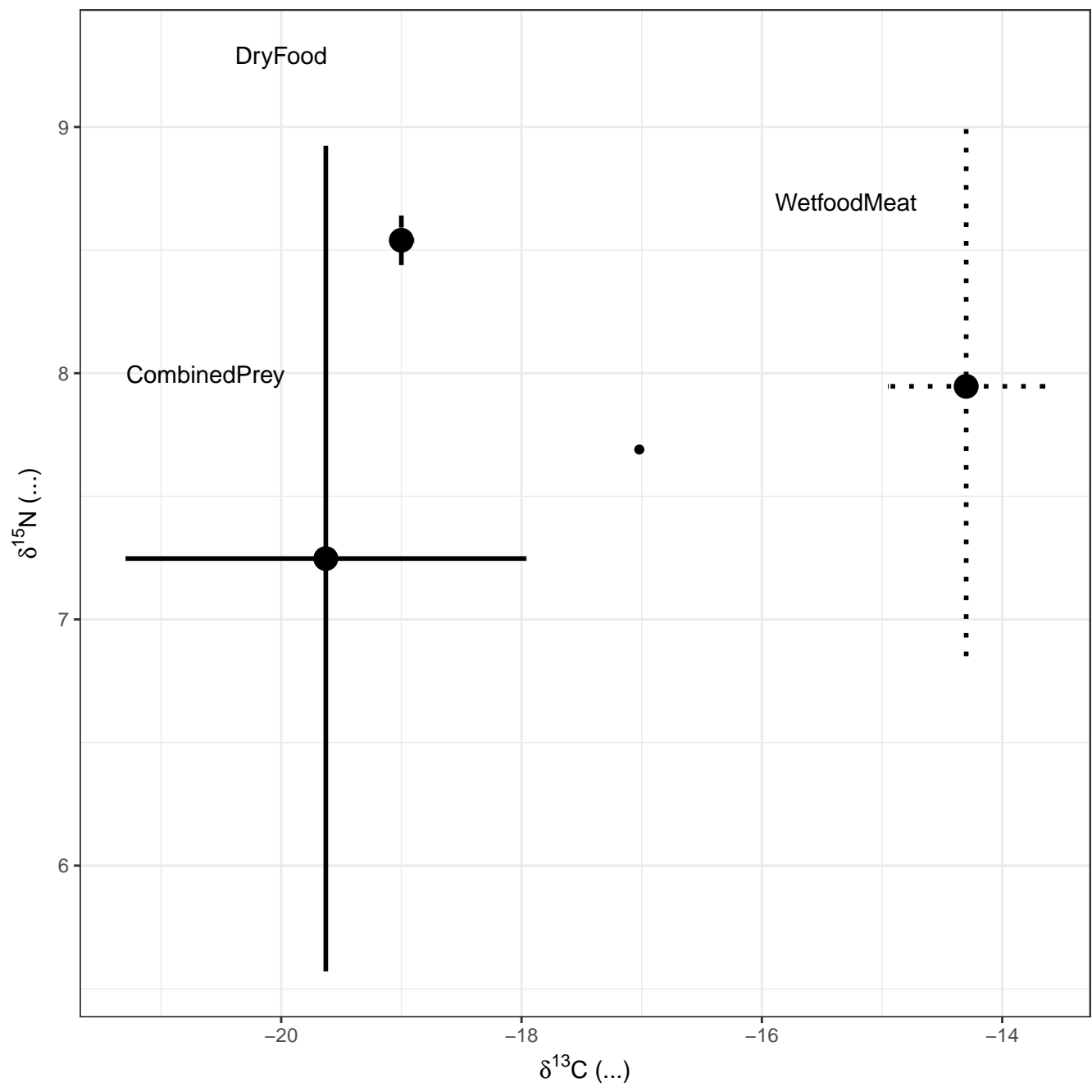

Supplement: Supplemental Information 2 — Contains isospace plot generated in MixSIAR for each cat in analysis. [file peerj-08-8337-s007.zip › Isospace plots/Orange_isospace combadj.pdf]

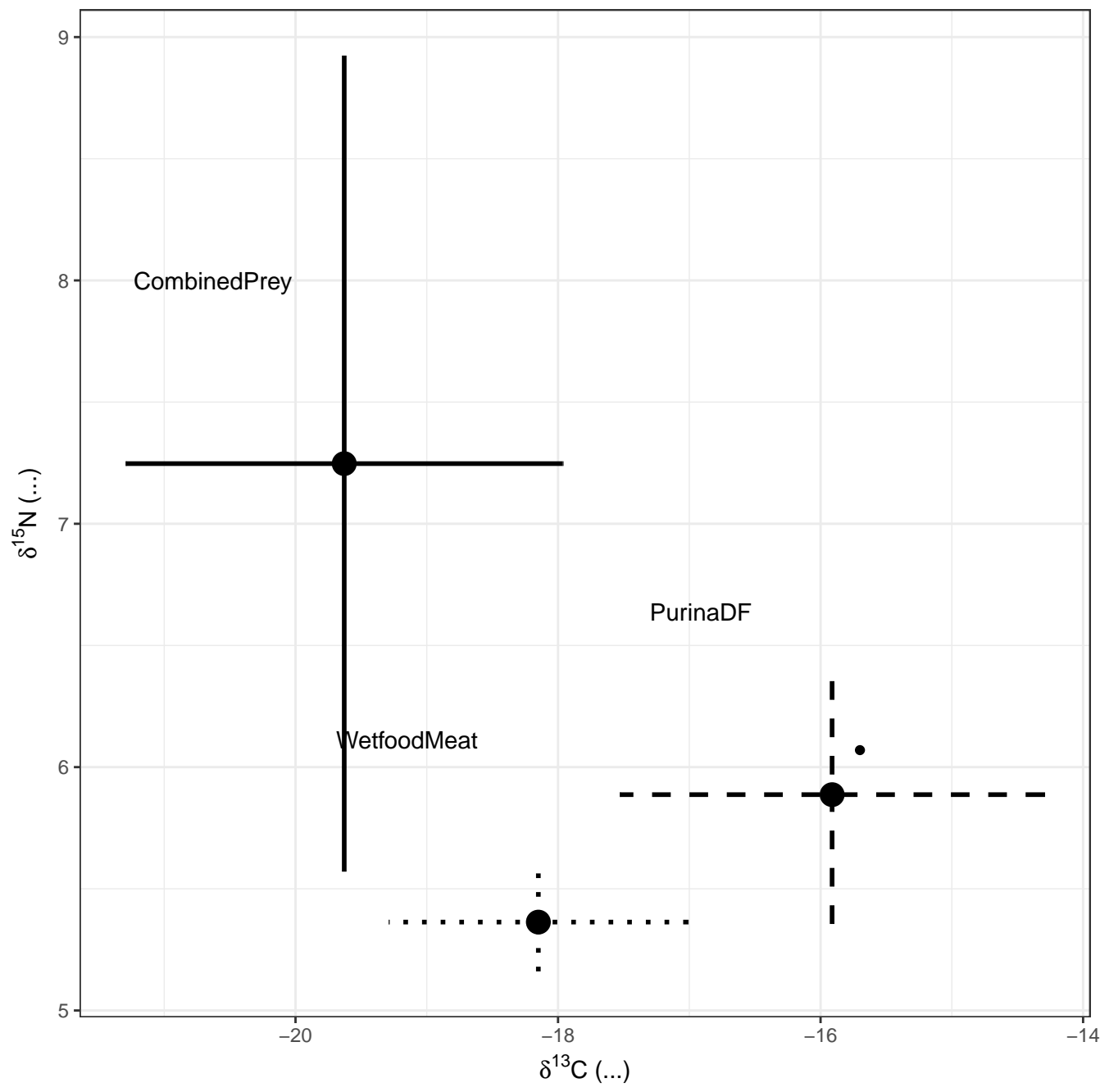

Supplement: Supplemental Information 2 — Contains isospace plot generated in MixSIAR for each cat in analysis. [file peerj-08-8337-s007.zip › Isospace plots/Oreo_isospace combadj.pdf]

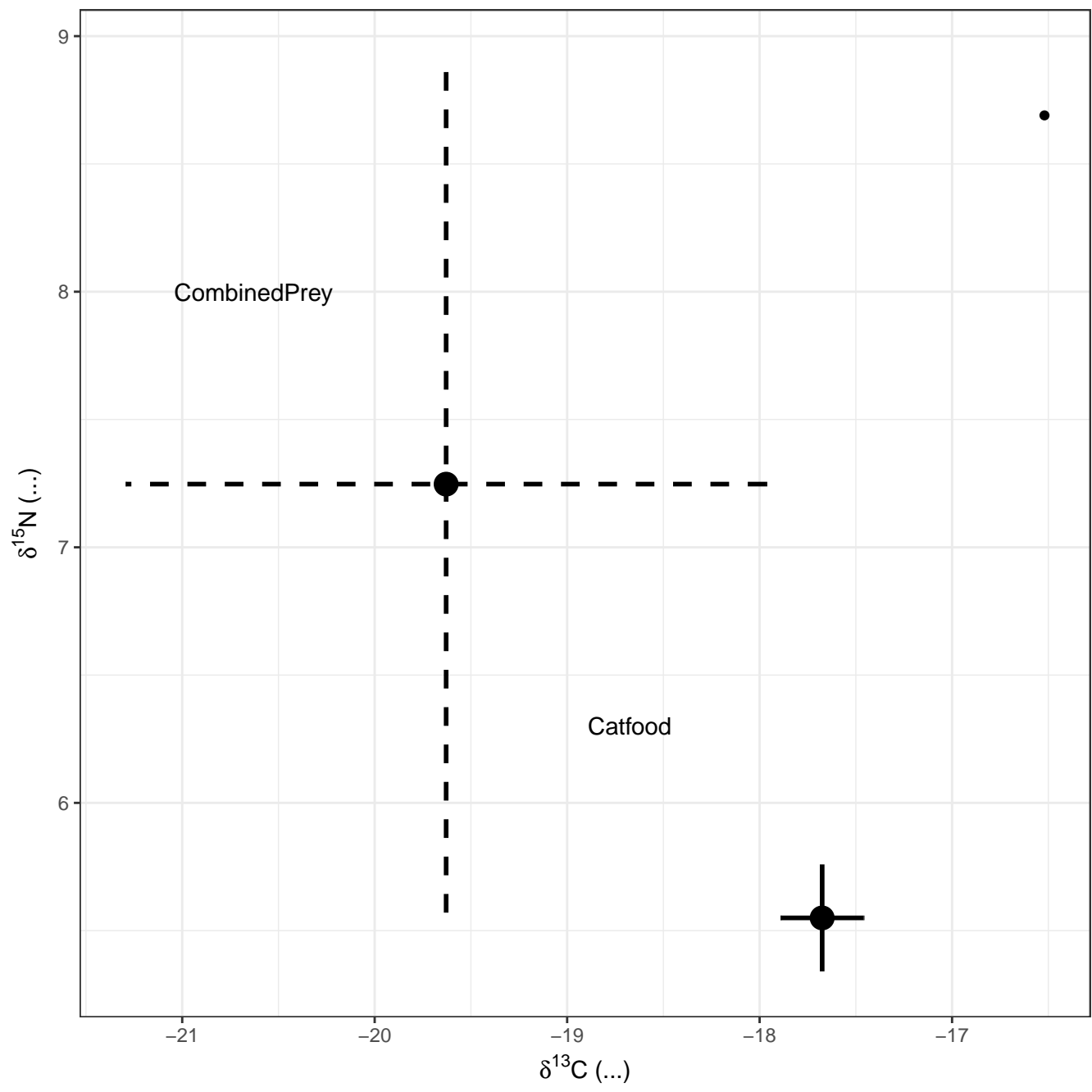

Supplement: Supplemental Information 2 — Contains isospace plot generated in MixSIAR for each cat in analysis. [file peerj-08-8337-s007.zip › Isospace plots/Pancho_isospace combadj.pdf]

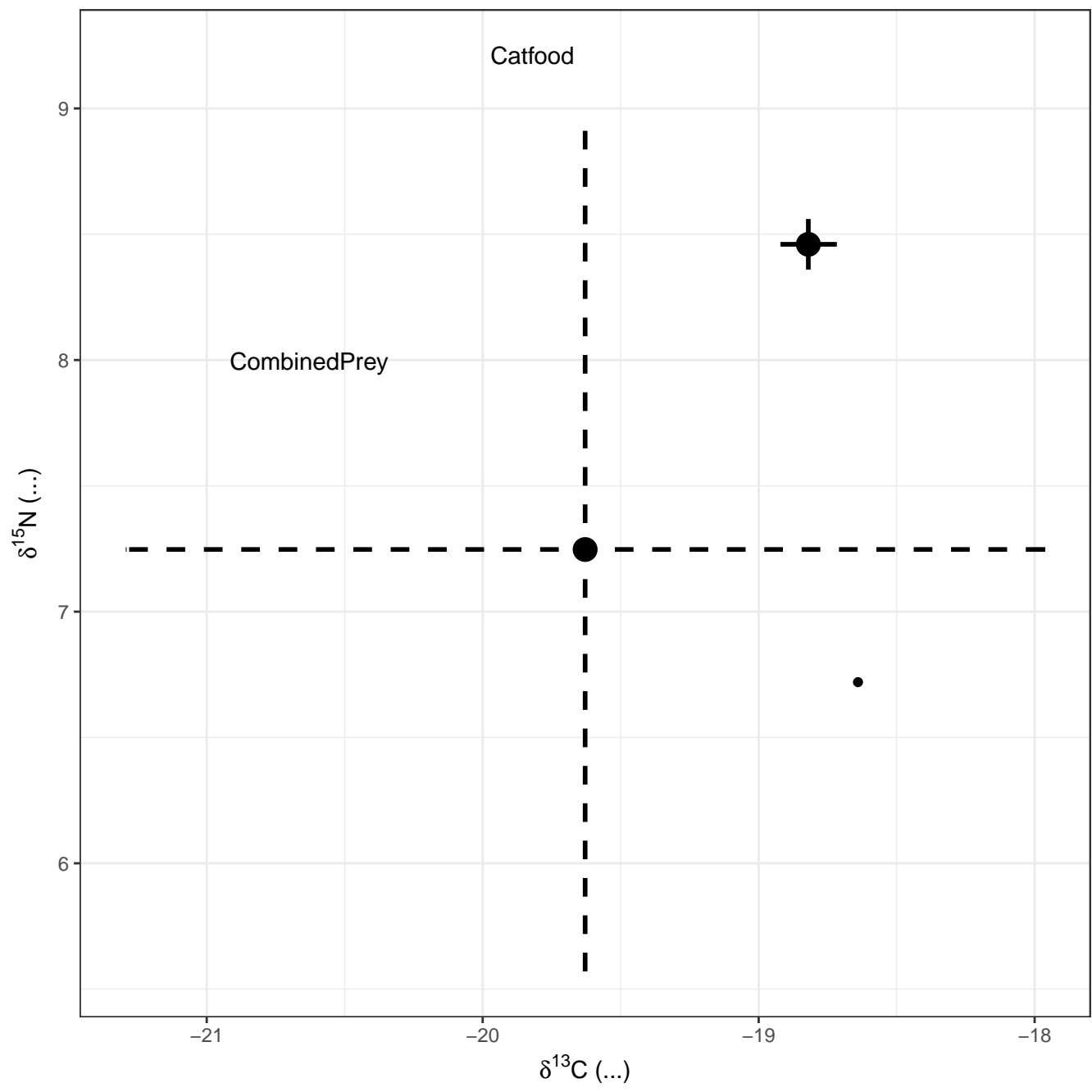

Supplement: Supplemental Information 2 — Contains isospace plot generated in MixSIAR for each cat in analysis. [file peerj-08-8337-s007.zip › Isospace plots/Pumpkin_isospace combadj.pdf]

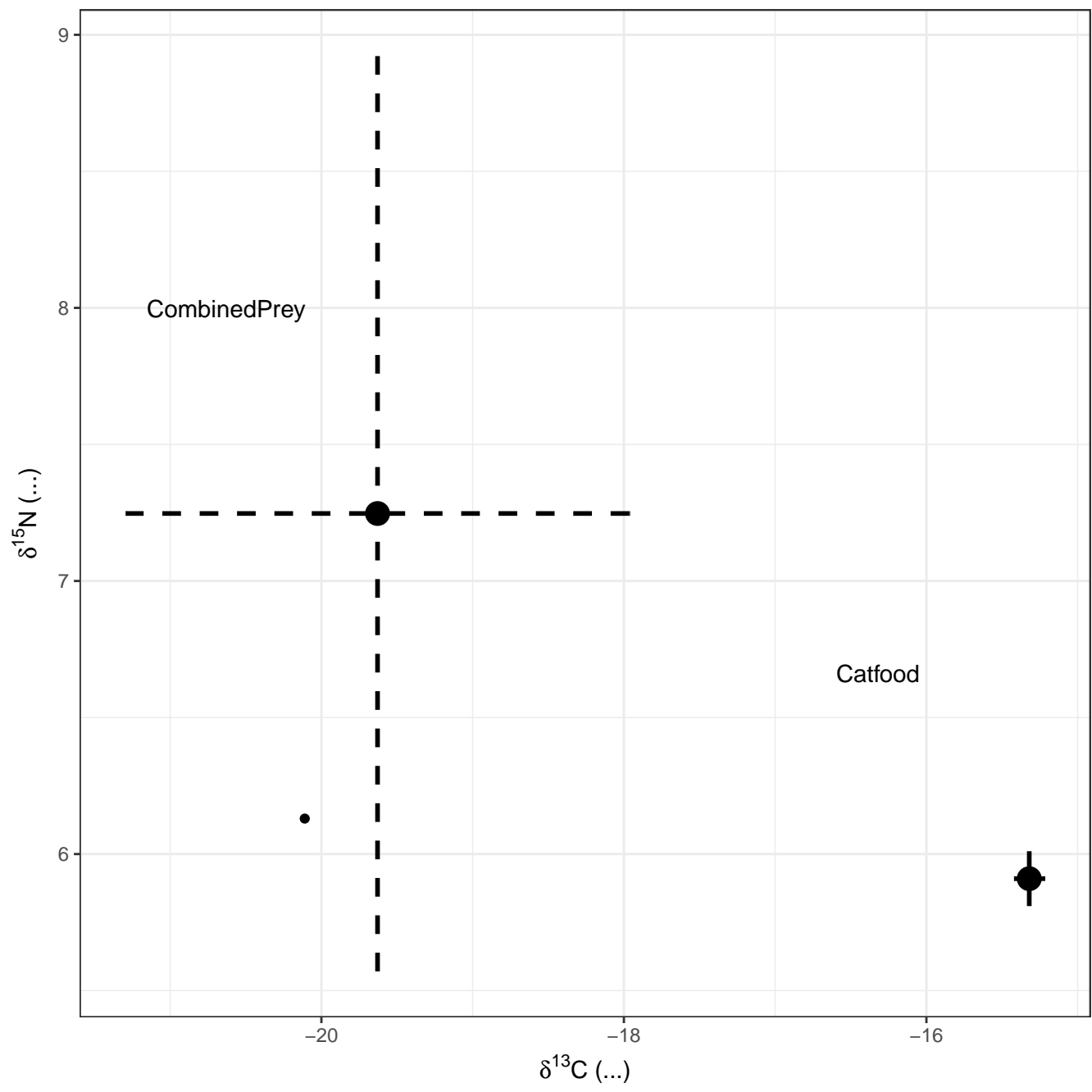

Supplement: Supplemental Information 2 — Contains isospace plot generated in MixSIAR for each cat in analysis. [file peerj-08-8337-s007.zip › Isospace plots/Sandstorm_isospace combadj.pdf]

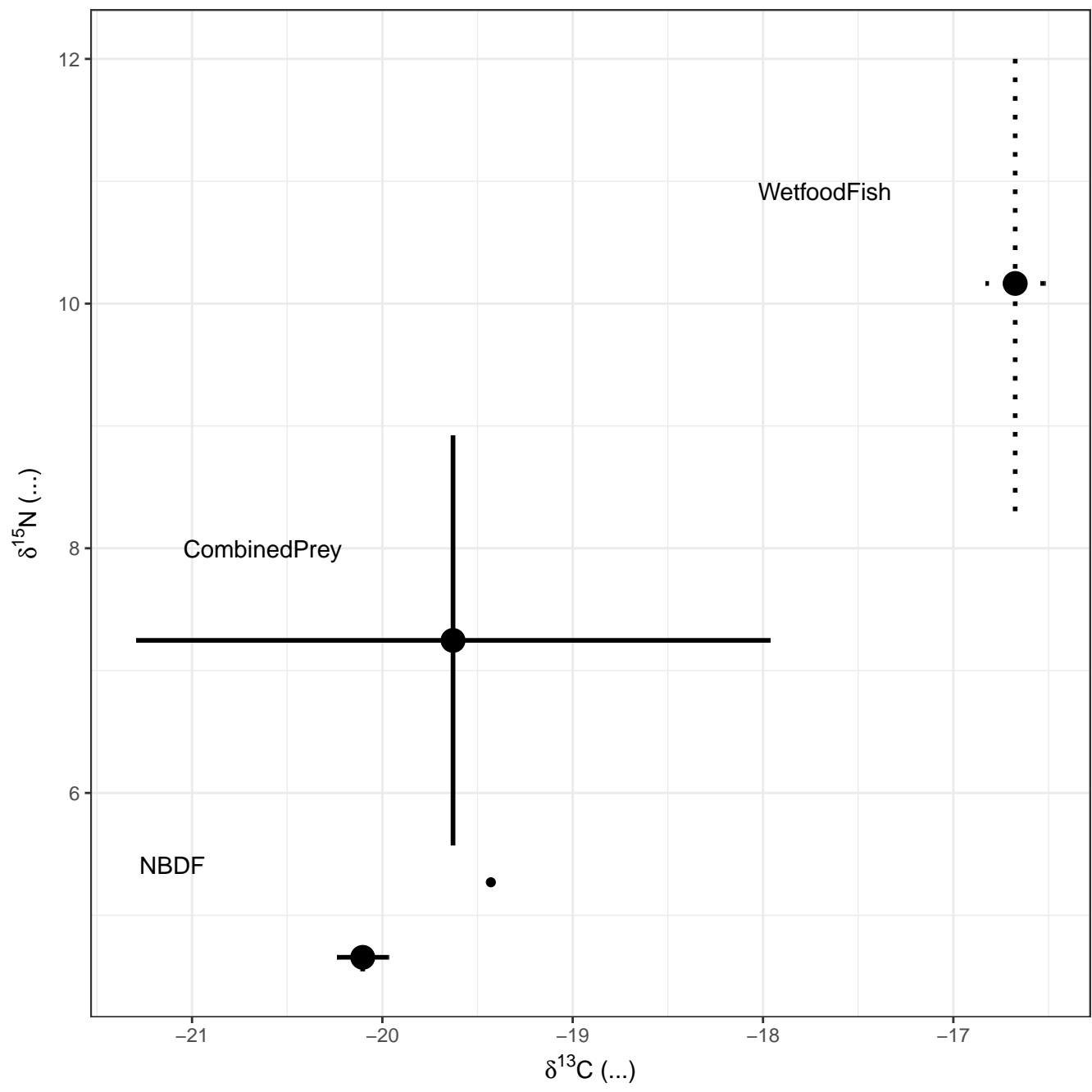

Supplement: Supplemental Information 2 — Contains isospace plot generated in MixSIAR for each cat in analysis. [file peerj-08-8337-s007.zip › Isospace plots/Selkie_isospace combadj.pdf]

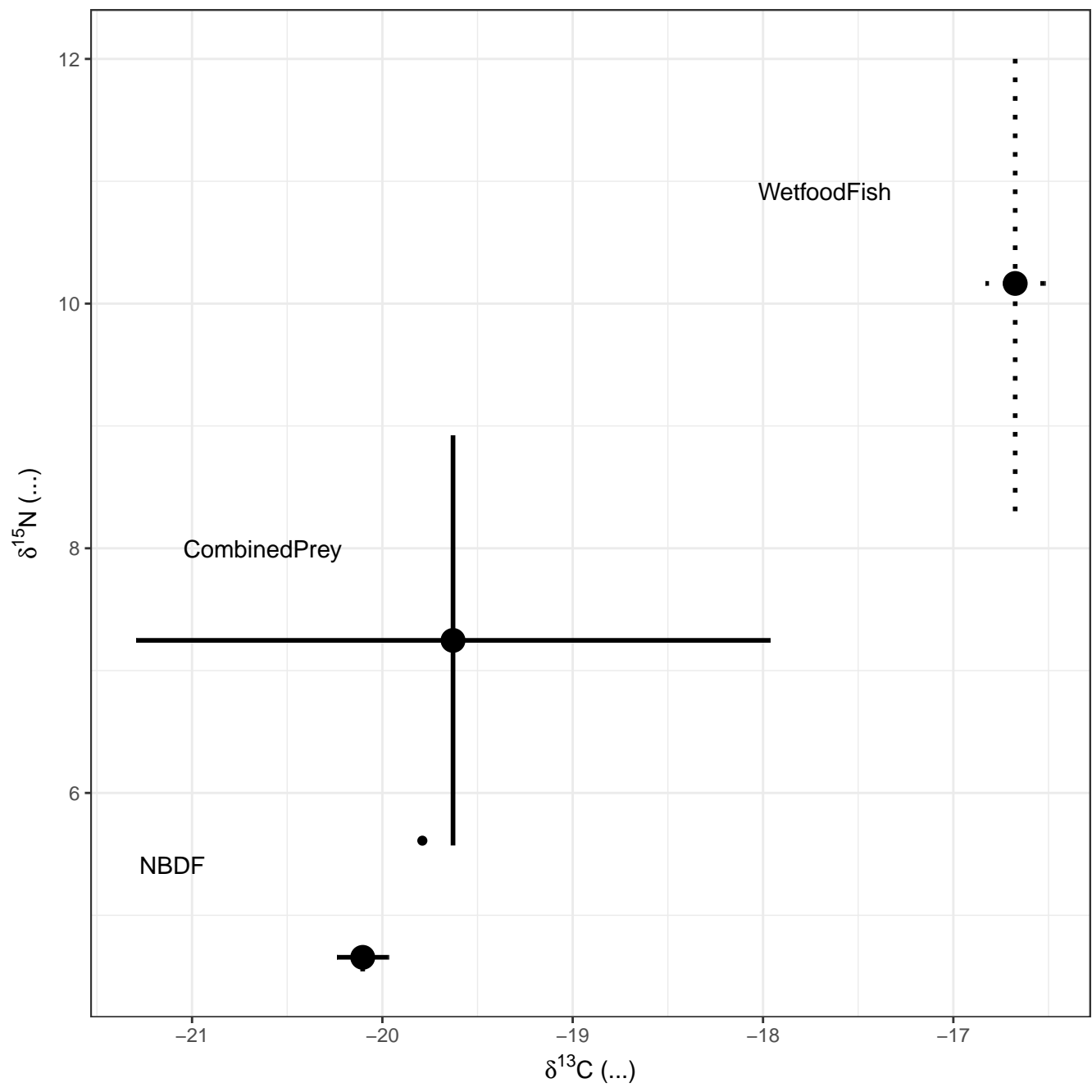

Supplement: Supplemental Information 2 — Contains isospace plot generated in MixSIAR for each cat in analysis. [file peerj-08-8337-s007.zip › Isospace plots/Smattie_isospace combadj.pdf]

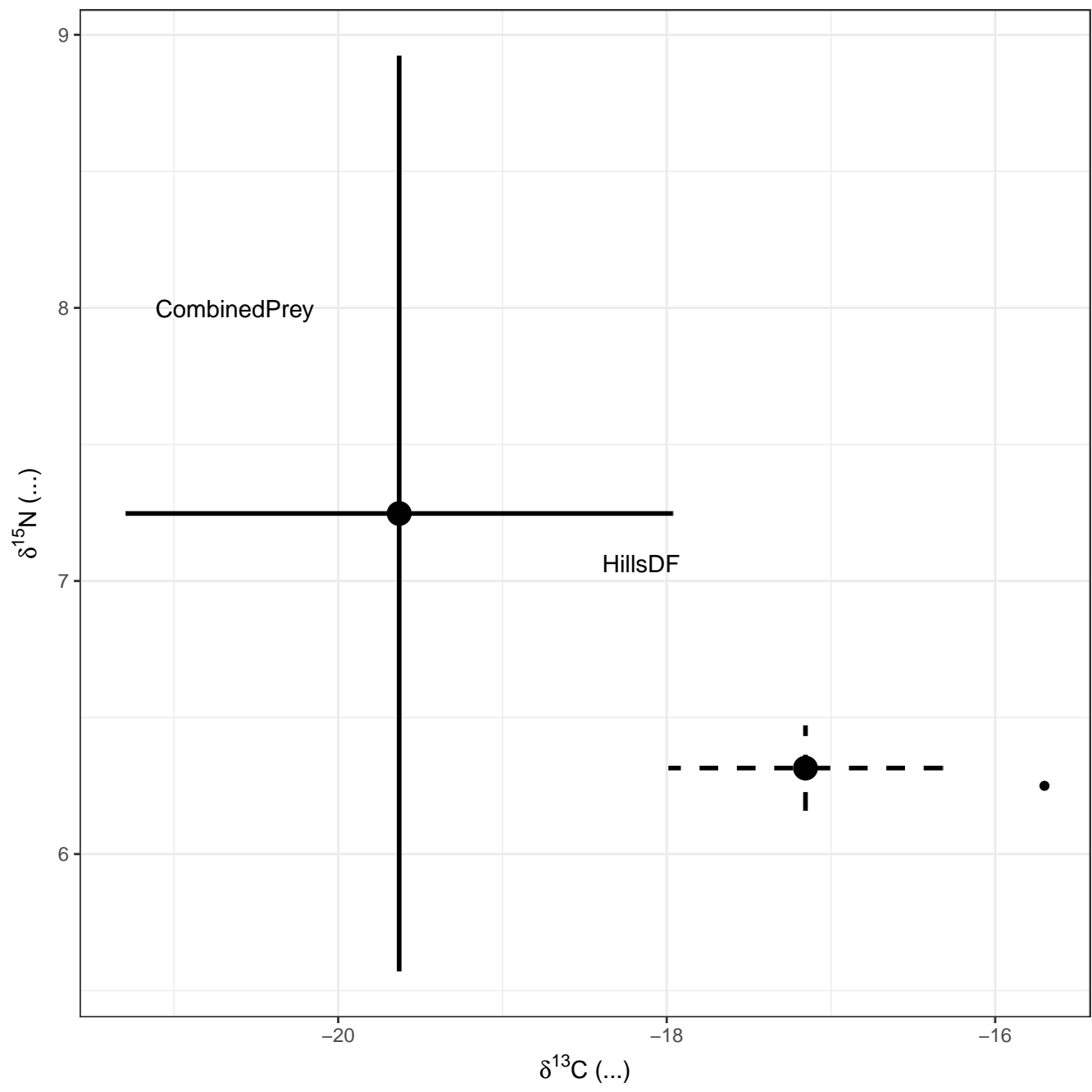

Supplement: Supplemental Information 2 — Contains isospace plot generated in MixSIAR for each cat in analysis. [file peerj-08-8337-s007.zip › Isospace plots/Snickerdoodle_isospace combadj.pdf]

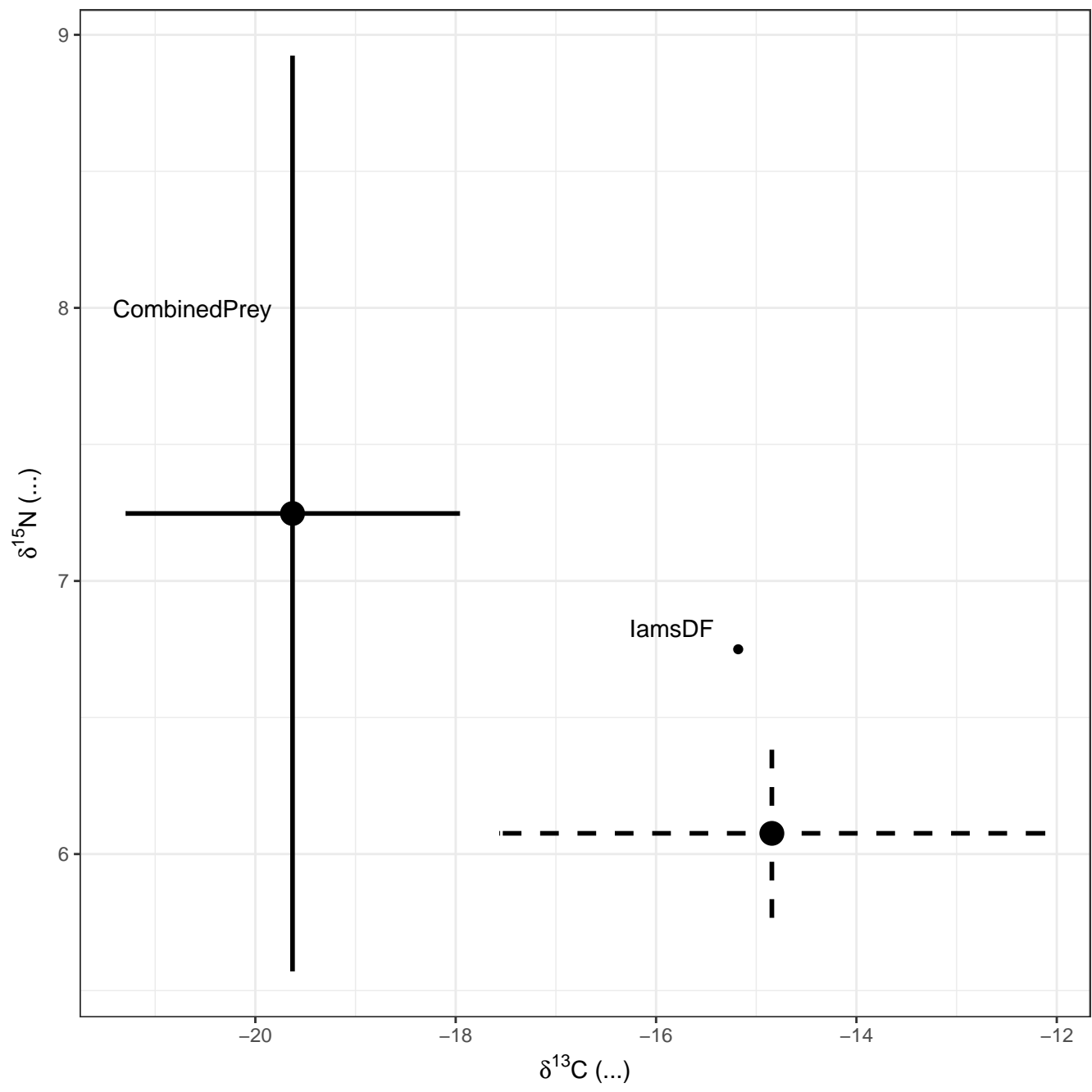

Supplement: Supplemental Information 2 — Contains isospace plot generated in MixSIAR for each cat in analysis. [file peerj-08-8337-s007.zip › Isospace plots/SunnyB_isospace combadj.pdf]

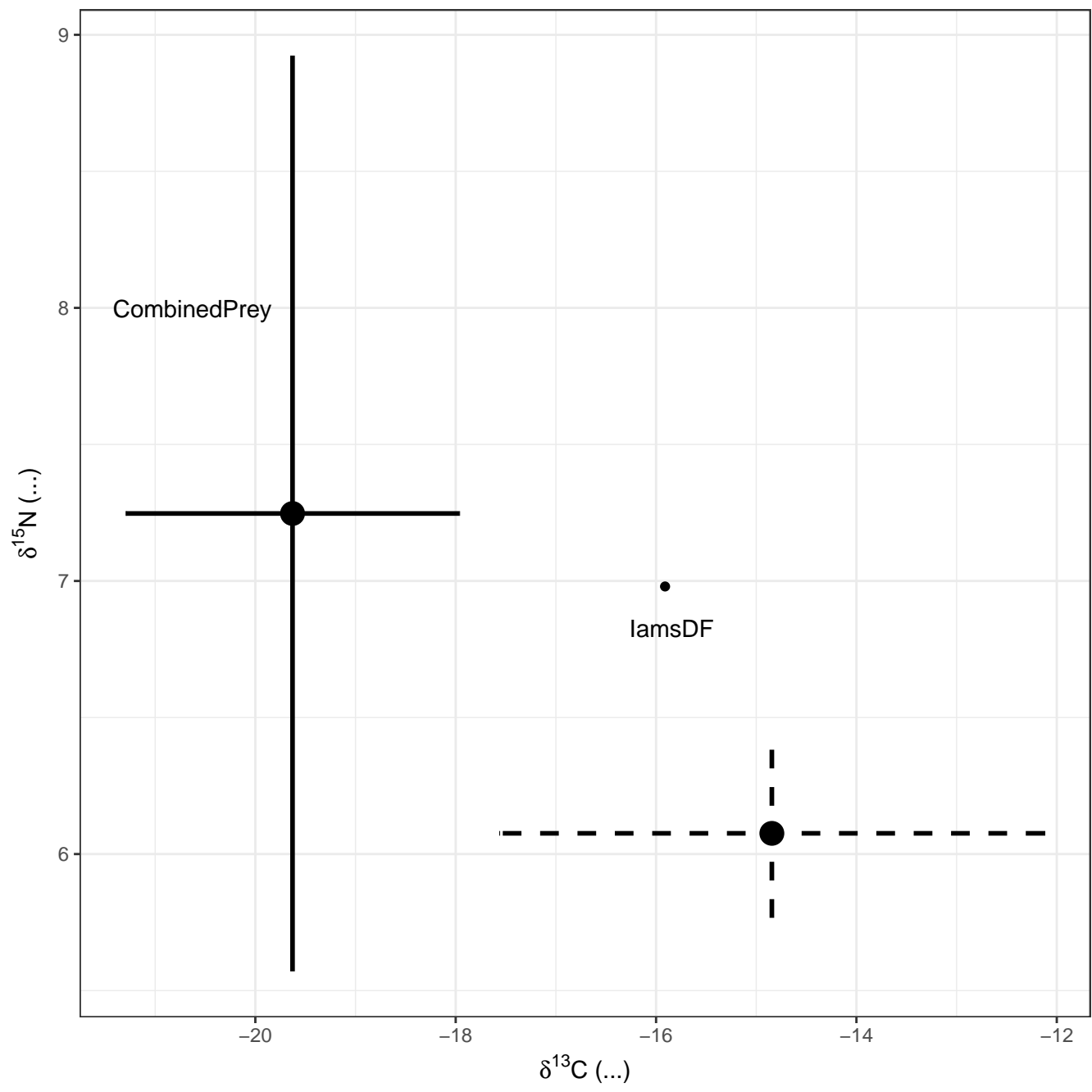

Supplement: Supplemental Information 2 — Contains isospace plot generated in MixSIAR for each cat in analysis. [file peerj-08-8337-s007.zip › Isospace plots/SunnyZ_isospace combadj.pdf]

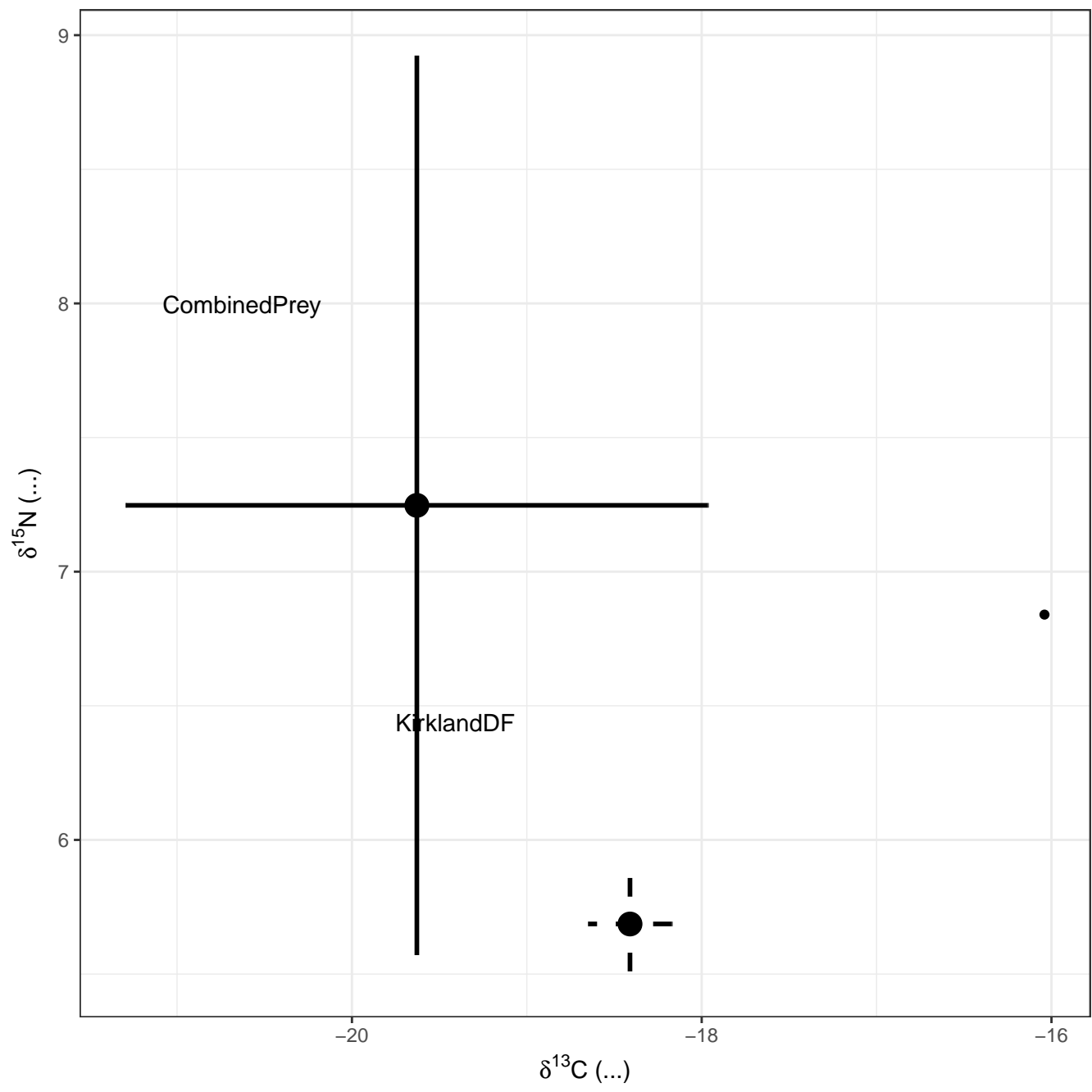

Supplement: Supplemental Information 2 — Contains isospace plot generated in MixSIAR for each cat in analysis. [file peerj-08-8337-s007.zip › Isospace plots/Tallulah_isospace combadj.pdf]

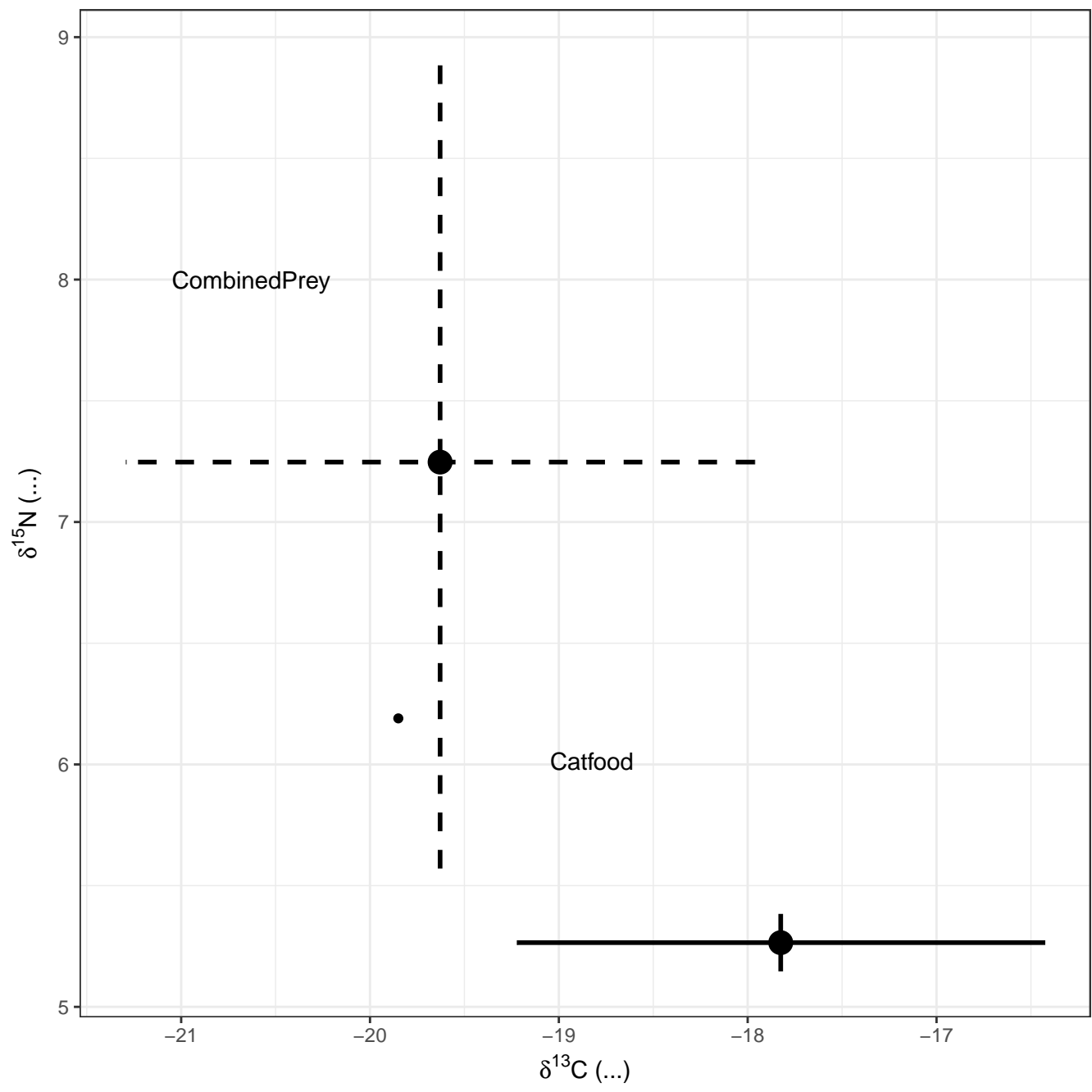

Supplement: Supplemental Information 2 — Contains isospace plot generated in MixSIAR for each cat in analysis. [file peerj-08-8337-s007.zip › Isospace plots/Tara_isospace combadj.pdf]

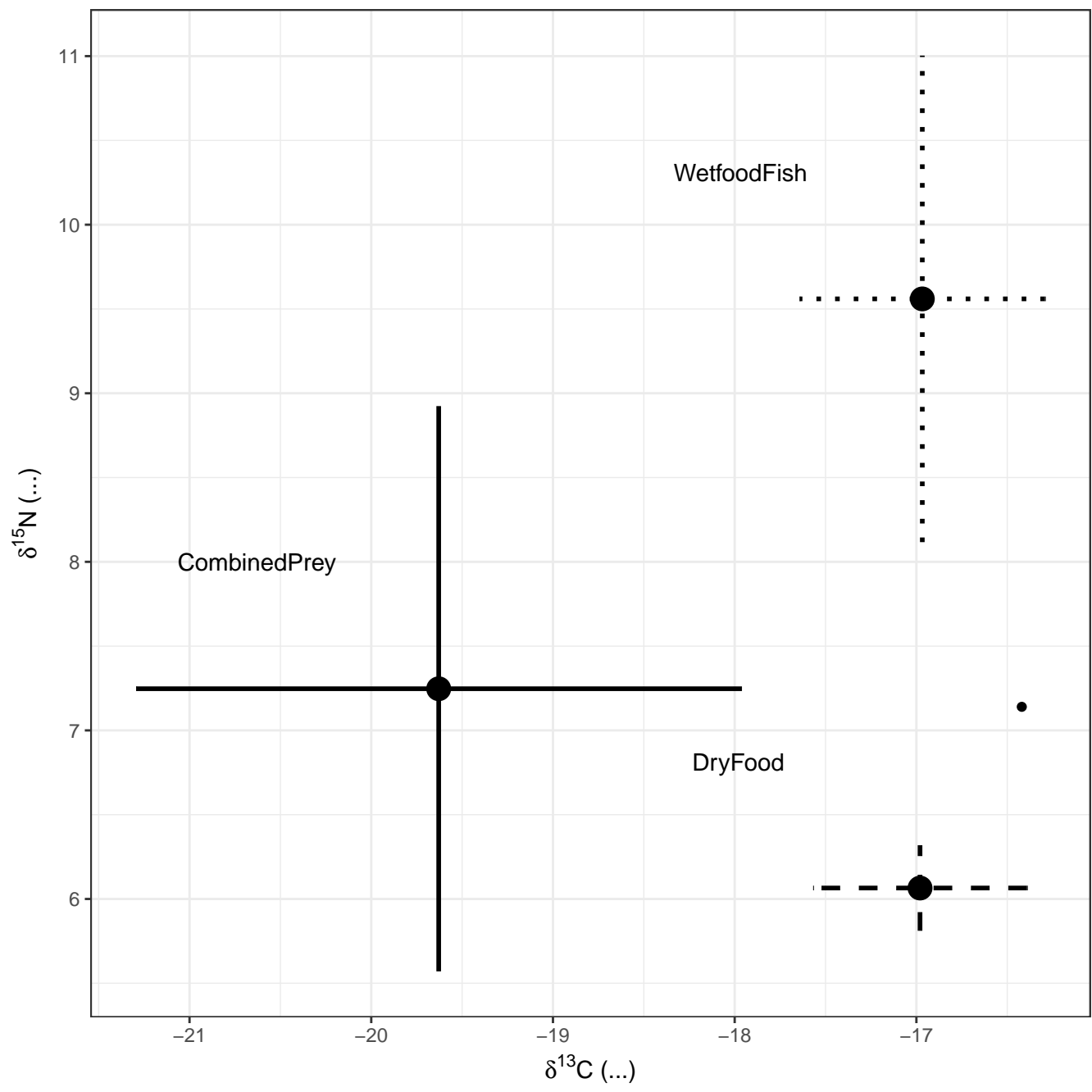

Supplement: Supplemental Information 2 — Contains isospace plot generated in MixSIAR for each cat in analysis. [file peerj-08-8337-s007.zip › Isospace plots/Tex_isospace combadj.pdf]

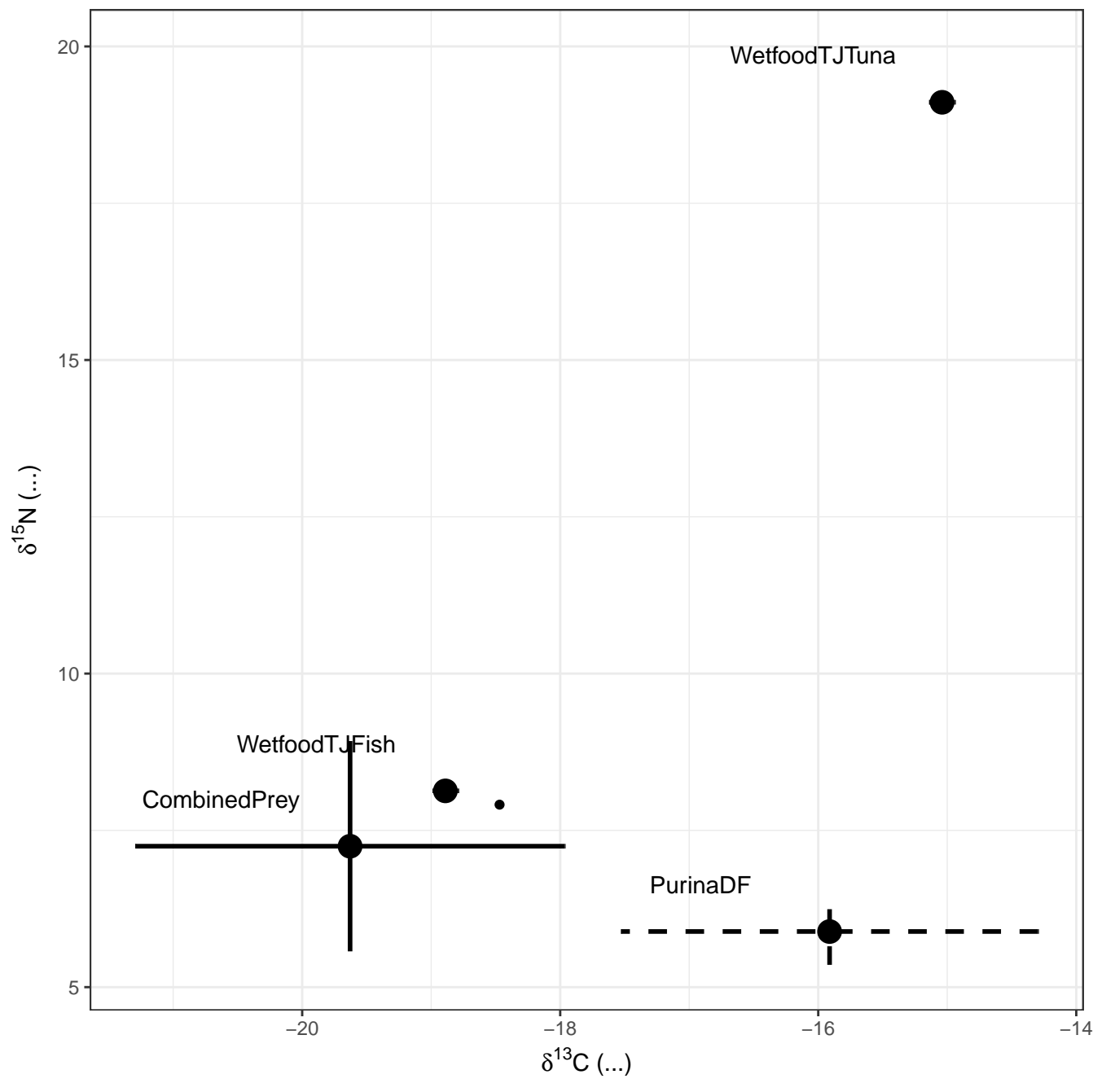

Supplement: Supplemental Information 2 — Contains isospace plot generated in MixSIAR for each cat in analysis. [file peerj-08-8337-s007.zip › Isospace plots/Tiger_isospace combadj.pdf]

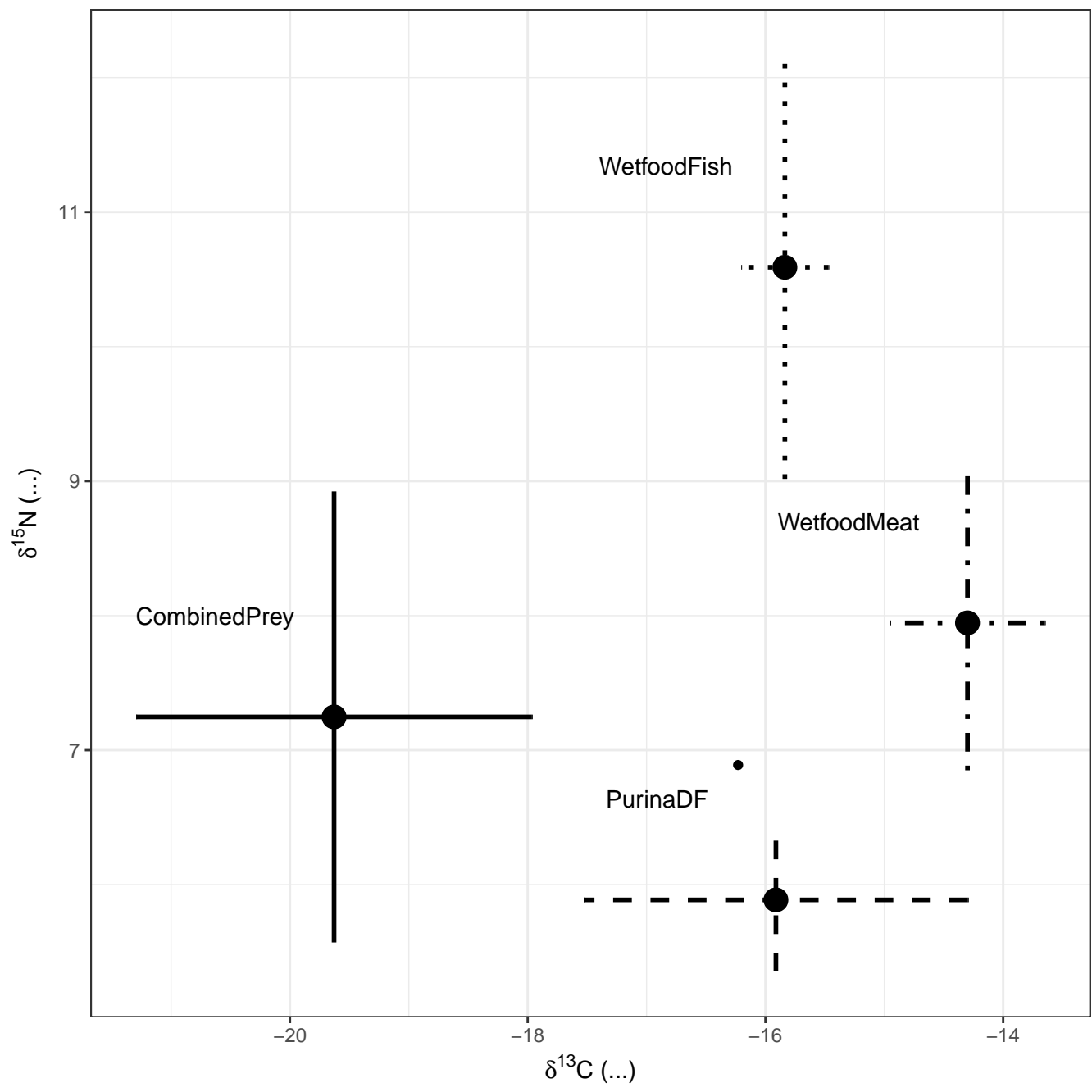

Supplement: Supplemental Information 2 — Contains isospace plot generated in MixSIAR for each cat in analysis. [file peerj-08-8337-s007.zip › Isospace plots/TonkaA_isospace combadj.pdf]

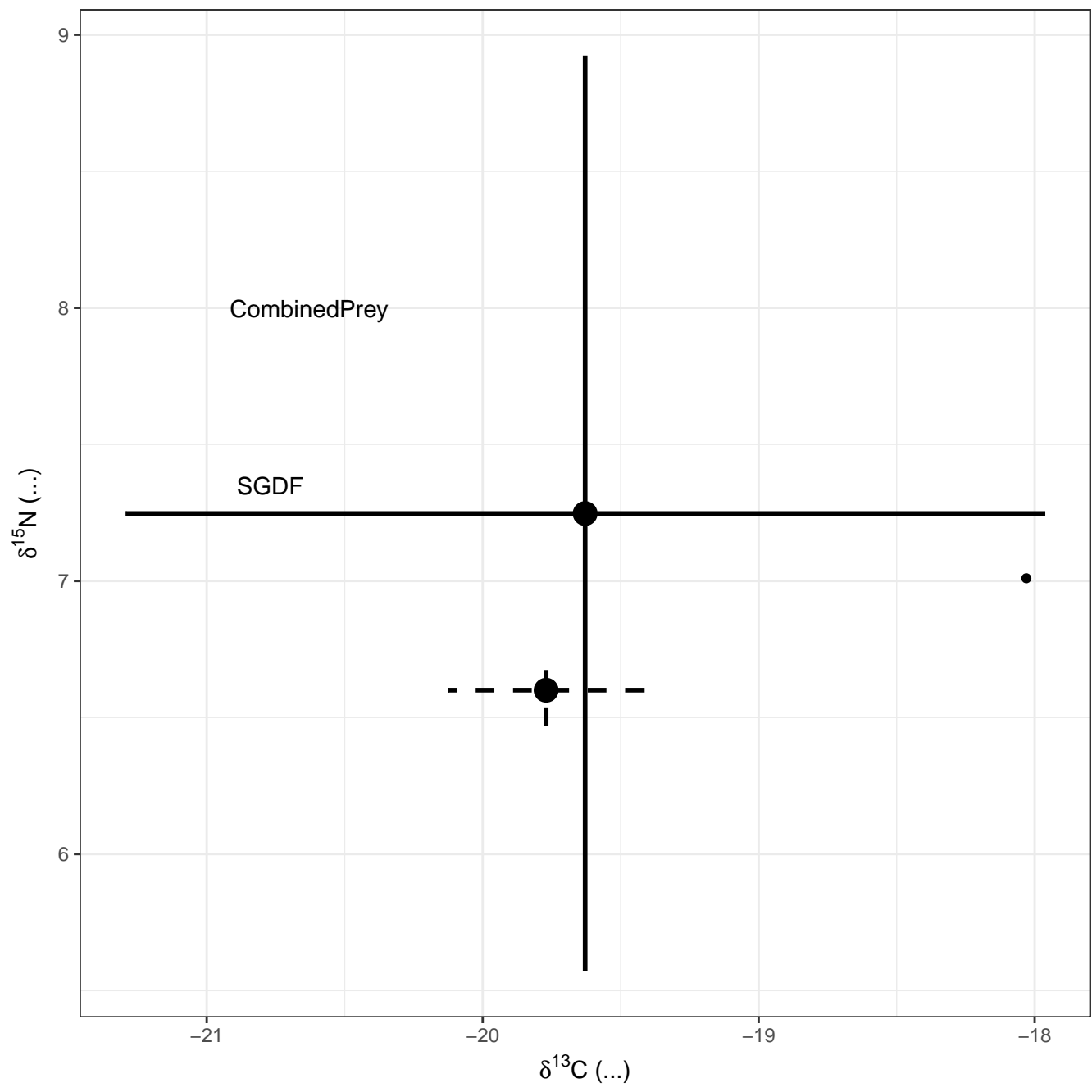

Supplement: Supplemental Information 2 — Contains isospace plot generated in MixSIAR for each cat in analysis. [file peerj-08-8337-s007.zip › Isospace plots/Trinity_isospace combadj.pdf]

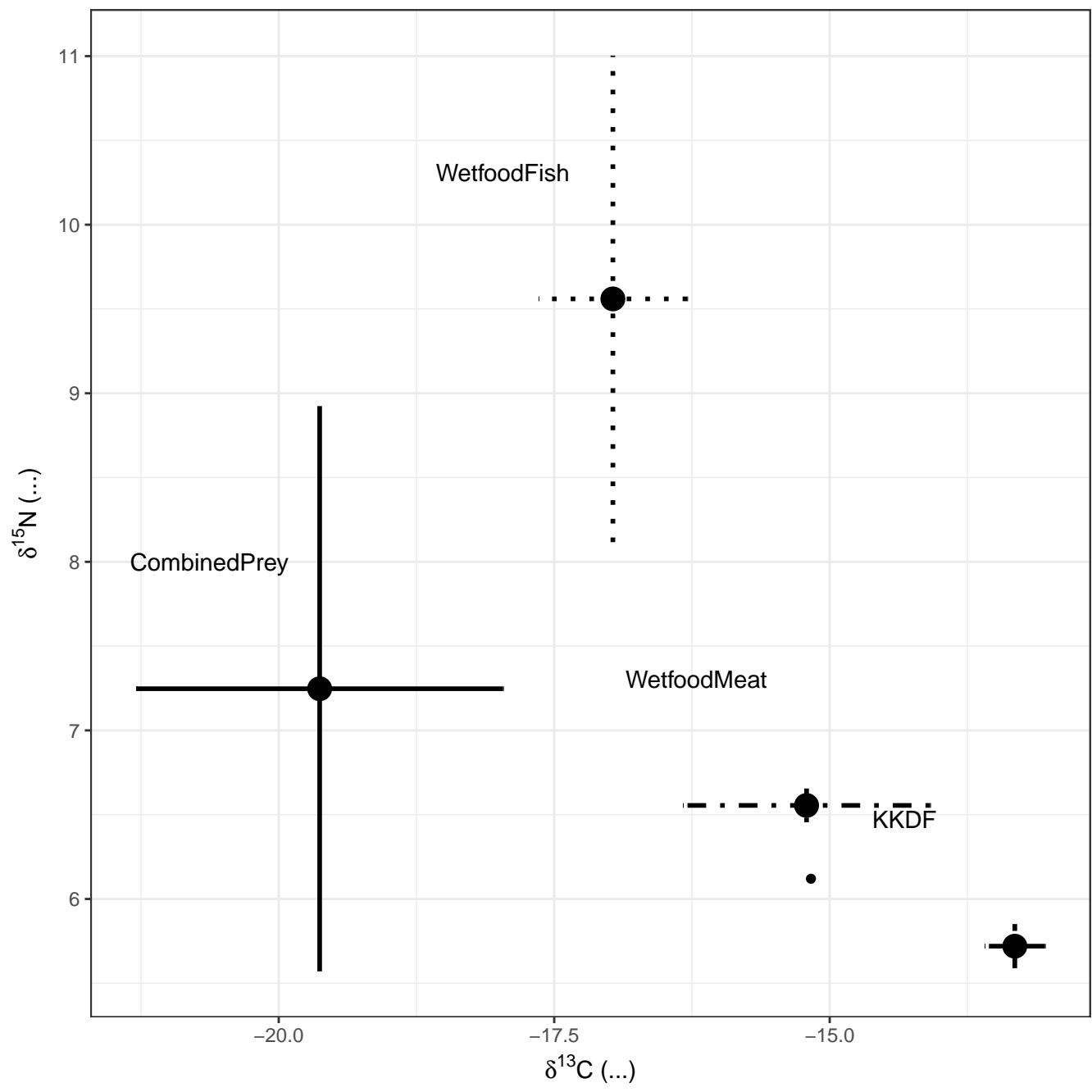

Supplement: Supplemental Information 2 — Contains isospace plot generated in MixSIAR for each cat in analysis. [file peerj-08-8337-s007.zip › Isospace plots/Vacuum_isospace combadj.pdf]

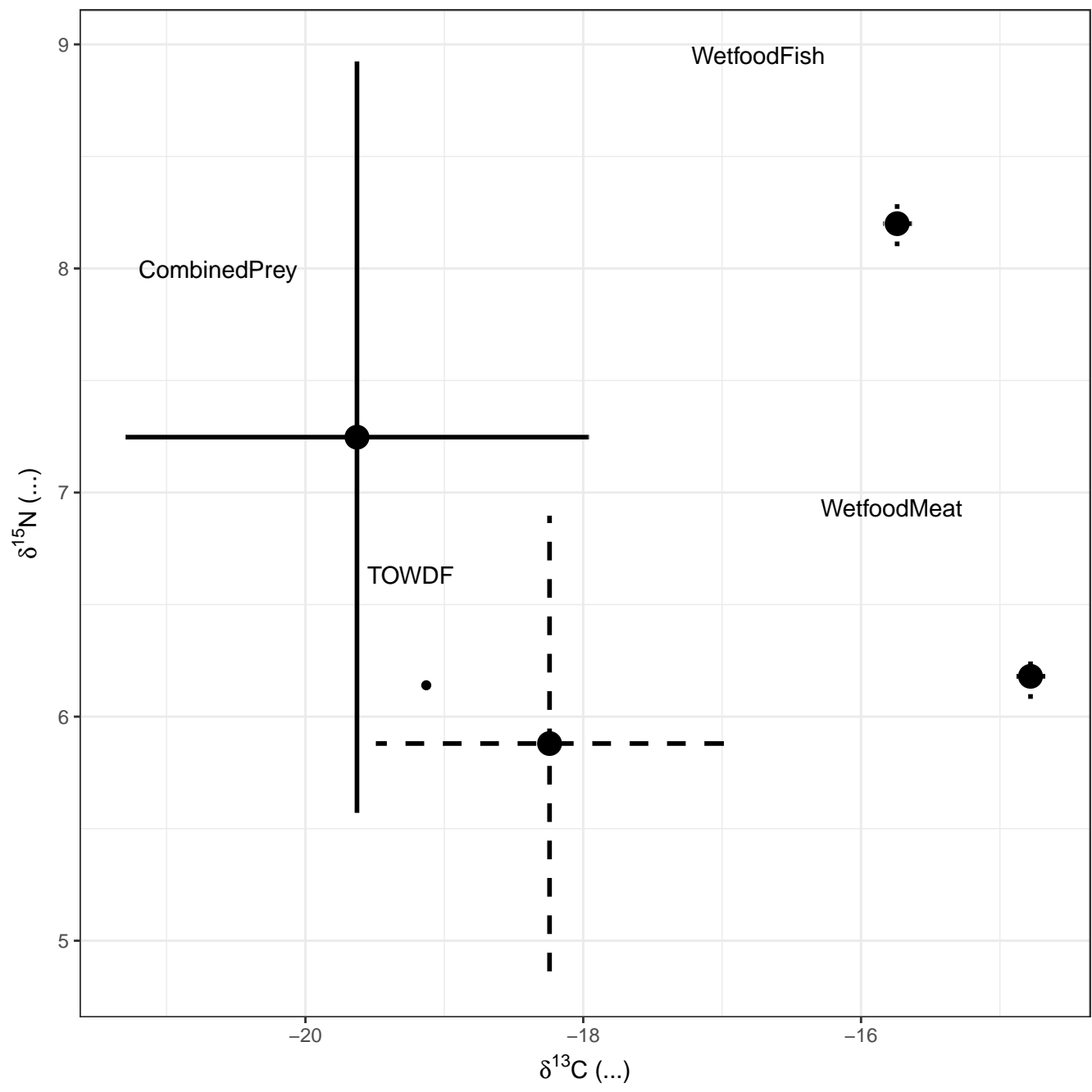

Supplement: Supplemental Information 2 — Contains isospace plot generated in MixSIAR for each cat in analysis. [file peerj-08-8337-s007.zip › Isospace plots/Ziggy_isospace combadj.pdf]

# Overall Population

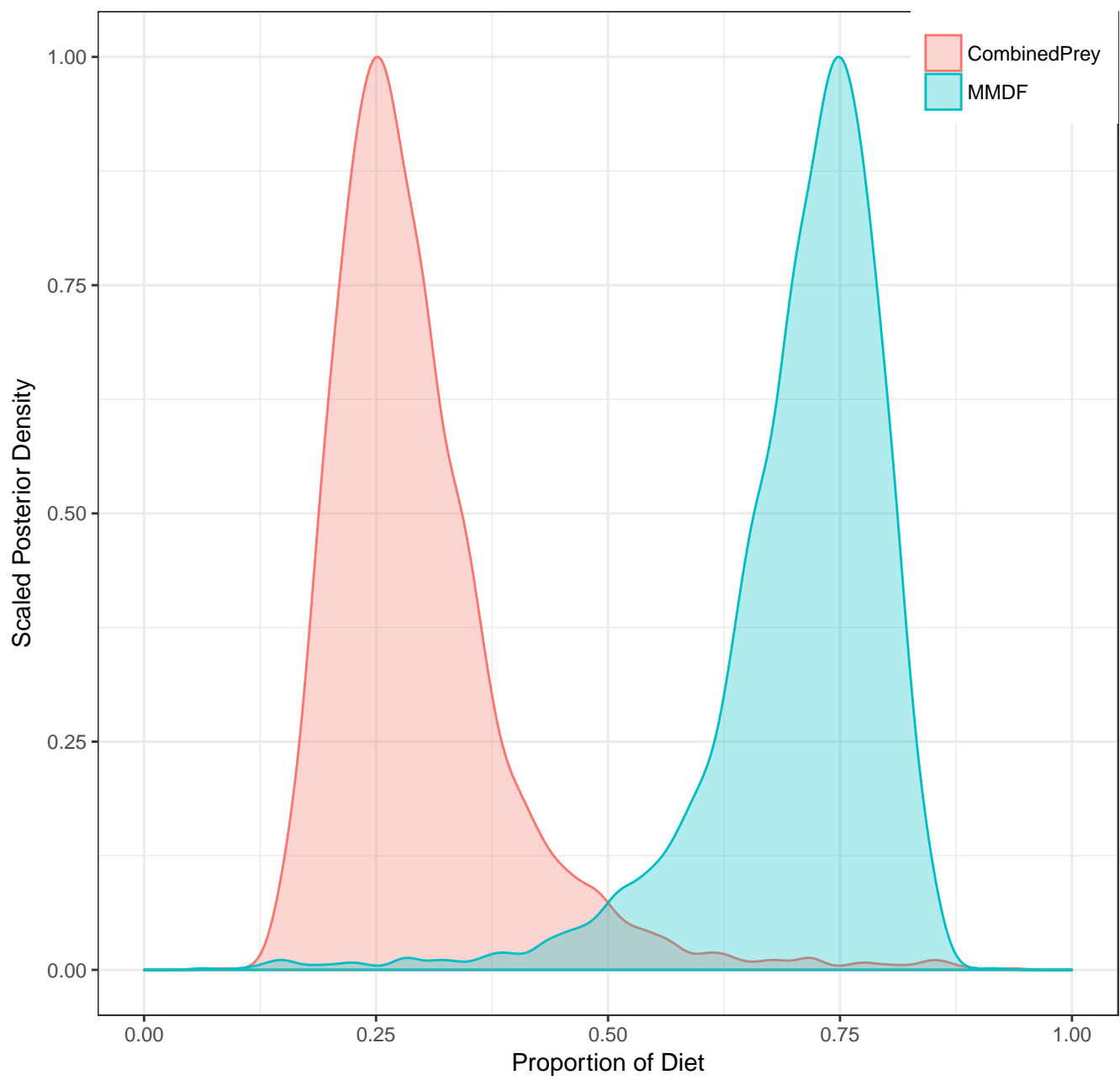

Supplement: Supplemental Information 3 [file peerj-08-8337-s008.zip › Scaled posterior density charts/Amber_proportion combadj.pdf]

Overall Population

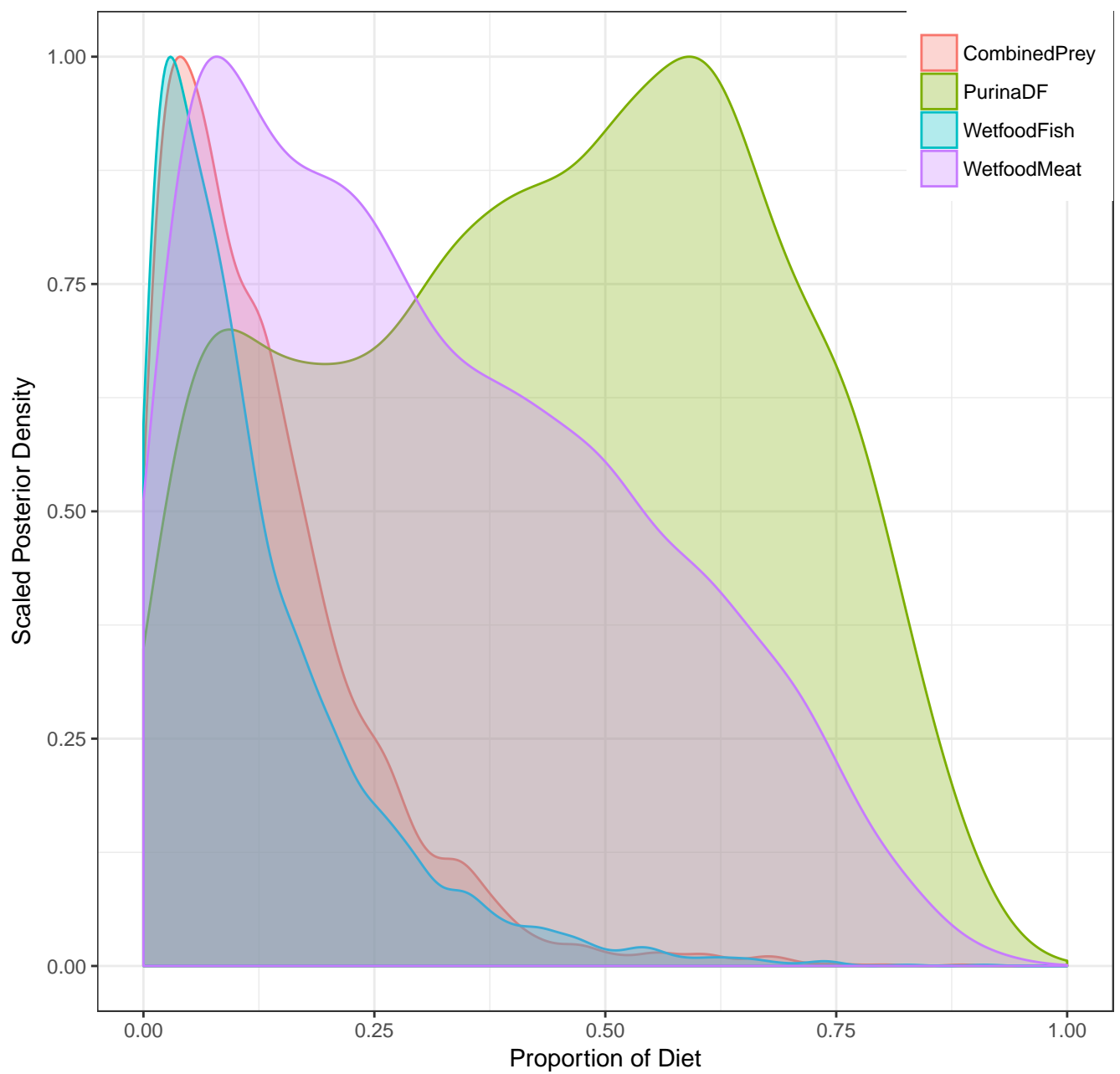

Supplement: Supplemental Information 3 [file peerj-08-8337-s008.zip › Scaled posterior density charts/Banjo_proportion combadj.pdf]

# Overall Population

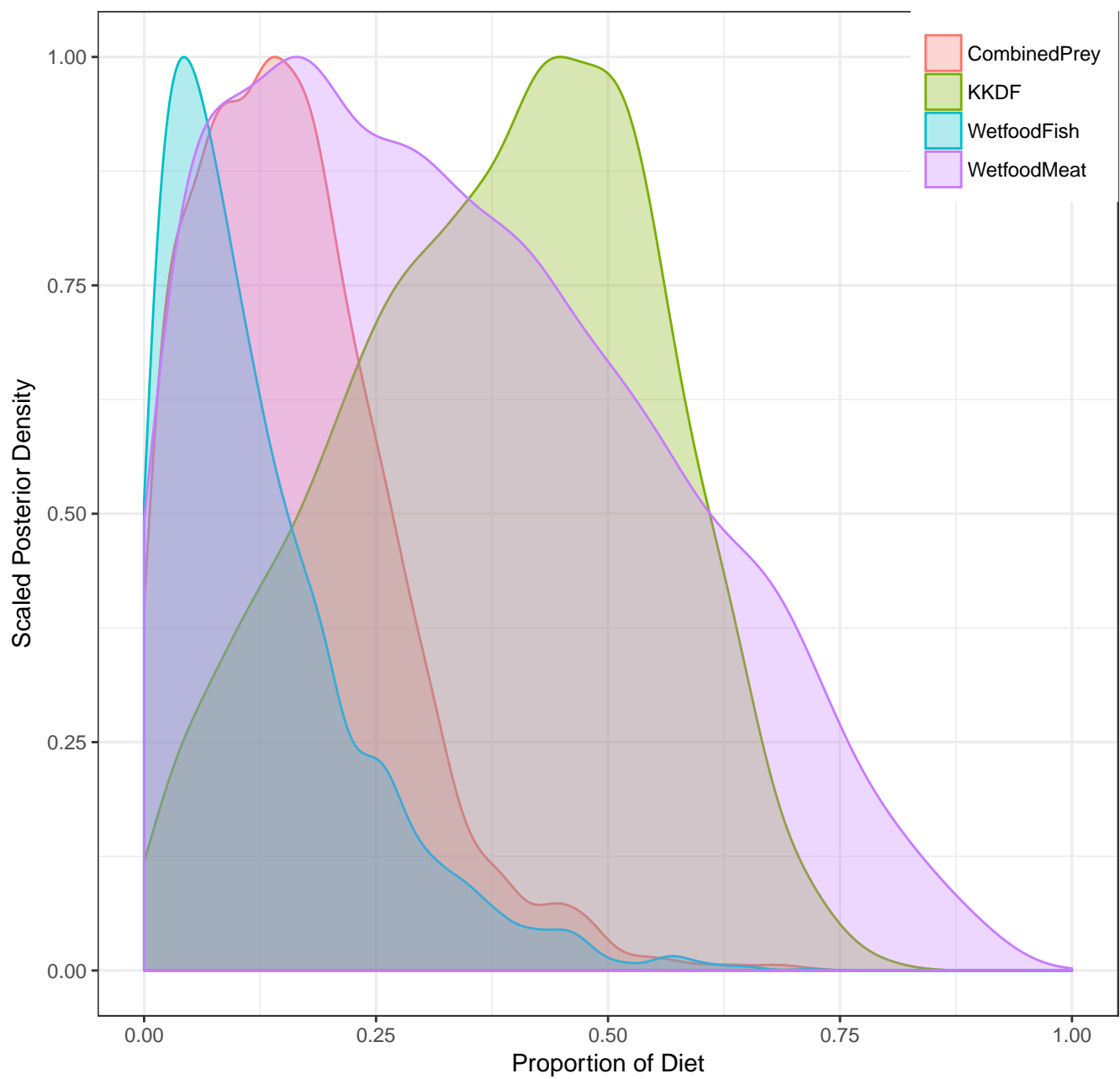

Supplement: Supplemental Information 3 [file peerj-08-8337-s008.zip › Scaled posterior density charts/Beatle_proportion combadj.pdf]

# Overall Population

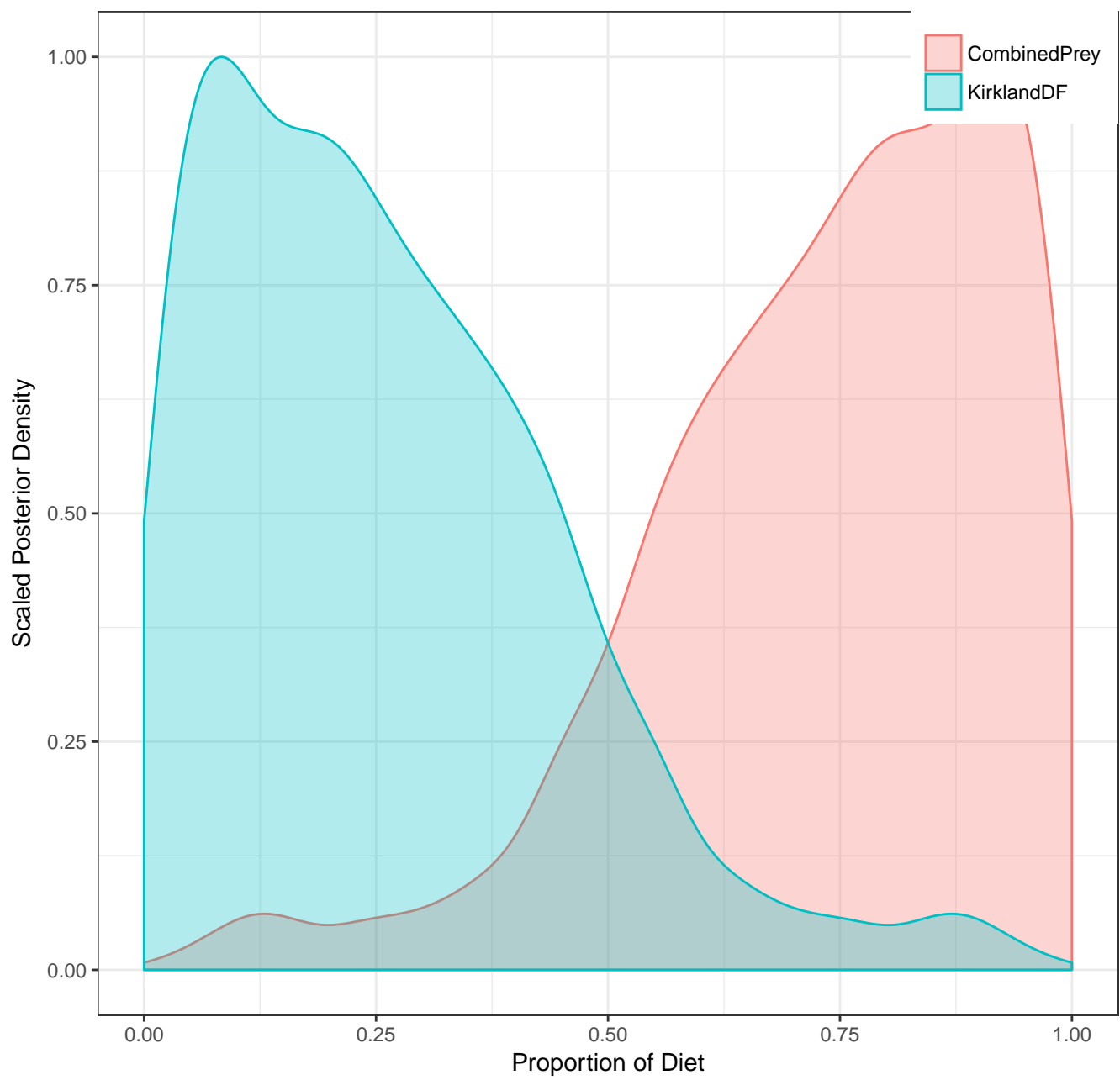

Supplement: Supplemental Information 3 [file peerj-08-8337-s008.zip › Scaled posterior density charts/CatnissE_proportion combadj.pdf]

Overall Population

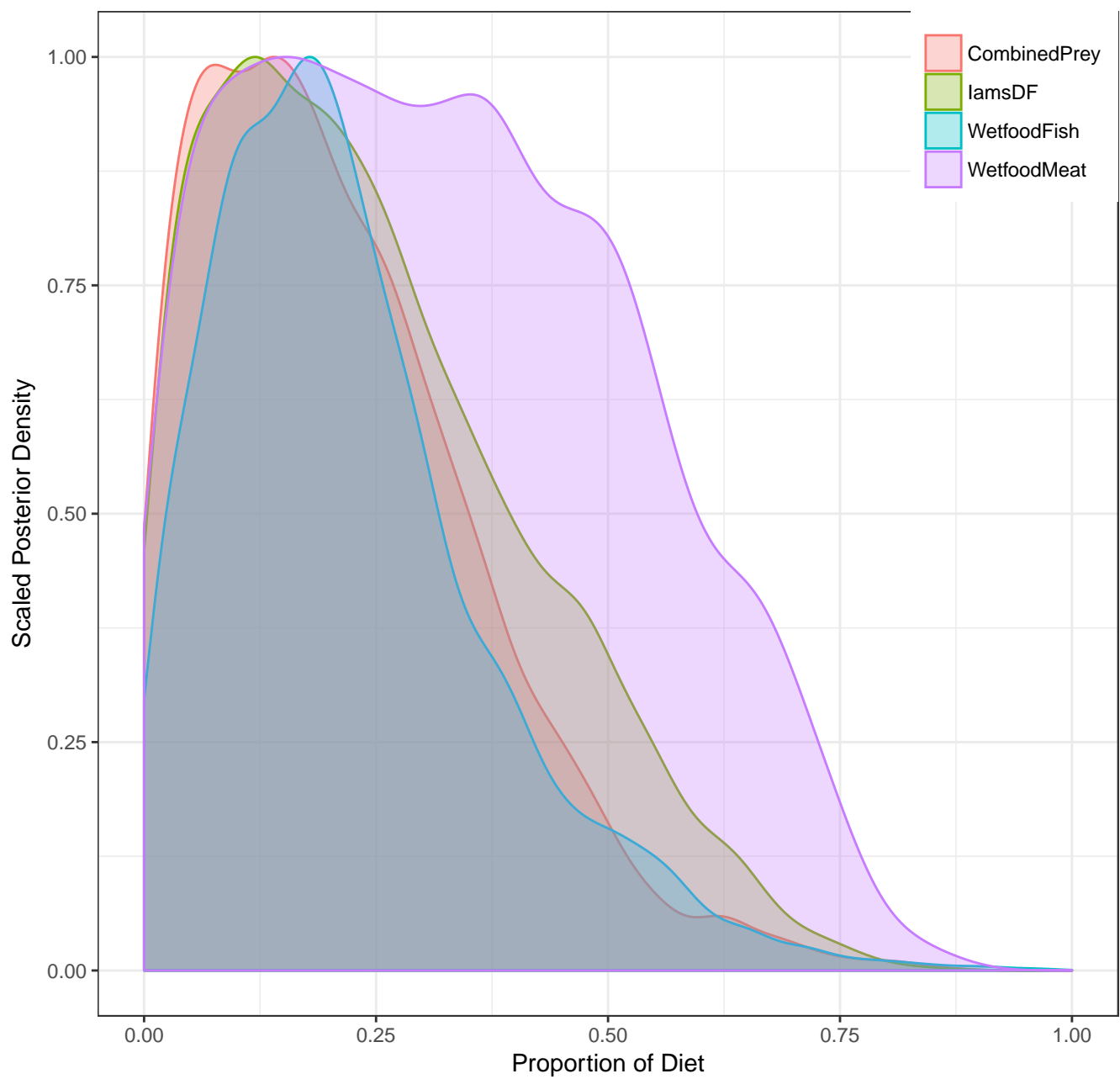

Supplement: Supplemental Information 3 [file peerj-08-8337-s008.zip › Scaled posterior density charts/Cayden_proportion combadj.pdf]

# Overall Population

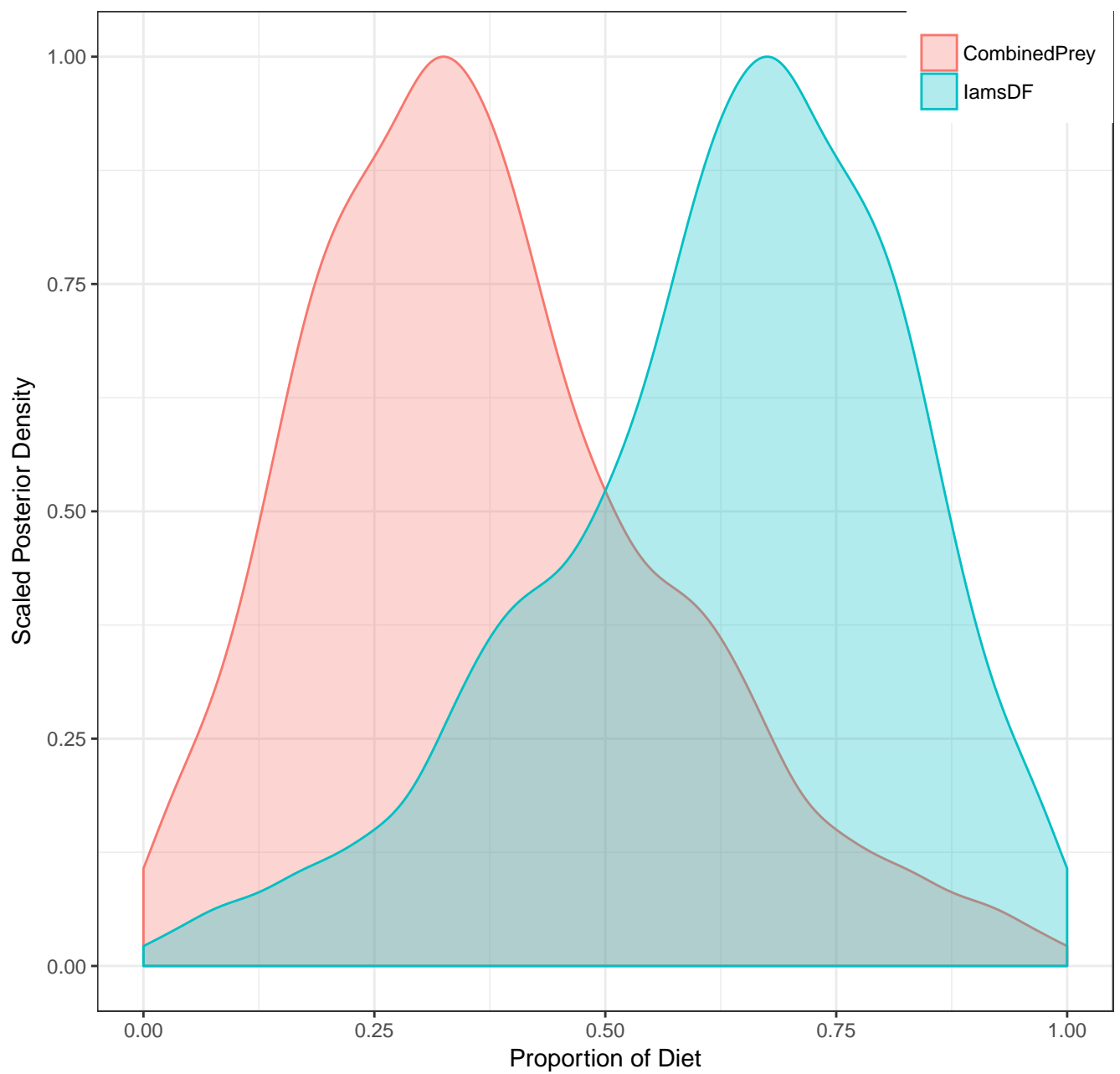

Supplement: Supplemental Information 3 [file peerj-08-8337-s008.zip › Scaled posterior density charts/Climber_proportion combadj.pdf]

# Overall Population

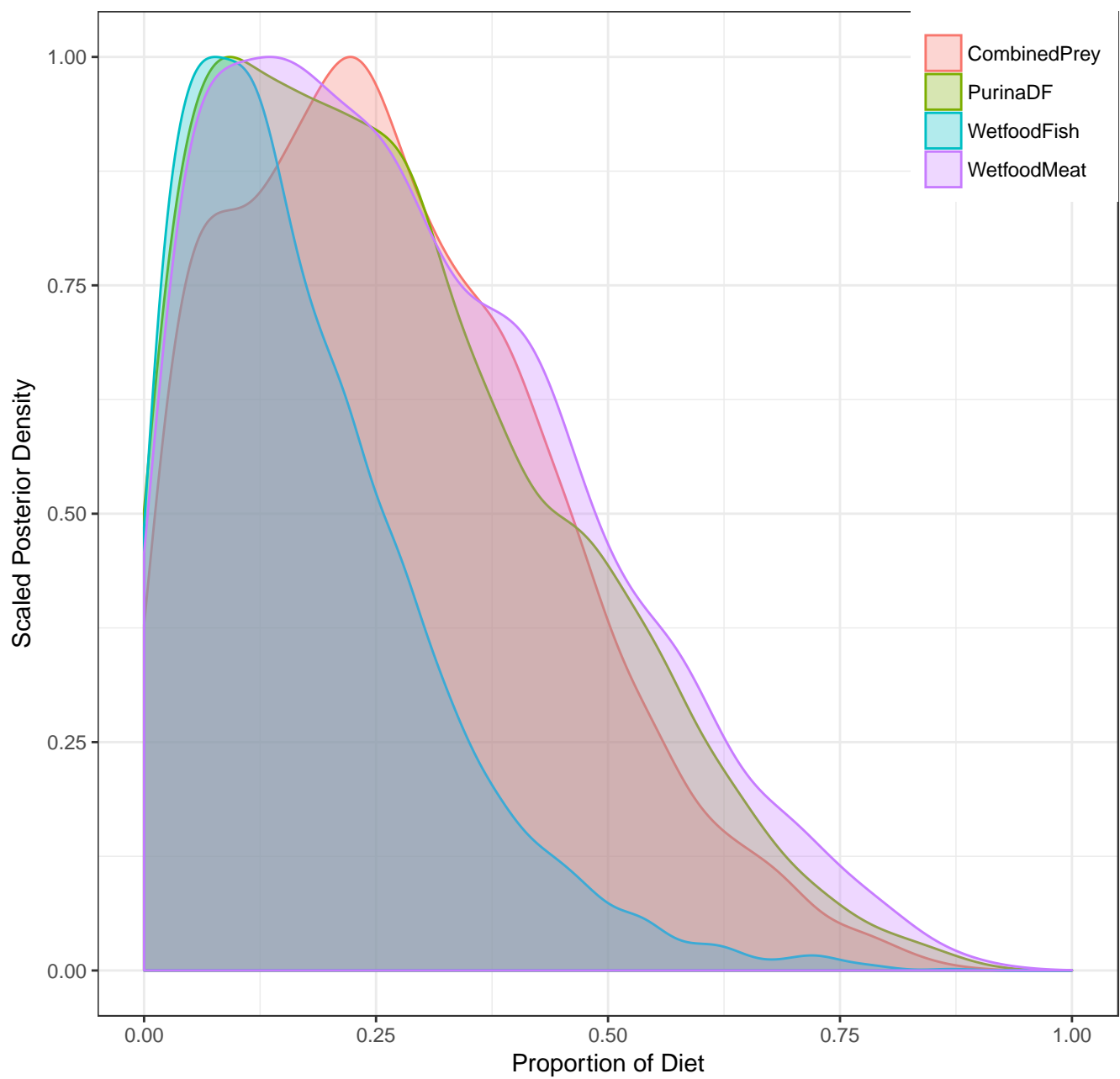

Supplement: Supplemental Information 3 [file peerj-08-8337-s008.zip › Scaled posterior density charts/CptnAmerica_proportion combadj.pdf]

# Overall Population

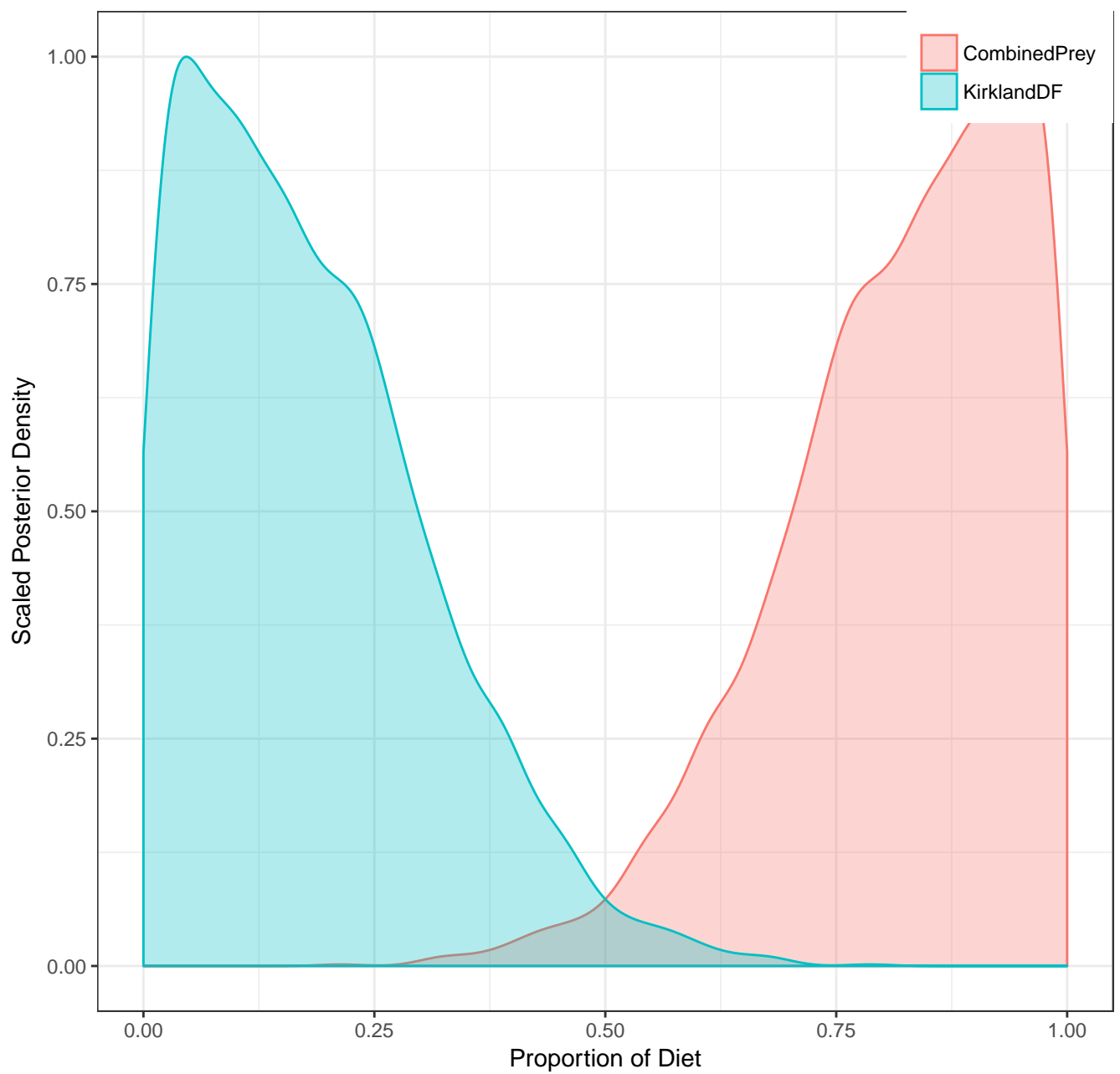

Supplement: Supplemental Information 3 [file peerj-08-8337-s008.zip › Scaled posterior density charts/DCarlos_proportion combadj.pdf]

# Overall Population

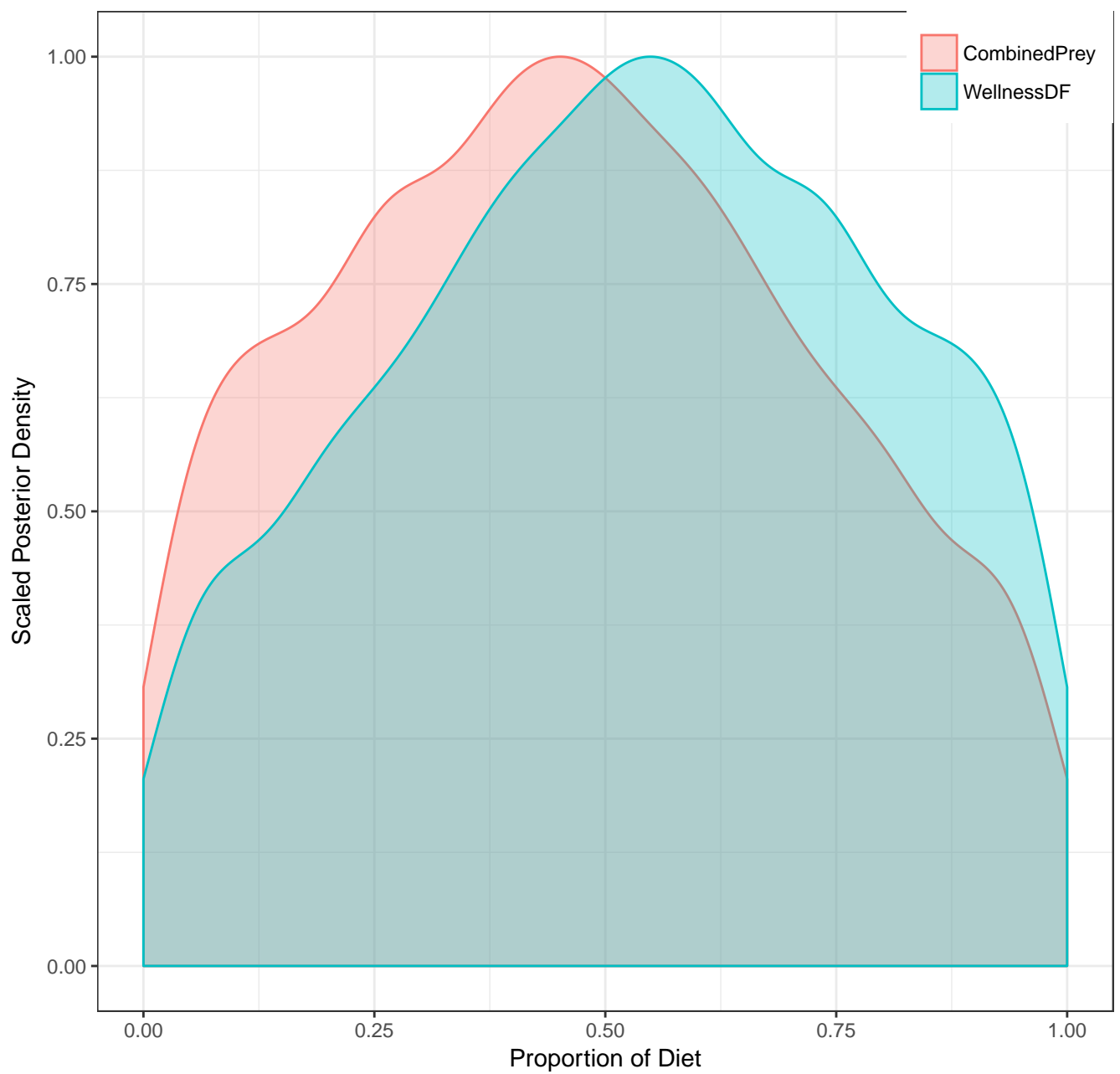

Supplement: Supplemental Information 3 [file peerj-08-8337-s008.zip › Scaled posterior density charts/Fey_proportion combadj.pdf]

# Overall Population

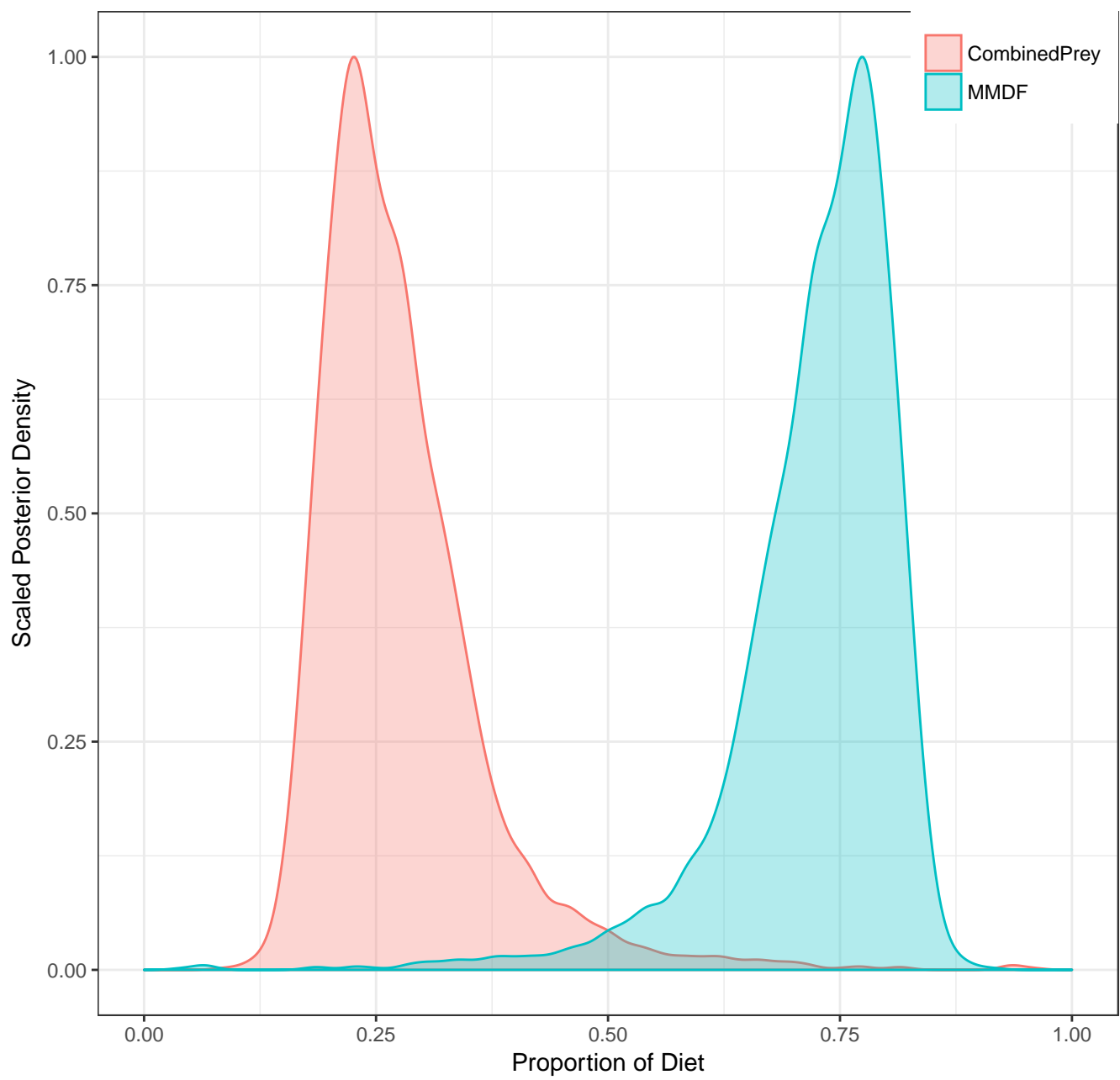

Supplement: Supplemental Information 3 [file peerj-08-8337-s008.zip › Scaled posterior density charts/Fiona_proportion combadj.pdf]

# Overall Population

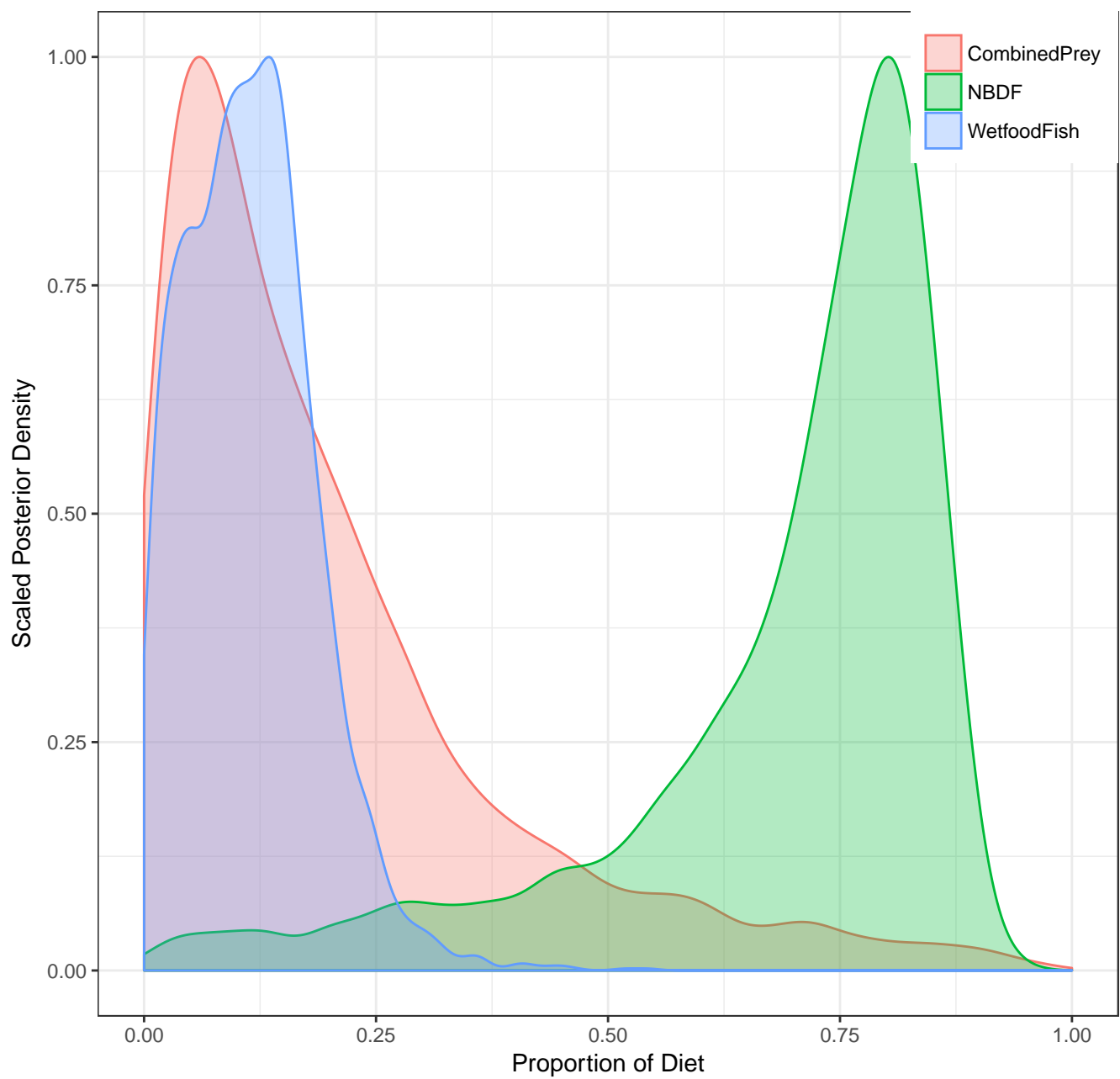

Supplement: Supplemental Information 3 [file peerj-08-8337-s008.zip › Scaled posterior density charts/Gianni_proportion combadj.pdf]

# Overall Population

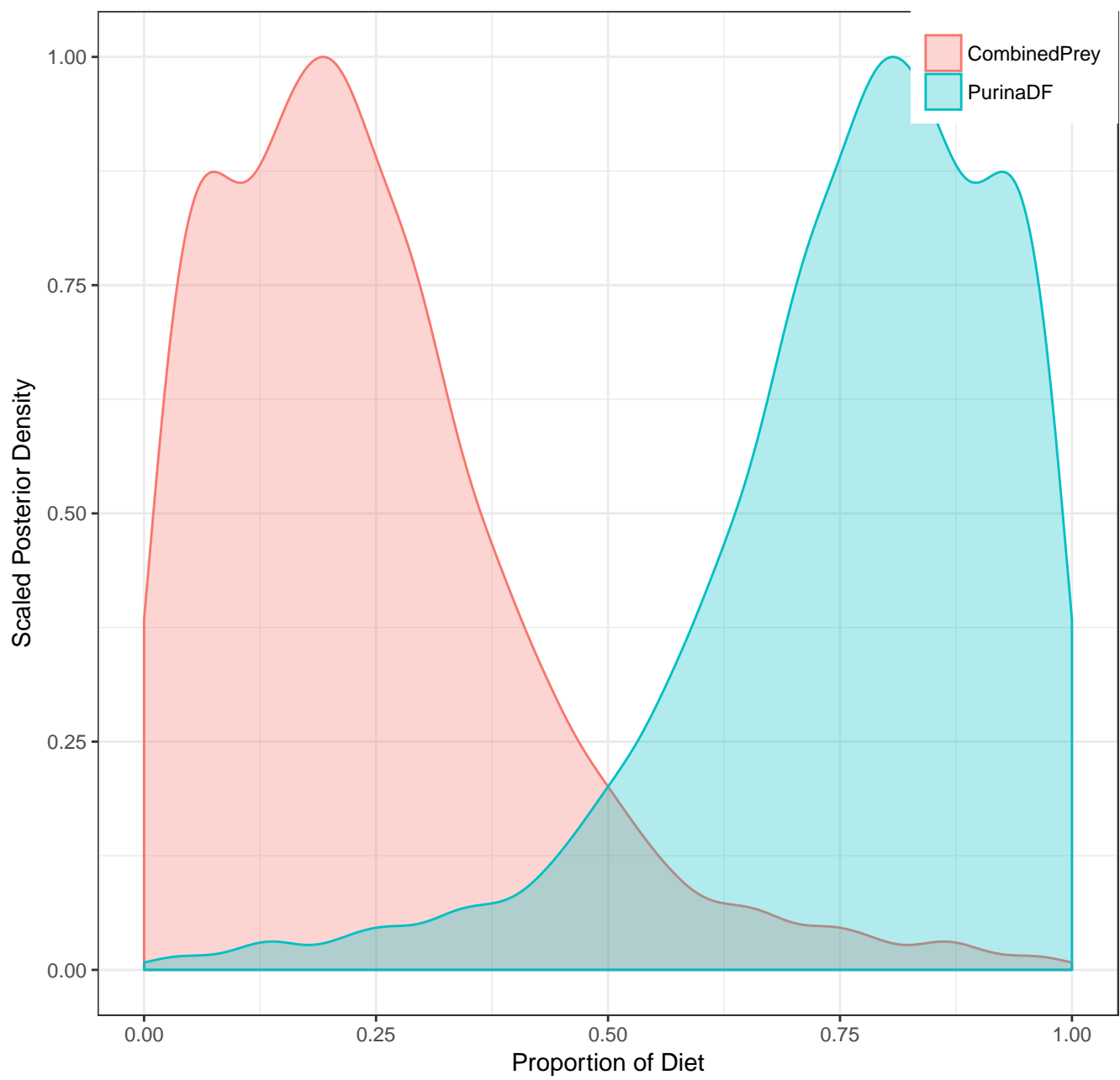

Supplement: Supplemental Information 3 [file peerj-08-8337-s008.zip › Scaled posterior density charts/Gracie_proportion combadj.pdf]

# Overall Population

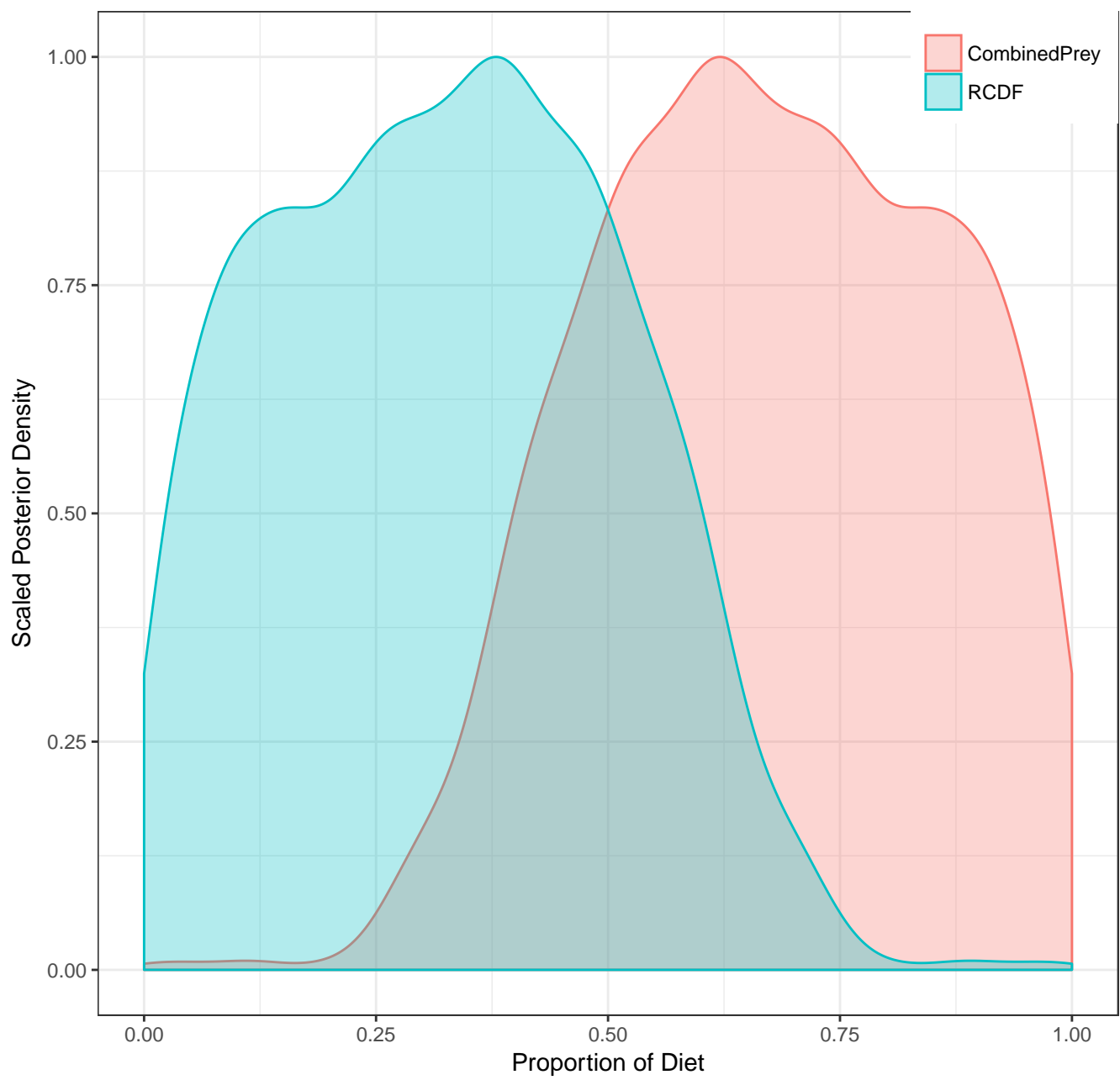

Supplement: Supplemental Information 3 [file peerj-08-8337-s008.zip › Scaled posterior density charts/Hailey_proportion combadj.pdf]

# Overall Population

Scaled Posterior Density

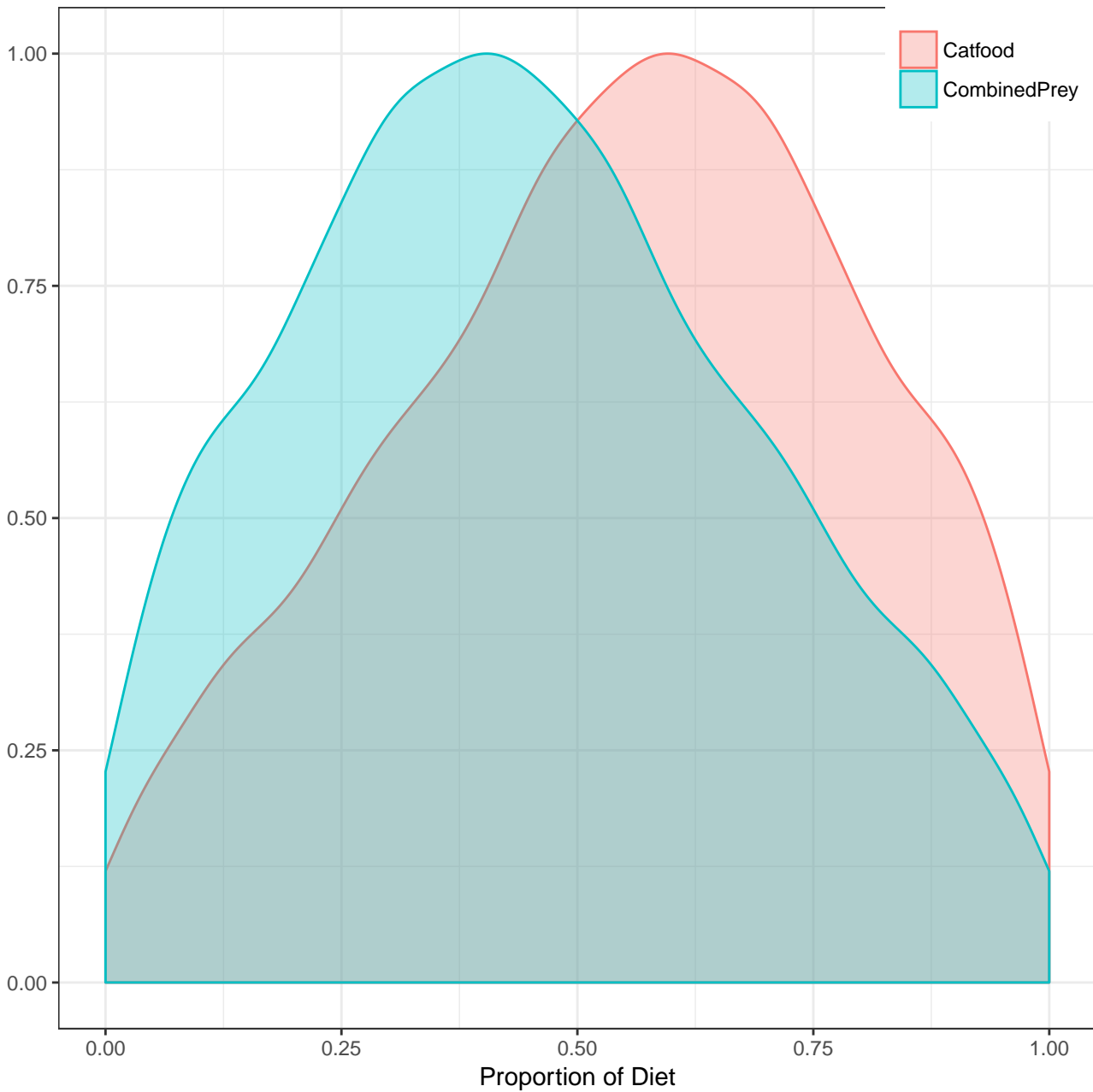

Supplement: Supplemental Information 3 [file peerj-08-8337-s008.zip › Scaled posterior density charts/HazelG_proportion combadj.pdf]

# Overall Population

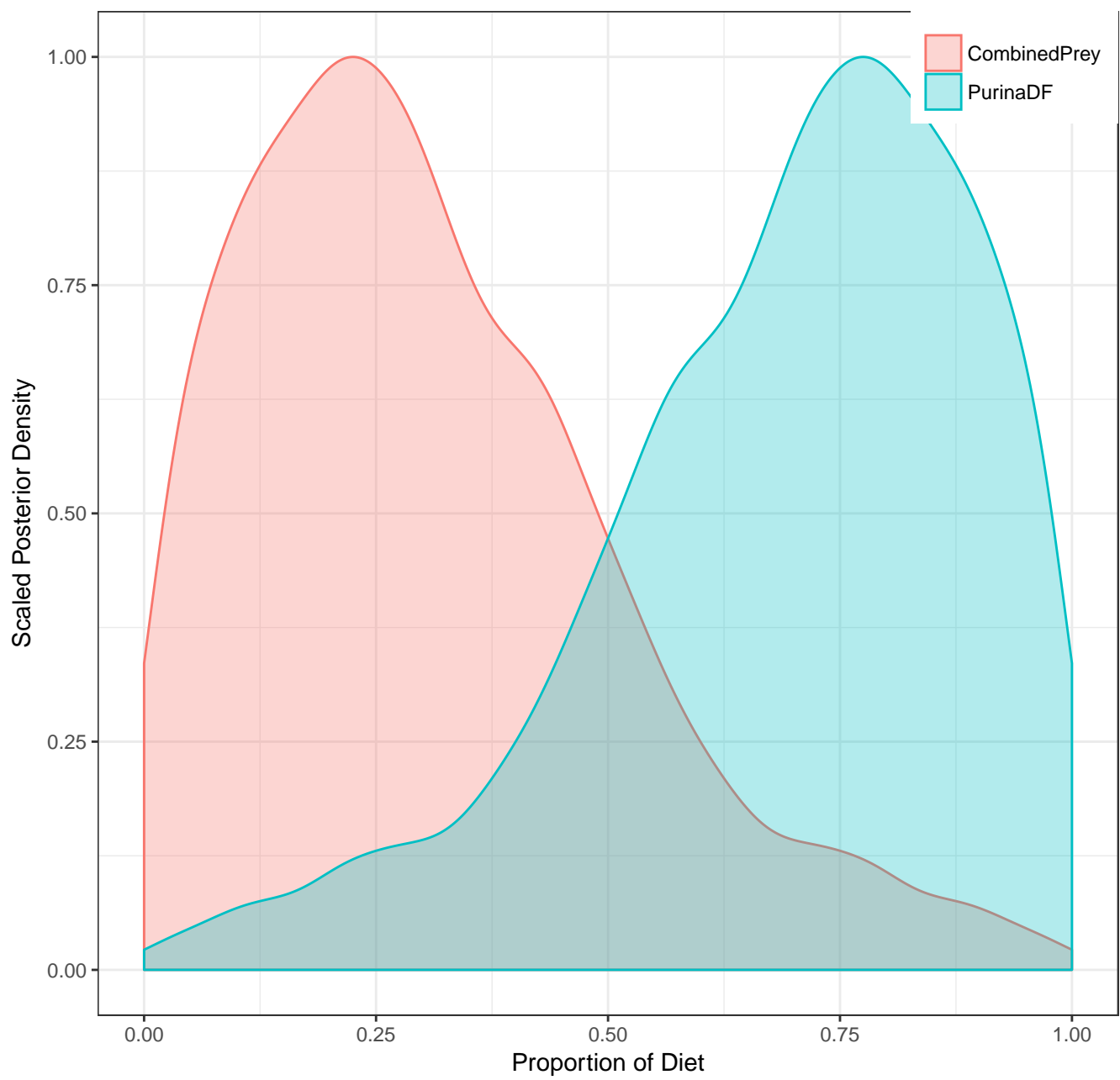

Supplement: Supplemental Information 3 [file peerj-08-8337-s008.zip › Scaled posterior density charts/Hershey_proportion combadj.pdf]

# Overall Population

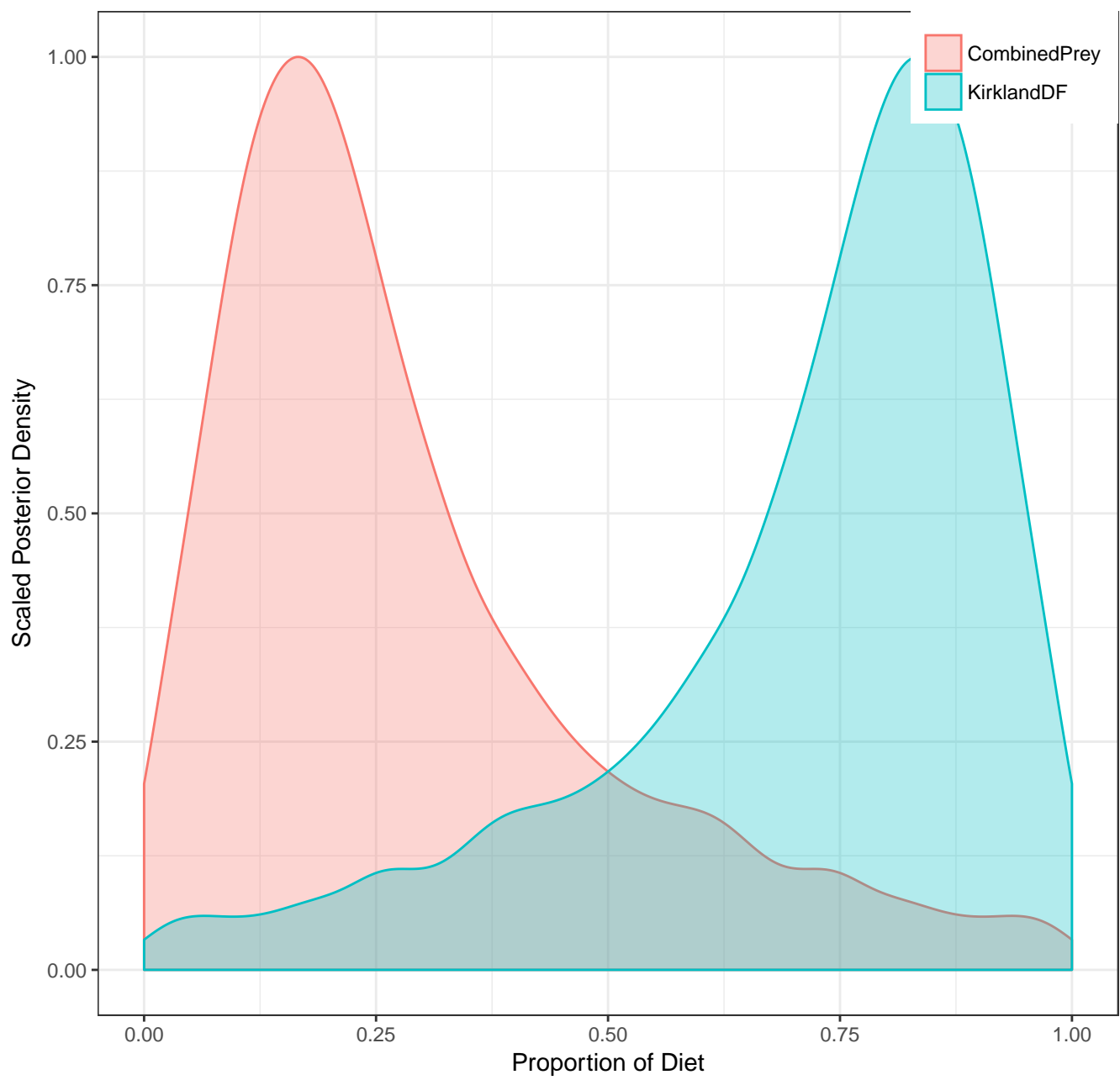

Supplement: Supplemental Information 3 [file peerj-08-8337-s008.zip › Scaled posterior density charts/Jetpack_proportion combadj.pdf]

# Overall Population

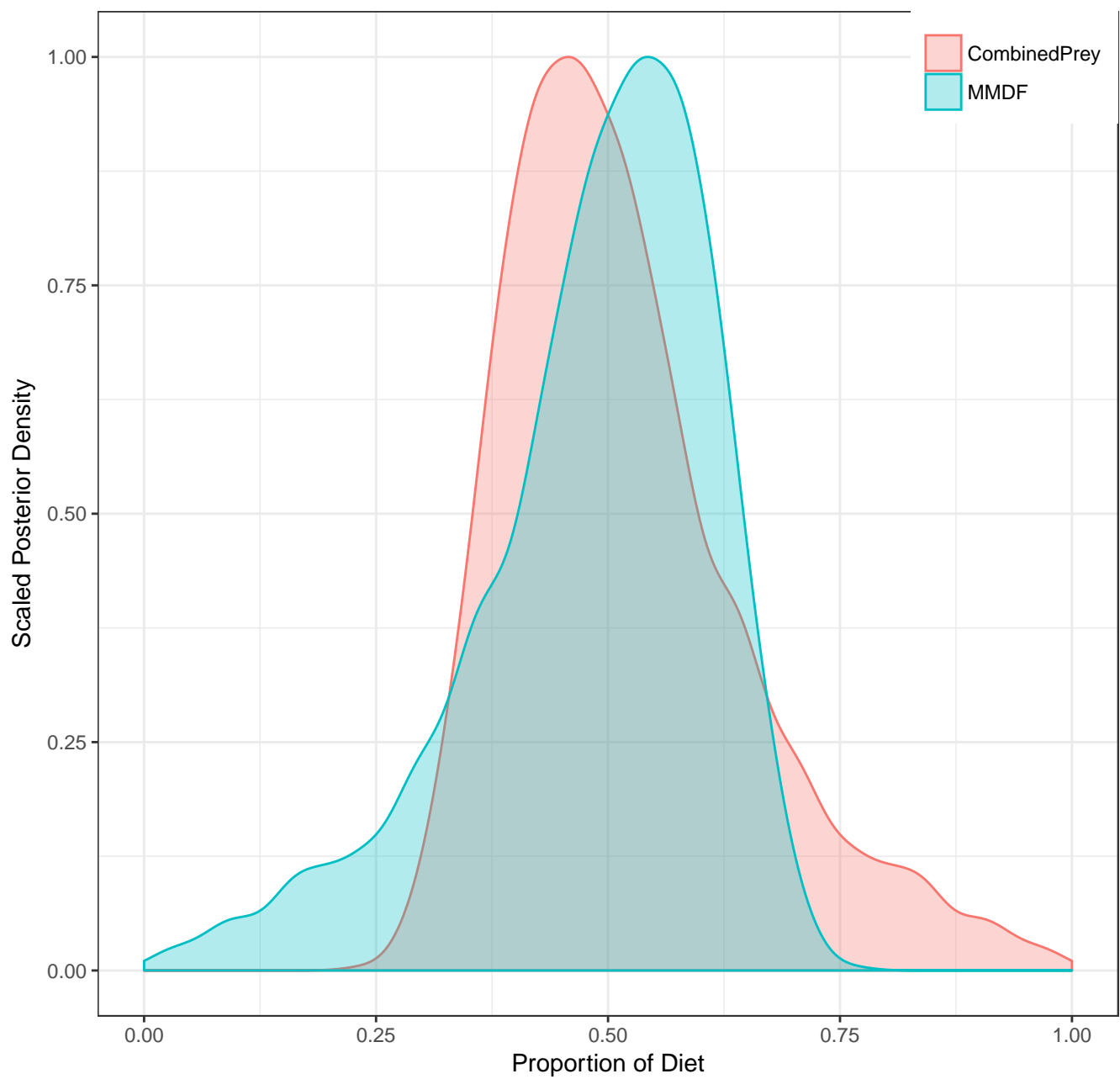

Supplement: Supplemental Information 3 [file peerj-08-8337-s008.zip › Scaled posterior density charts/Joey_proportion combadj.pdf]

# Overall Population

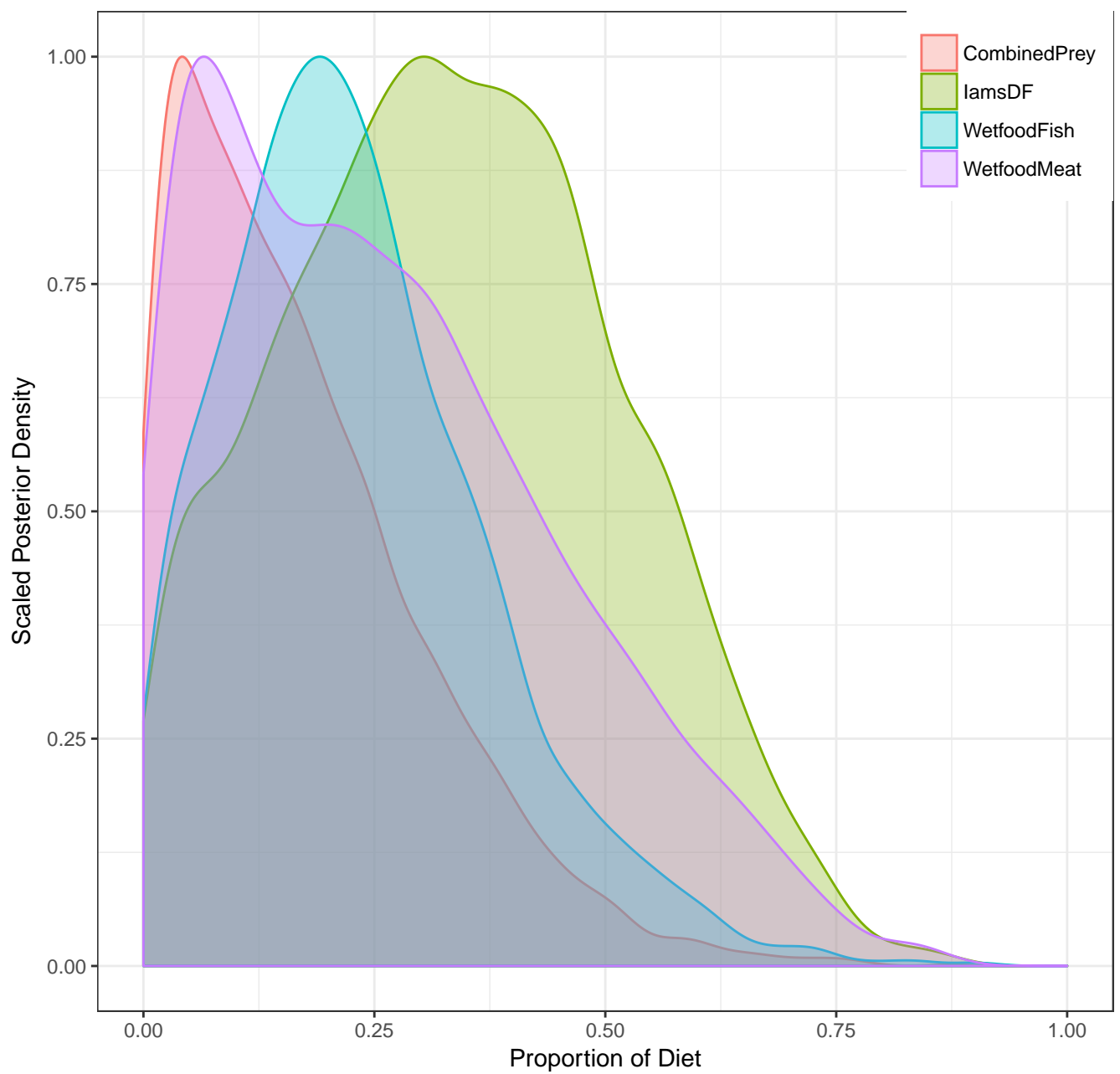

Supplement: Supplemental Information 3 [file peerj-08-8337-s008.zip › Scaled posterior density charts/Kicky_proportion combadj.pdf]

Overall Population

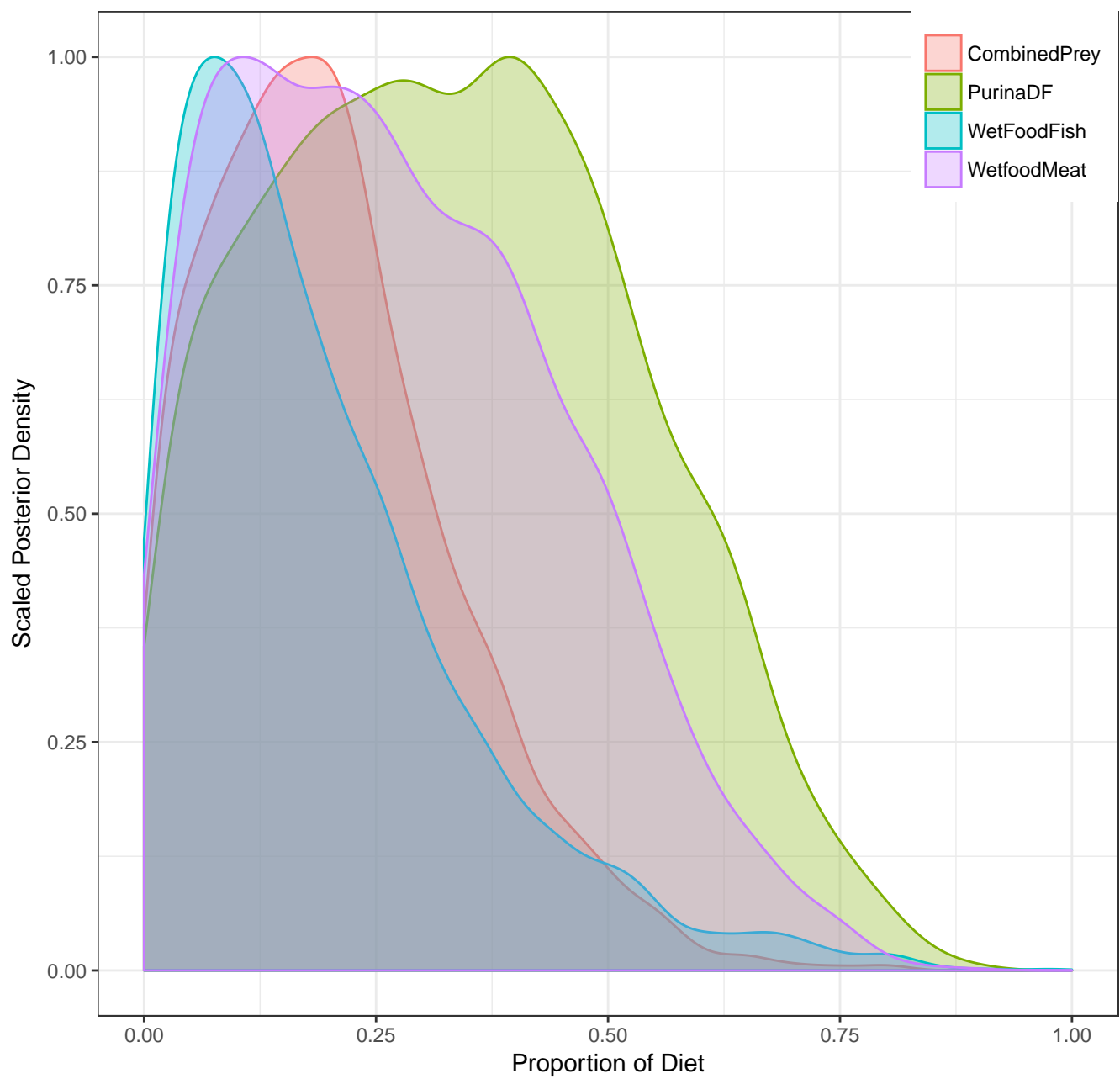

Supplement: Supplemental Information 3 [file peerj-08-8337-s008.zip › Scaled posterior density charts/Leopard_proportion combadj.pdf]

# Overall Population

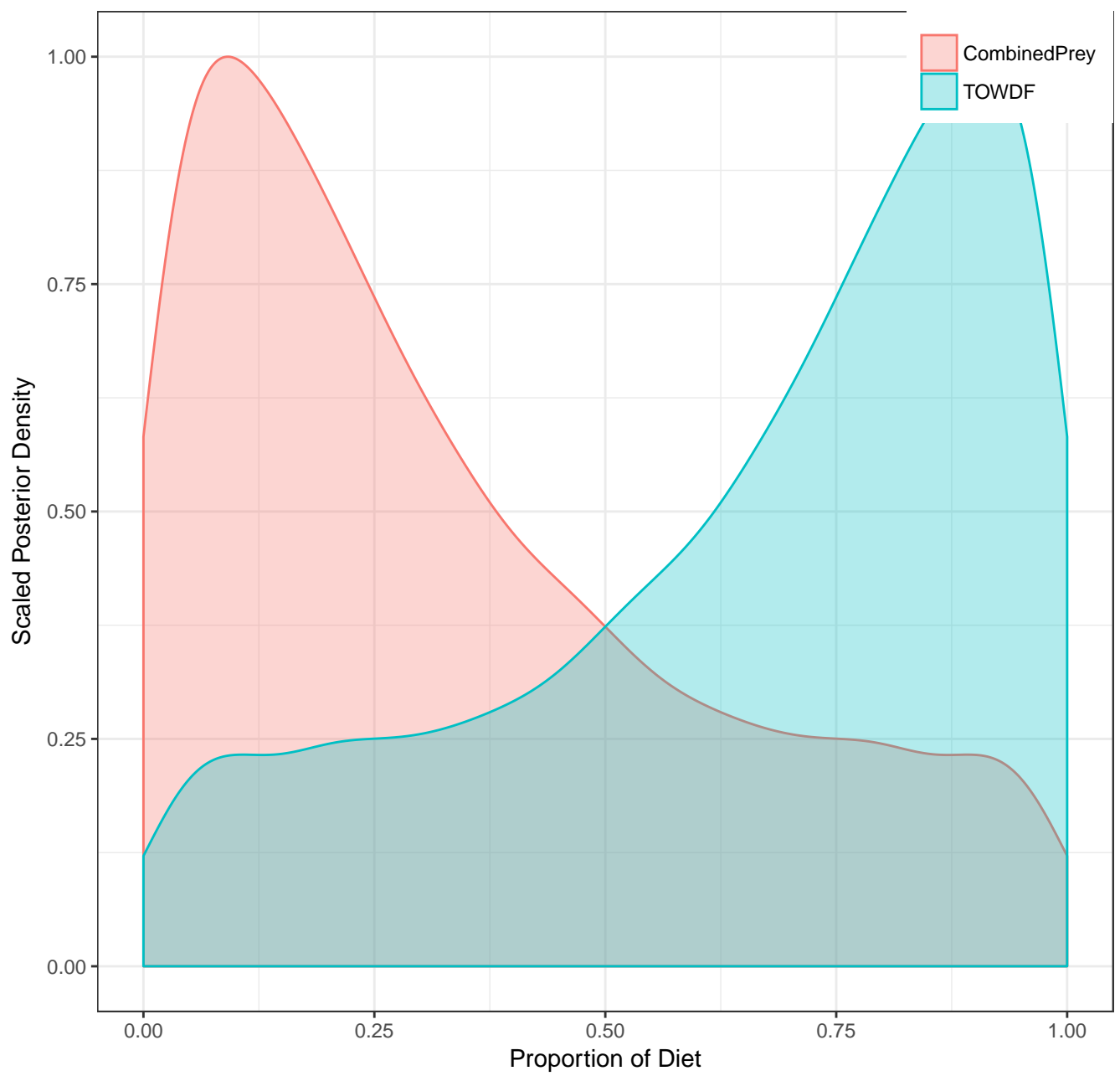

Supplement: Supplemental Information 3 [file peerj-08-8337-s008.zip › Scaled posterior density charts/Luna_proportion combadj.pdf]

# Overall Population

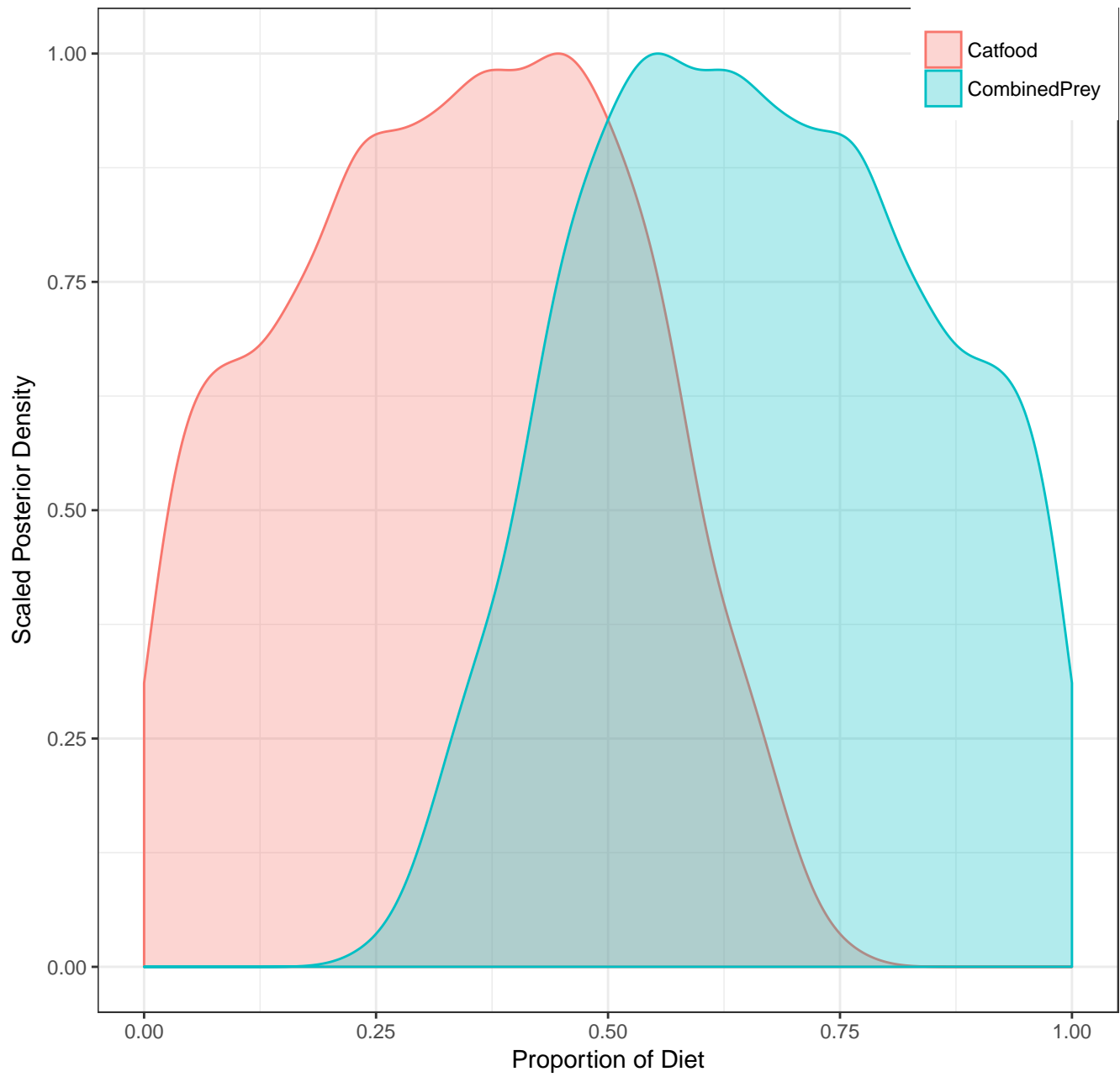

Supplement: Supplemental Information 3 [file peerj-08-8337-s008.zip › Scaled posterior density charts/Nola_proportion combadj.pdf]

# Overall Population

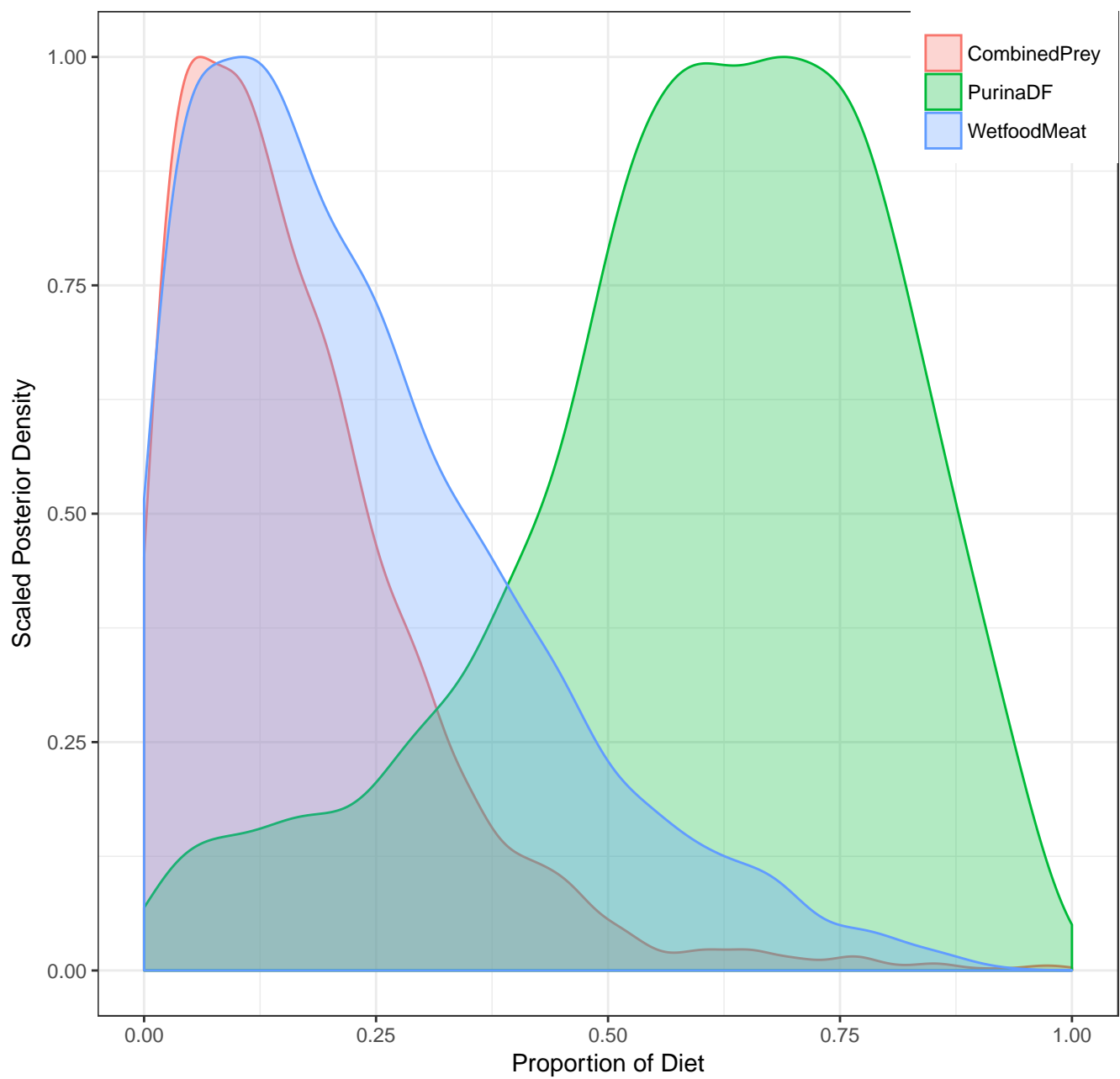

Supplement: Supplemental Information 3 [file peerj-08-8337-s008.zip › Scaled posterior density charts/Oreo_proportion combadj.pdf]

# Overall Population

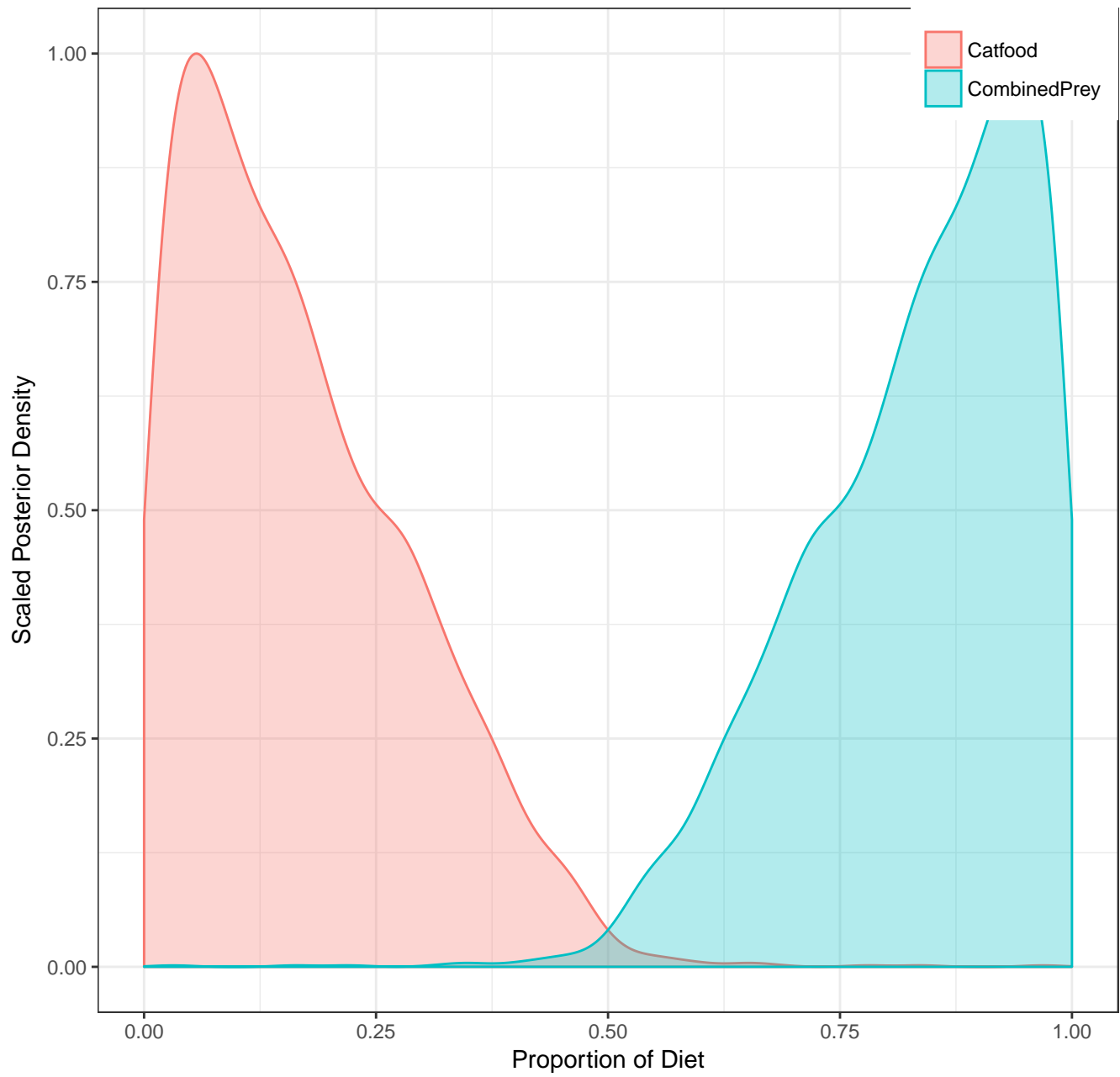

Supplement: Supplemental Information 3 [file peerj-08-8337-s008.zip › Scaled posterior density charts/Pancho_proportion combadj.pdf]

# Overall Population

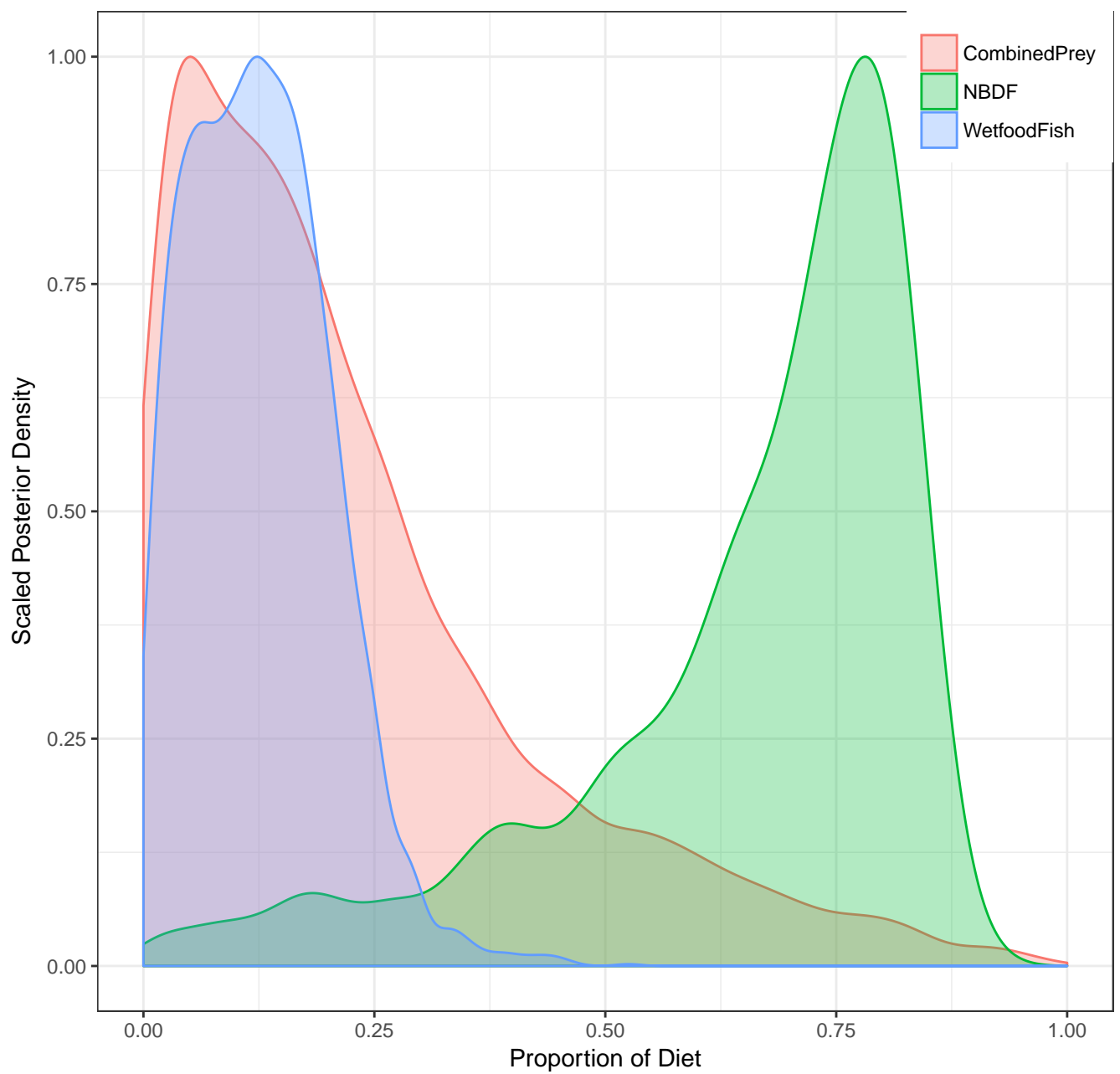

Supplement: Supplemental Information 3 [file peerj-08-8337-s008.zip › Scaled posterior density charts/Selkie_proportion combadj.pdf]

# Overall Population

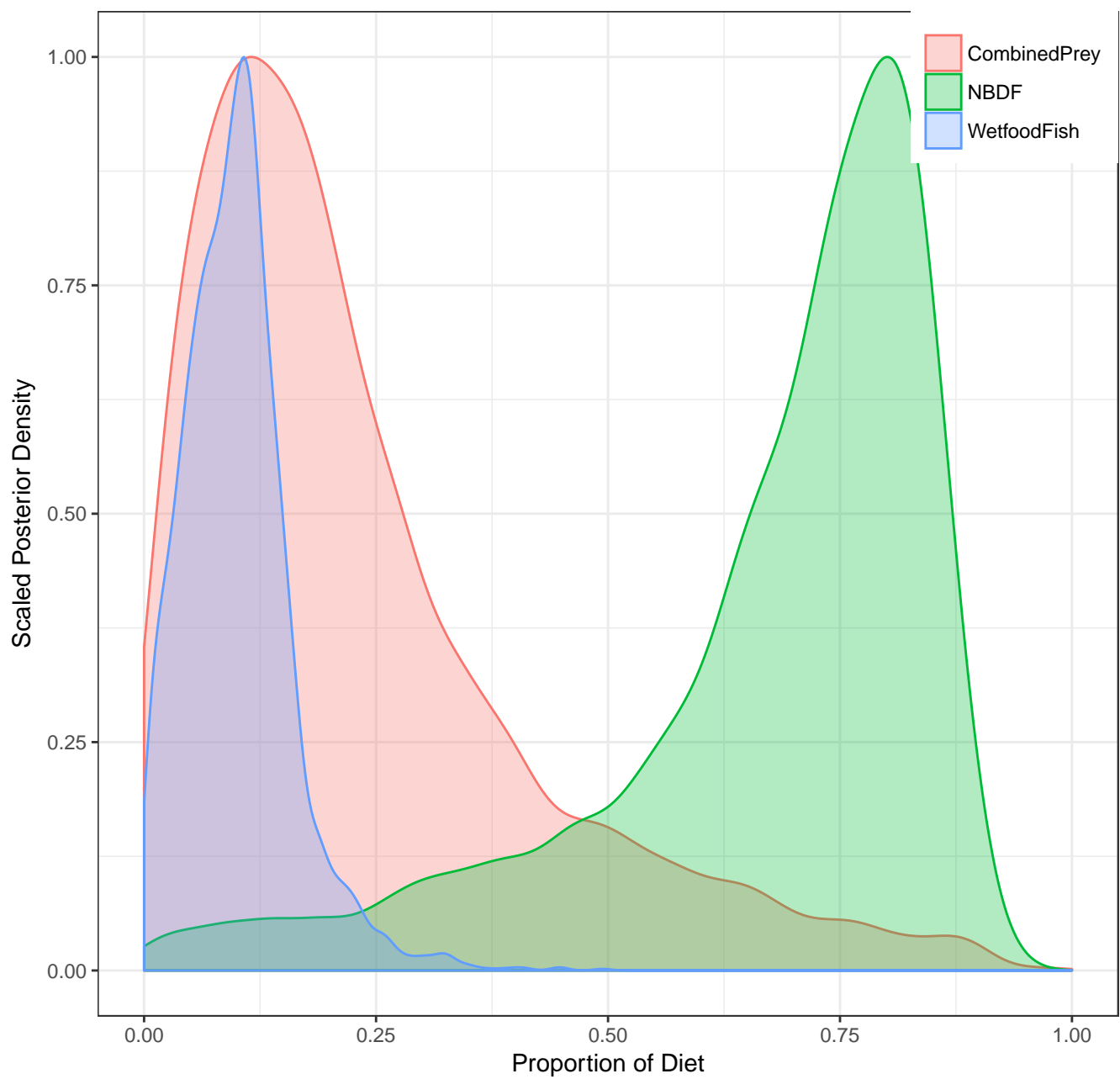

Supplement: Supplemental Information 3 [file peerj-08-8337-s008.zip › Scaled posterior density charts/Smattie_proportion combadj.pdf]

# Overall Population

Scaled Posterior Density

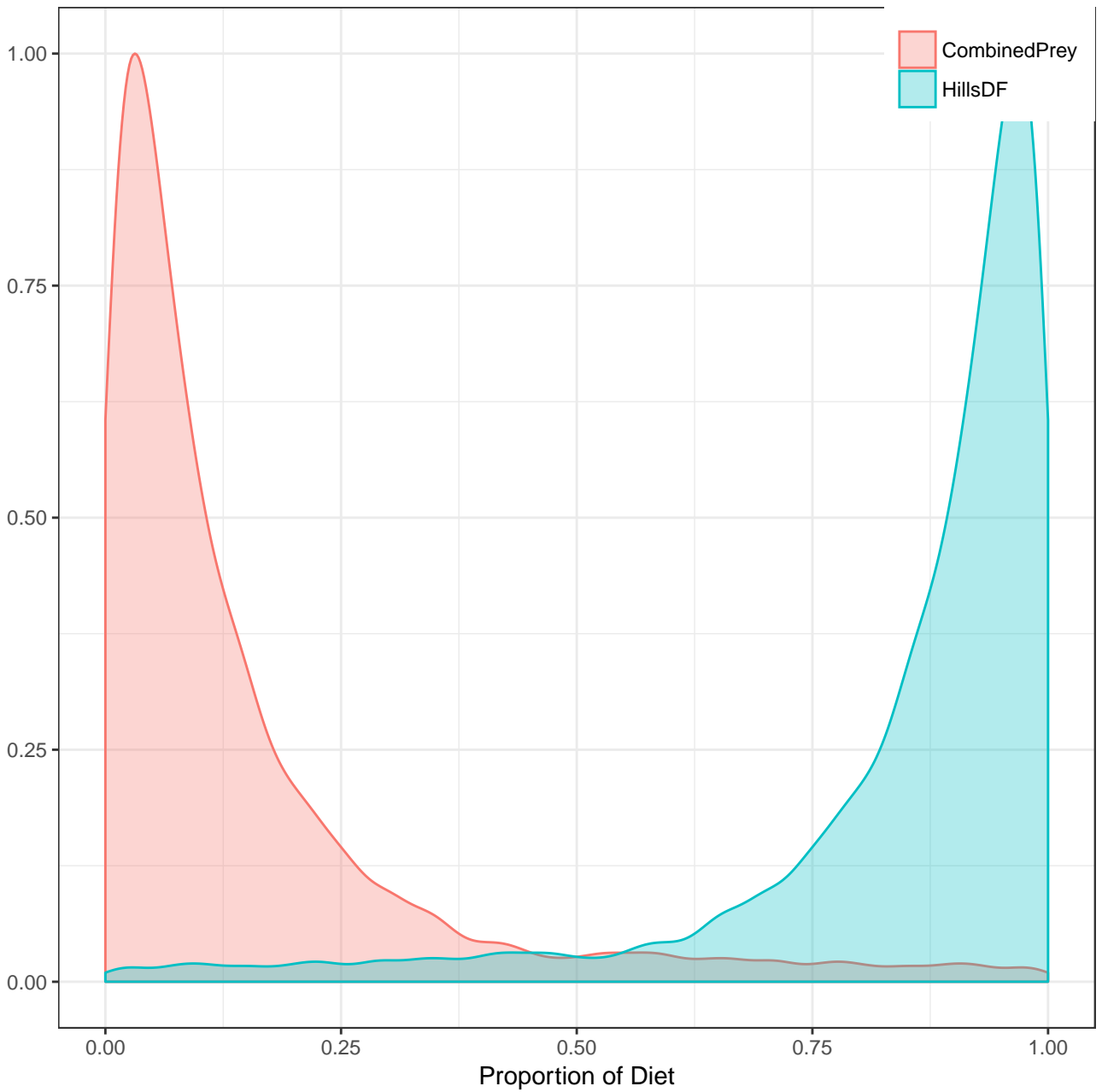

Supplement: Supplemental Information 3 [file peerj-08-8337-s008.zip › Scaled posterior density charts/Snickerdoodle_proportion combadj.pdf]

# Overall Population

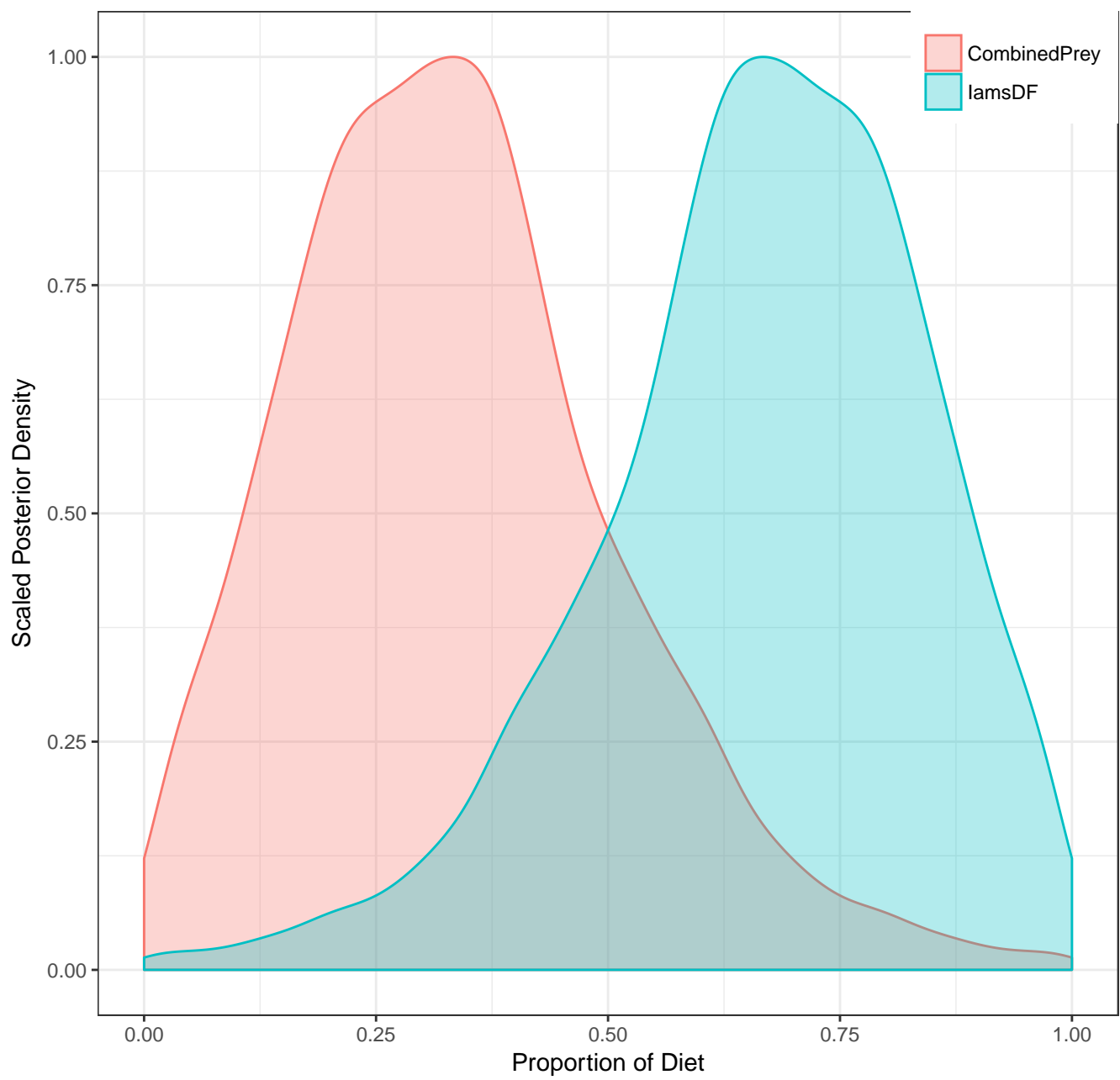

Supplement: Supplemental Information 3 [file peerj-08-8337-s008.zip › Scaled posterior density charts/SunnyB_proportion combadj.pdf]

# Overall Population

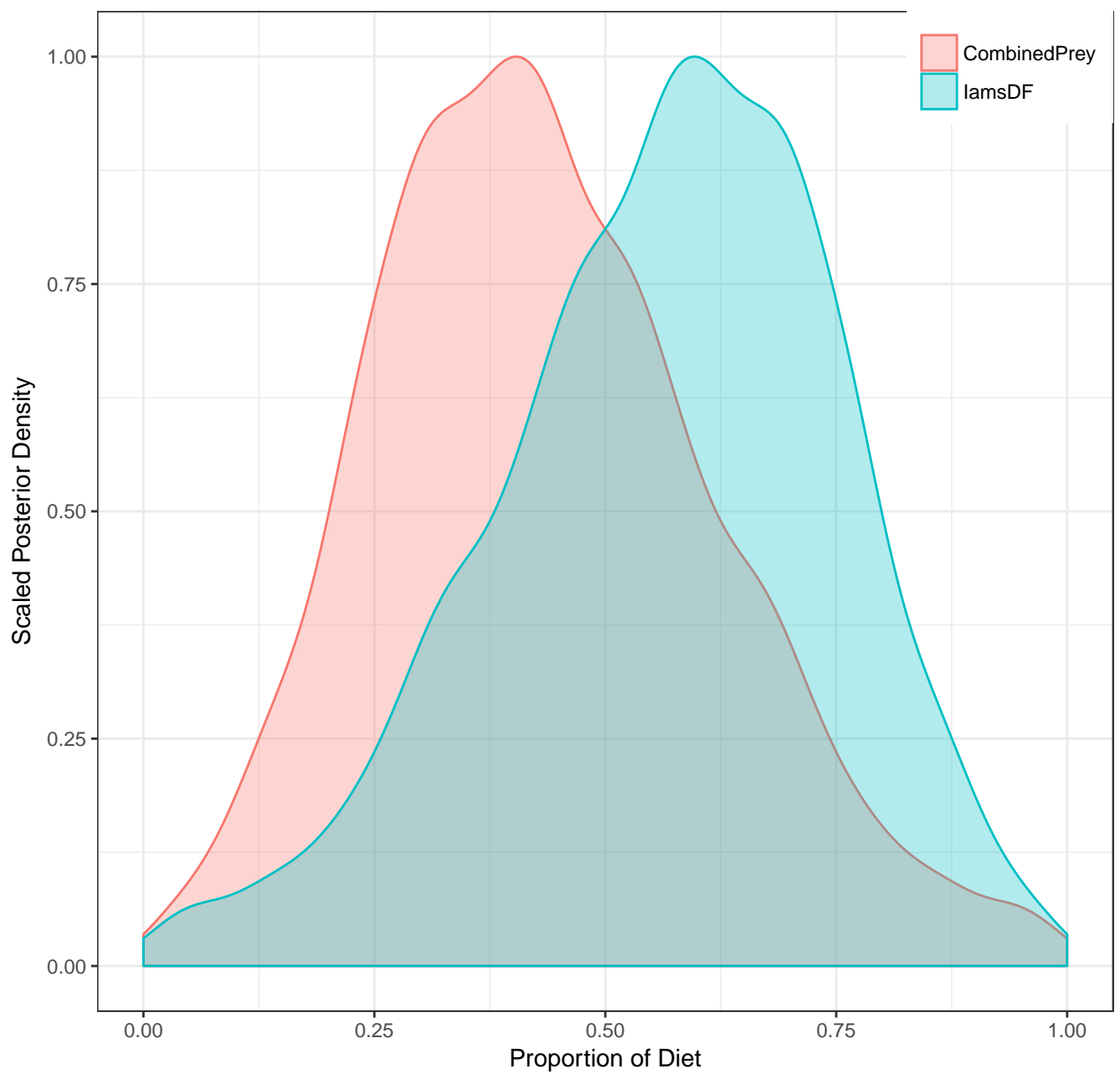

Supplement: Supplemental Information 3 [file peerj-08-8337-s008.zip › Scaled posterior density charts/SunnyZ_prportion combadj.pdf]

# Overall Population

Scaled Posterior Density

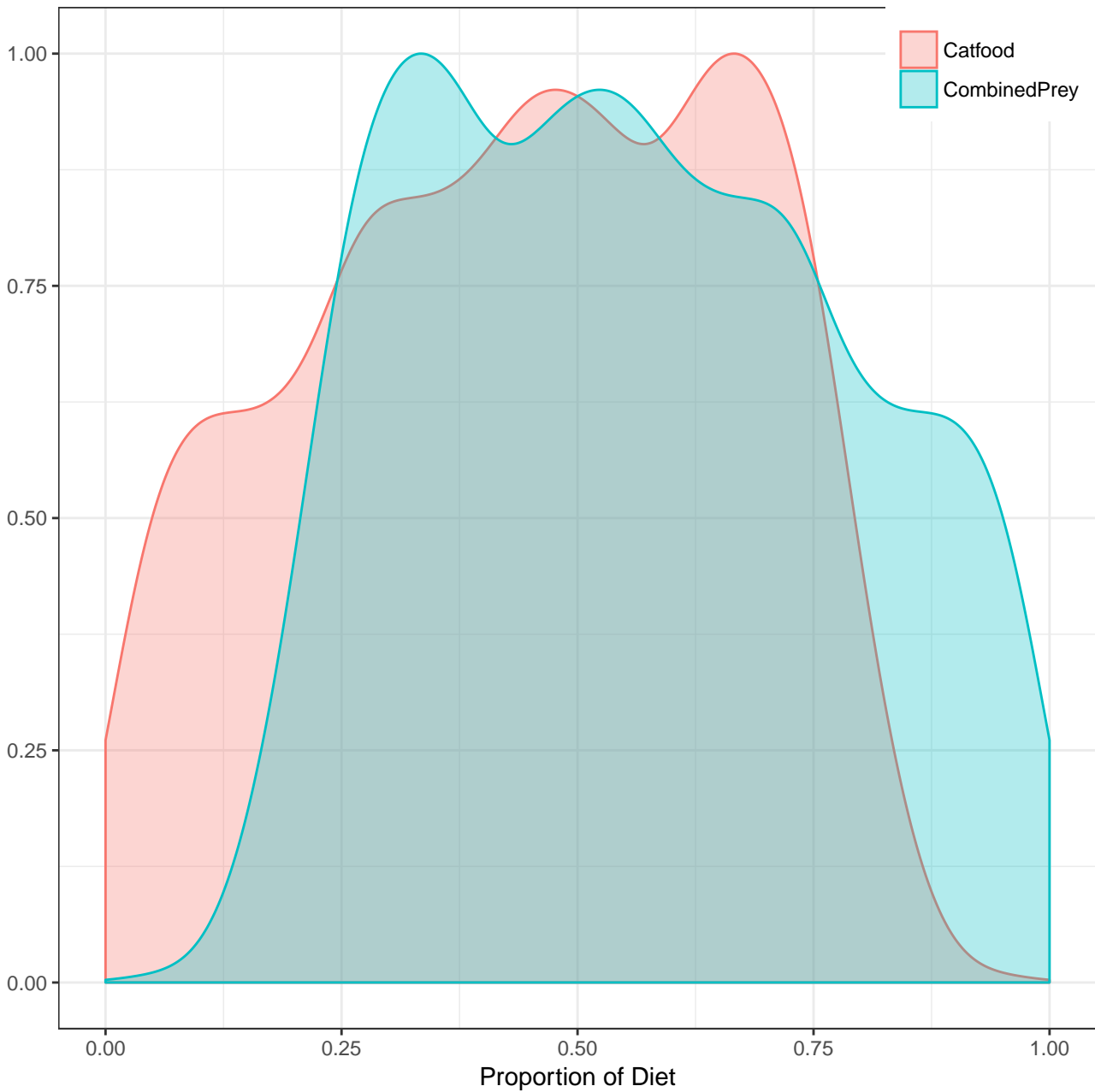

Supplement: Supplemental Information 3 [file peerj-08-8337-s008.zip › Scaled posterior density charts/Tara_proportion combadj.pdf]

# Overall Population

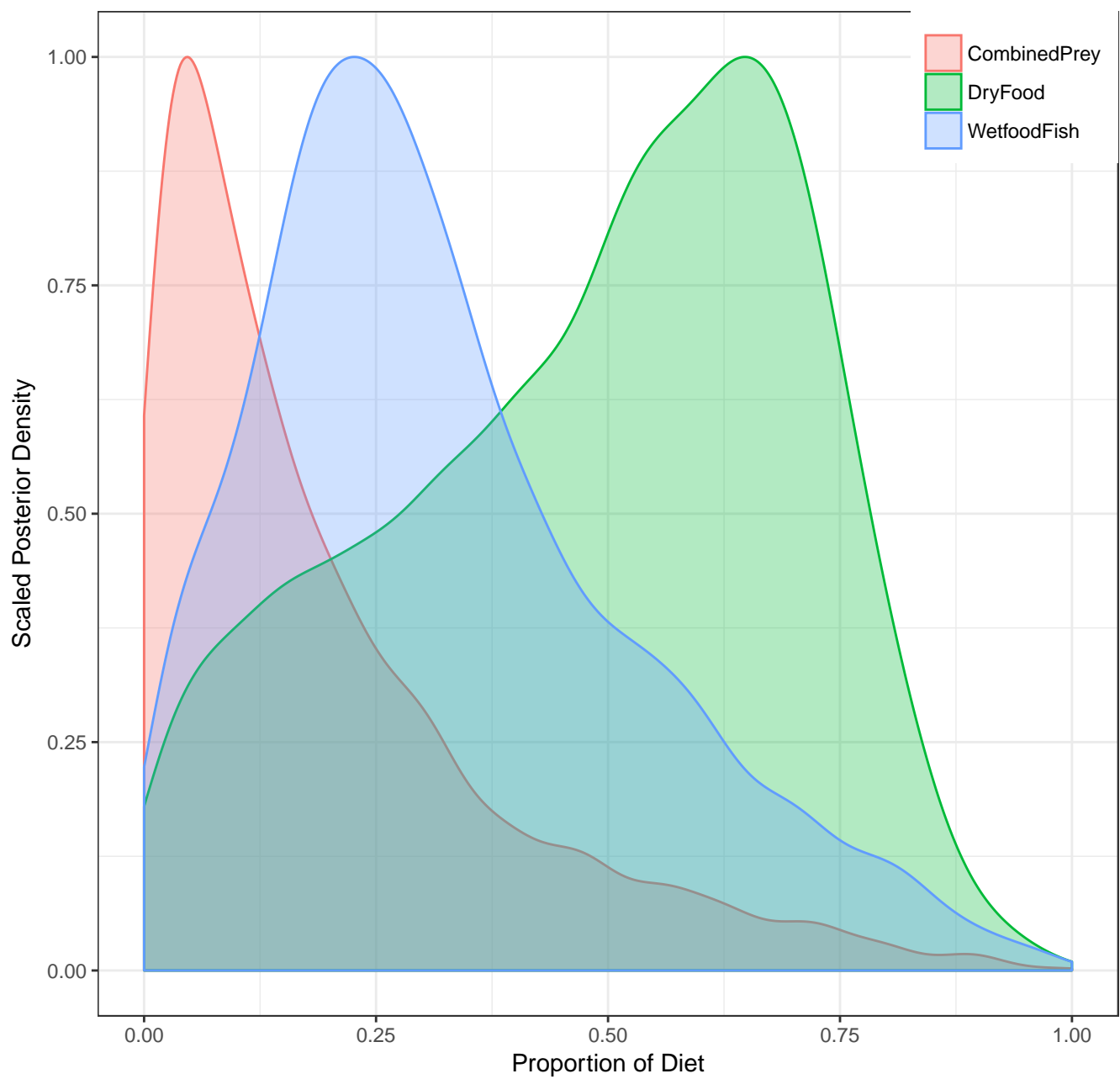

Supplement: Supplemental Information 3 [file peerj-08-8337-s008.zip › Scaled posterior density charts/Tex_proportion combadj.pdf]

# Overall Population

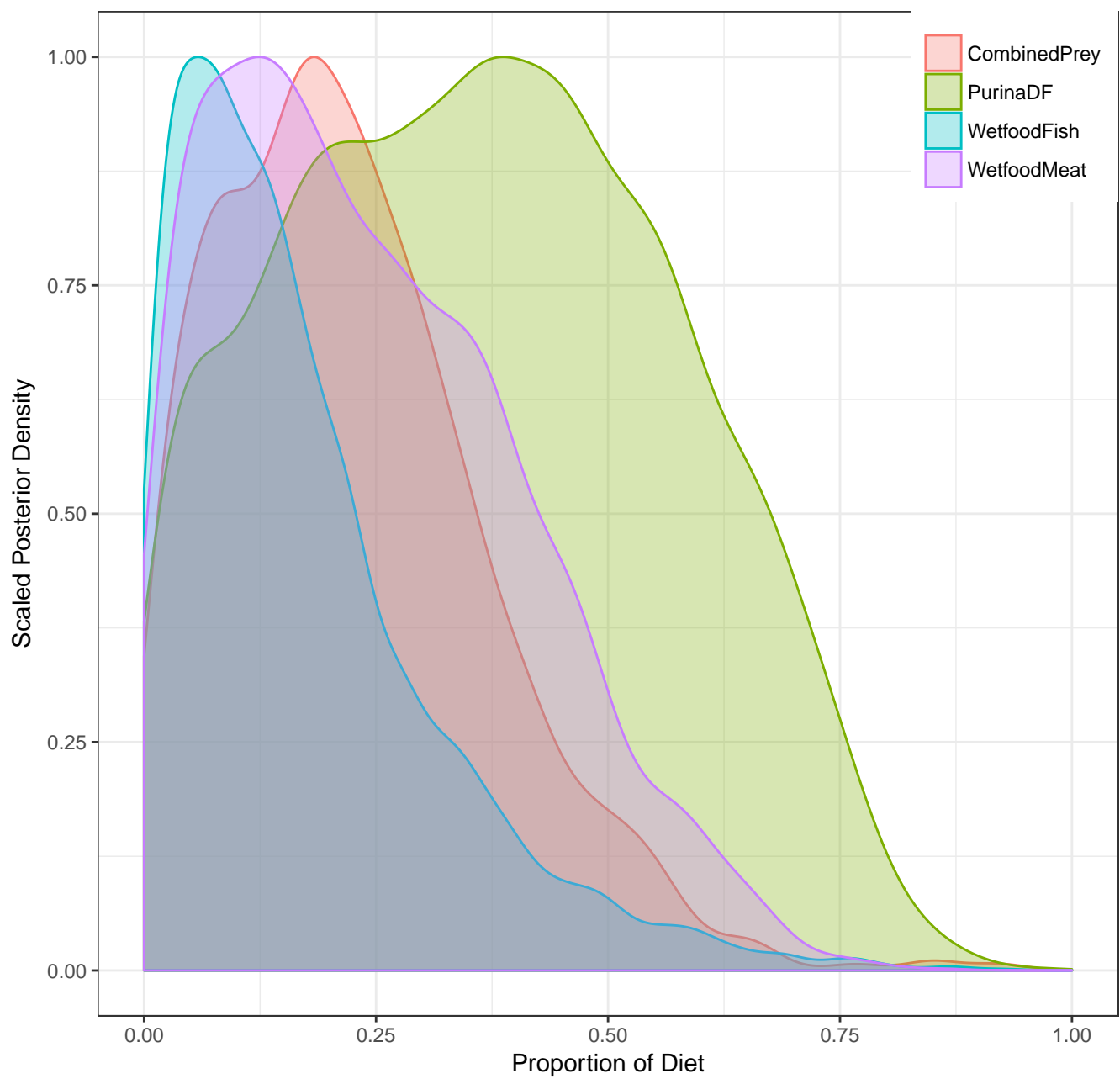

Supplement: Supplemental Information 3 [file peerj-08-8337-s008.zip › Scaled posterior density charts/TonkaA_proportion combadj.pdf]

# Overall Population

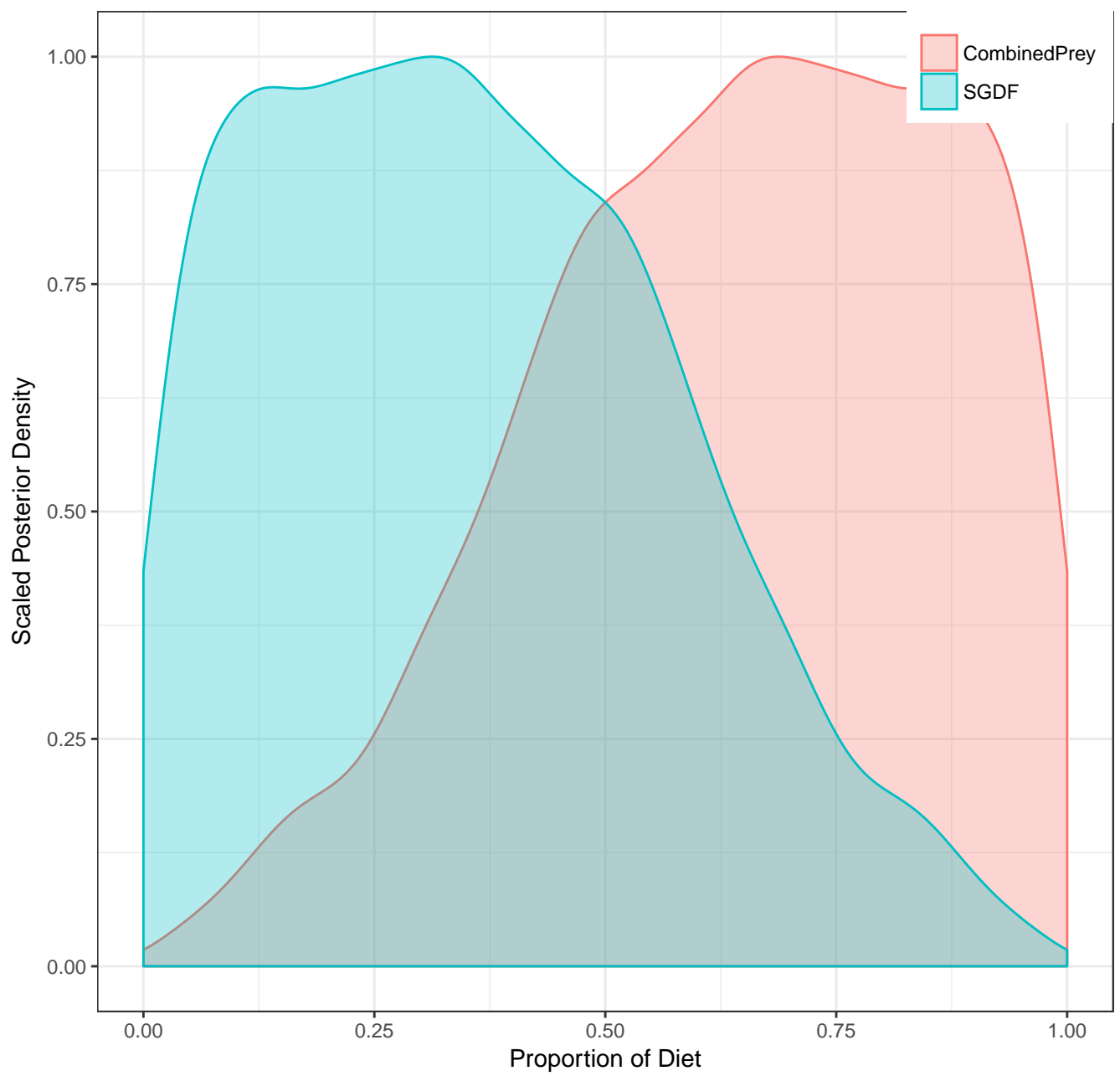

Supplement: Supplemental Information 3 [file peerj-08-8337-s008.zip › Scaled posterior density charts/Trinity_proportion combadj.pdf]

Overall Population

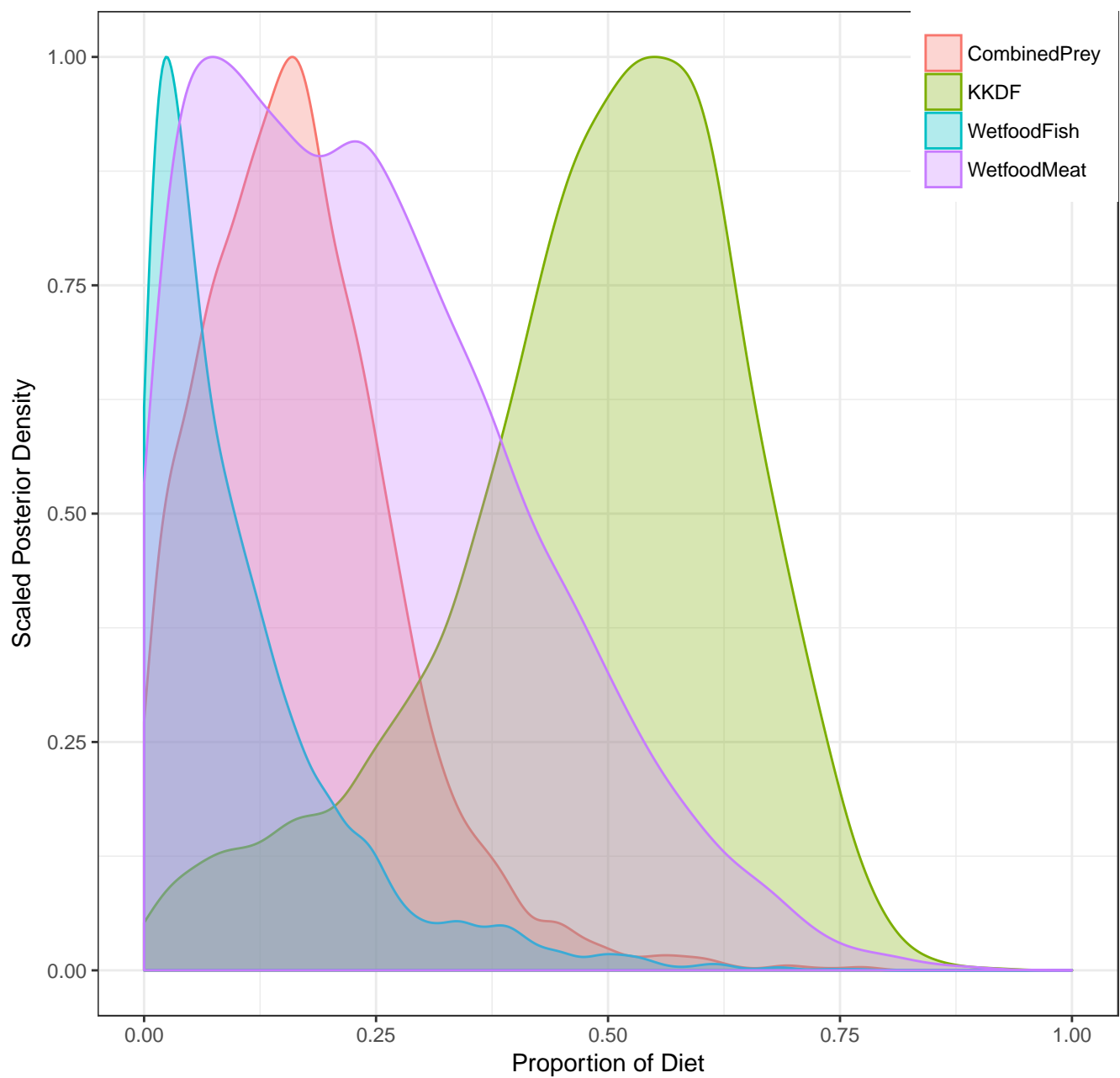

Supplement: Supplemental Information 3 [file peerj-08-8337-s008.zip › Scaled posterior density charts/Vacuum_proportion combadj.pdf]
